# The kidney failure risk equation in people with CKD and multimorbidity: the effect of competing mortality risks

**DOI:** 10.1093/ndt/gfaf252

**Published:** 2025-11-25

**Authors:** Heather Walker, Juan-Jesus Carrero, Michael K Sullivan, Ryan Field, Anne-Laure Faucon, Jennifer S Lees, Edouard L Fu, Bhautesh Dinesh Jani, Katie Gallacher, Patrick B Mark

**Affiliations:** School of Cardiovascular and Metabolic Health, University of Glasgow, Glasgow, UK; Department of Medical Epidemiology and Biostatistics, Karolinska Institutet, Stockholm, Sweden; School of Cardiovascular and Metabolic Health, University of Glasgow, Glasgow, UK; Renal and Transplant Unit, Queen Elizabeth University Hospital, NHS Greater Glasgow and Clyde, Glasgow, UK; Health Economics and Health Technology Assessment, School of Health and Wellbeing, University of Glasgow, Glasgow, UK; Department of Medical Epidemiology and Biostatistics, Karolinska Institutet, Stockholm, Sweden; School of Cardiovascular and Metabolic Health, University of Glasgow, Glasgow, UK; Renal and Transplant Unit, Queen Elizabeth University Hospital, NHS Greater Glasgow and Clyde, Glasgow, UK; Department of Clinical Epidemiology, Leiden University Medical Center, Leiden, The Netherlands; Department of Medical Epidemiology and Biostatistics, Karolinska Institutet, Stockholm, Sweden; General Practice and Primary Care, School of Health and Wellbeing, University of Glasgow, Glasgow, UK; General Practice and Primary Care, School of Health and Wellbeing, University of Glasgow, Glasgow, UK; School of Cardiovascular and Metabolic Health, University of Glasgow, Glasgow, UK; Renal and Transplant Unit, Queen Elizabeth University Hospital, NHS Greater Glasgow and Clyde, Glasgow, UK

**Keywords:** chronic renal failure, CKD, competing risk analysis, KFRE, prognostic model

## Abstract

**Background and hypothesis:**

Guidelines recommend using risk prediction models for predicting kidney failure in chronic kidney disease (CKD). Many people with CKD have multiple long-term conditions (multimorbidity), which influences outcomes including kidney failure and mortality. This study validated the four-variable kidney failure risk equation (KFRE) in individuals with CKD, with and without multimorbidity, comparing performance of KFRE using creatinine and cystatin C to calculate estimated glomerular filtration rate (eGFR) and updated the model to account for competing mortality risks.

**Methods:**

Observational cohort study using research-based (UK Biobank) and population-based cohorts (Stockholm Creatinine Measurements project: SCREAM). Multimorbidity was defined as two or more long-term conditions in addition to CKD. Kidney failure was defined as long-term dialysis or kidney transplantation. KFRE performance assessment included discrimination, calibration, and overall fit at 2 and 5 years. An updated model (using the same variables as KFRE) accounting for competing mortality risks was developed and validated.

**Results:**

14 998 of 24 489 individuals in UK Biobank (61.2%) and 30 147 of 42 902 individuals in SCREAM (70.3%) had multimorbidity. Discrimination of KFRE was good (area under curve $\ge $0.86 across eGFR equations in all cohorts, multimorbidity groups and time horizons). Kidney failure risk was under-estimated in people with multimorbidity in UK Biobank (observed/expected (O/E) ratio 1.75 at 5 years; eGFR creatinine). Conversely, calibration-in-the-large (O/E ratio) at 5 years in SCREAM was 1.05 in the multimorbidity group (eGFR creatinine). Using cystatin C compared to creatinine did not improve model performance.

Cumulative incidence of death was higher with multimorbidity compared to no multimorbidity. An updated model considering competing mortality improved calibration performance over KFRE, O/E ratio 0.98 in multimorbidity group of the validation cohort (UK Biobank) at 5 years.

**Conclusion:**

Competing mortality risk is important when predicting kidney failure, particularly for people with multimorbidity. An updated model accounting for competing mortality risk, permits improved model performance.

## Graphical Abstract

**Graphical Abstract:**
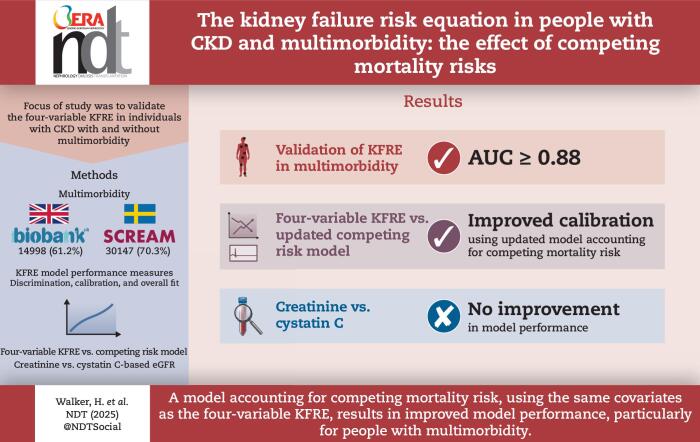

KEY LEARNING POINTS **What was known:**

- The kidney failure risk equation has been recommended in clinical guidance to predict risk of kidney failure in chronic kidney disease.
- However, there is a lack of validation in individuals with multimorbidity compared to no multimorbidity, where risk of kidney failure and competing mortality risk is likely to be elevated.

**This study adds:**

- KFRE has good discrimination but demonstrated suboptimal calibration in individuals with CKD and multimorbidity.
- An updated model accounting for competing mortality risk, using the same variables as KFRE, performs better for individuals with multimorbidity.

**Potential impact:**

- Competing mortality risk is important when predicting kidney failure, particularly for people with multimorbidity.
- By more accurately identifying those at greatest risk of developing kidney failure, a model accounting for competing mortality risk may impact individualized referral of individuals with multimorbidity and CKD, and subsequently impact treatment burden.

## INTRODUCTION

Chronic kidney disease (CKD) is a global healthcare problem [1]. A small proportion of people with CKD develop kidney failure requiring kidney replacement therapy (KRT). Individuals with CKD are also at increased risk of mortality compared to individuals without CKD [2], a potential competing risk of developing kidney failure. Prognostic models to predict the risk of kidney failure may help provide personalized healthcare aiding communication with patients [3], guiding referrals to specialist kidney care [4] and directing KRT preparation. The four-variable kidney failure risk equation (KFRE) [5], comprised of age, sex, estimated glomerular filtration rate (eGFR), and urine albumin-creatinine ratio (uACR), is the most widely used and validated kidney failure prognostic model for individuals with CKD.

Individuals with CKD have more long-term conditions (multimorbidity) compared to those without CKD [6]. Although prediction models such as KFRE are recommended for use in CKD [7], there is a lack of reporting of multimorbidity prevalence in studies that developed KFRE. No studies assess model performance in individuals with multimorbidity [8].

Certain combinations of conditions (or ‘clusters’) of multimorbidity are at higher risk of progressive kidney dysfunction [9]. Whether multimorbidity clusters affect KFRE performance is uncertain. Individuals with CKD and multimorbidity are at also at increased risk of mortality [10]. Current KFRE models used in clinical practice do not account for competing risk of mortality [11], which becomes increasingly relevant over longer prediction intervals and is more likely to affect individuals with multimorbidity [12]. It is important to determine how mortality risk influences estimated kidney failure risk for patients with CKD and multimorbidity [11, 13].

Creatinine is the most commonly used biomarker to estimate GFR in routine clinical practice. Creatinine-based estimated glomerular filtration rate (eGFR) may not provide optimal accuracy in individuals with multimorbidity or ageing, due to loss of muscle mass that affects creatinine levels [14, 15]. Subsequently, multimorbidity may impact the accuracy of creatinine-based eGFR and bias the prognostic utility of KFRE. Cystatin C is an alternative biomarker that is recommended in the Kidney Disease: Improving Global Outcomes (KDIGO) 2024 CKD clinical practice guidance [16] to be used when available for adults at risk of CKD and in certain clinical situations such as extremes of body habitus. Cystatin C is proposed as a more useful biomarker addressing risk of adverse outcomes, particularly in older patients [17, 18]. Both biomarkers are affected by differing non-GFR related factors: creatinine varies by age, sex, muscle mass, and drugs that impact tubular secretion [19], whereas cystatin C can fluctuate with chronic inflammation, smoking, increased adiposity [20], and thyroid dysfunction [21]. The use of eGFR cystatin C (eGFRcys) or combined creatinine-cystatin C (eGFRcr-cys), in place of eGFR creatinine (eGFRcr) in the KFRE, has not been explored for patients with multimorbidity.

We study the four-variable KFRE in individuals with and without multimorbidity, while assessing impact of competing mortality risks on model performance. We test whether a model accounting for competing risk of mortality performed better in individuals with multimorbidity than current KFRE. Analyses also address the influence of creatinine compared to cystatin C based eGFR on performance of KFRE.

## MATERIALS AND METHODS

### Data sources and study populations

Data were established from two databases: (i) the UK Biobank, a prospective research cohort recruiting approximately half a million participants aged 37–73 years between 2006 and 2010 with sociodemographic, medical and biological data [22]. Participants consented to data linkage to healthcare records and national death registers. Ethical approval was granted from the NHS National Research Ethics Service (16/NW/0274). (ii) The Stockholm Creatinine Measurements project (SCREAM), a population-based cohort covering the Stockholm region of Sweden with linked anonymized health and administrative data for ∼2.9 million people between 2006 and 2021 [23]. The regional ethical review board in Stockholm approved the study (reference 2017/793-31); informed consent was waived as data were deidentified.

The study observation period in UK Biobank was date of recruitment until 31 March 2020 in Scotland and England and 28 February 2018 in Wales; and in SCREAM was between 1 January 2010 and 31 December 2021. Individuals were followed up for 2 and 5 years from the index date (recruitment date in UK Biobank or date of latest sample date (creatinine/cystatin C or quantifiable albuminuria/proteinuria) in SCREAM), or until date of kidney failure or death, whichever occurred earlier.

The four-variable KFRE, containing age, sex, eGFR, and uACR was validated in both cohorts. Participants were included if they were aged ≥18 years and had creatinine and cystatin C measurements occurring on the same day. Where multiple creatinine and cystatin C measurements were available, the first measurement per participant was included. If multiple measurements occurred on the same day, the median value was used. Individuals were included if eGFR <60 millimetres per minute per 1.73 square metres (ml/min/1.73 m^2^) at baseline by any eGFR equation (eGFRcr, eGFRcys, or eGFRcr-cys). A single blood result was used as participants were assumed to be stable on recruitment to UK Biobank. In SCREAM, only outpatient measurements were used [24]. Participants were included if they had a quantifiable albuminuria/proteinuria measurement 12 months before or after creatinine and cystatin C measurement. Laboratory measurements outside the 0.1th and 99.9th percentiles of distribution were excluded, as they may represent laboratory error. Individuals established on KRT before the index date were excluded. When quantifiable uACR was unavailable, we estimated uACR concentrations from urine protein–creatinine ratio (uPCR) or dipstick proteinuria measurements using a validated conversion algorithm (Supplementary Text 1) derived from a meta-analysis of CKD cohorts [25], which provides biologically plausible estimates that account for non-linear associations between these measures. When undetectable uACR values were present they were imputed with 0.1 mg/mmol to allow calculation of KFRE.

eGFRcr was calculated using non-race adjusted 2009 CKD-EPI equation [26]. eGFRcys and eGFRcr-cys were measured using 2021 CKD-EPI equations [27].

### Multimorbidity and long-term conditions

Multimorbidity was defined as presence of two or more long-term conditions (LTCs) in addition to CKD. LTCs included are described elsewhere [28] and previously reported from UK Biobank [12]. Among these, 42 and 26 LTCs, in addition to CKD, were captured in UK Biobank and SCREAM respectively; limited to conditions present before the index date, with an infinite lookback window (Supplementary Table 1). The number of LTCs captured in SCREAM was limited to those that could be validated in administrative healthcare data [29]. In UK Biobank, conditions were self-reported by electronic questionnaire. In SCREAM, ICD-10 (International Classification of Diseases) codes, recorded in primary and secondary care records were used, with time windows based on validated algorithms for administrative data [29].

In addition, LTC counts were calculated (i.e. 0, 1, 2 … LTCs in addition to CKD) and multimorbidity clusters were constructed based on previous literature [9, 10, 30, 31] (cardiometabolic, complex, and mixed physical and mental health multimorbidity; described in Supplementary Table 2).

### Outcomes

Kidney failure was defined as requiring KRT within 2 and 5 years, defined as chronic dialysis or kidney transplantation. In SCREAM, KRT data were linked with the Swedish Renal Register, and hospital admission codes were used following an established UK Biobank algorithm [32]. In competing risk analyses, all-cause mortality was identified through linkage to national death registers.

### Sample size

A minimum sample size for external validation and development of a prognostic model was calculated based on published criteria, using the Cox–Snell R squared statistic and shrinkage factor of 0.9 [33, 34]. A minimum sample size of 4482 individuals with 106 kidney failure events was calculated for the 2-year period and 1884 individuals with 108 kidney failure events for the 5-year period.

### Statistical analysis

Baseline characteristics were summarized using means and standard deviations (SDs), medians and interquartile ranges (IQRs), and frequencies with percentages, where appropriate.

Predicted 2- and 5-year risks of kidney failure were calculated using the UK-calibrated version of KFRE [35]. eGFRcr was substituted for eGFRcys and eGFRcr-cys to assess model performance with alternative biomarkers. Sensitivity analysis was performed using regionally calibrated non-North American KFRE equation in the SCREAM cohort [36].

Validation of KFRE was examined in people with and without multimorbidity, across LTC counts and multimorbidity clusters, sample sizes permitting. Discrimination was assessed using time-dependent area under receiver operating characteristic curve(s) (AUC) [37] with 95% confidence intervals (CI) and Harrel’s C-index, with 95% CI estimated from the bootstrap method [38, 39]. Calibration was assessed by calibration curves [40], calibration intercept (to assess general calibration), calibration slope (assessing level of variation in model predictions) and calibration-in-the-large using the observed/expected (O/E) ratio. Prediction error and model fit was measured using the Brier score [41, 42] (to assess how close predicted probabilities are to observed outcomes) and scaled Brier score [43] (to allow model interpretation against a null model without covariates). Interpretation of statistical model performance measures is described in Supplementary Text 2.

Competing risk of death was considered because of high anticipated mortality rates amongst people with multimorbidity [11]. Predicted risks for individuals were calculated using UK-calibrated KFRE and observed risks were estimated with Aalen–Johansen estimator. Aalen–Johansen cumulative incidence curves for kidney failure and death were plotted in patients with and without multimorbidity. Individuals were categorized into risk groups (KFRE risk <3%, 3%–<5%, 5%–<15%, 15%–<25%, 25%–<50%, and ≥50%), as per previous validations [35, 36]. Aalen–Johansen cumulative incidence curves were plotted by risk groups and compared to Kaplan–Meier curves to depict kidney failure risk when accounting for or not accounting for competing risks.

To account for competing risks, cause-specific, Cox proportional hazards, regression modelling was used to fit the same covariates used in the four-variable KFRE in the SCREAM cohort. Proportional hazard (PH) assumptions were assessed visually and numerically. Scaled Schoenfeld residual plots for both kidney failure and mortality appeared generally stable, except for eGFR in the kidney failure model. However, numerical tests demonstrated that all covariates for the kidney failure model and age and eGFR for the mortality model showed statistically significant violations of the PH assumption. Despite these violations, we continued with the cause-specific Cox proportional hazards model using the same covariates used in the four-variable KFRE, as the magnitude of the violations was modest and the model provides interpretable hazard ratios for the effects of covariates, with a focus of risk estimation at a single time point (5 years). Furthermore, sensitivity analysis using a Fine–Gray competing risk model was performed for calibration [44], as this is less influenced by PH assumption. For the Fine–Gray model, we formally assessed the proportional subdistribution hazards assumption both visually and numerically, using score tests based on modified Schoenfeld residuals. The proportional subdistribution hazards test indicated modest departures from proportionality, with eGFR showing the strongest evidence of time variation. However, numerical tests demonstrated that age, eGFR, and log uACR for the kidney failure model and all covariates for the mortality model showed statistically significant violations of the PH assumption.

The competing risk model was then externally validated in UK Biobank. Discrimination, calibration plots, and model fit were calculated for the competing risk model [44] at 5 years and compared by presence or absence of multimorbidity. Comparison of baseline characteristics was made between individuals with 5-year risk of kidney failure >5% (recommended threshold in guidelines for referral to kidney care services [7] as calculated by KFRE and by the competing risk model). Sensitivity analysis reporting additional performance measures: precision, recall, F1 score, and area under the precision-recall curve was performed for each cohort, subgroups and time horizon to focus on assessing model performance in imbalanced datasets [45].

All analyses were performed using R (version 4.3.0) with tidyverse, nephron, dplyr, pROC, ggplot2, pmsampsize, predtools, magrittr, rms, survival, lubridate, sjPlot, survAUC, survminer, qwraps2, riskRegression, grid, gridExtra, geepack, kableExtra, boot, Hmisc, pseudo, pec, margins, lmtest, RColorBrewer, prodlim, reshape2, UpSetR, Metrics, caret, MLmetrics, PRROC, cmprsk, mstate, plotrix, knitr, splines, gtsummary, rsample, webshot, tidymodels, timeROC, crrSC, purr, and viridis packages. Results are reported in accordance with TRIPOD guidelines (Supplementary Table 3) [46].

## RESULTS

### Baseline characteristics

In total, 24 489 individuals from UK Biobank and 42 902 individuals from SCREAM were included with mean age 62.8 years (SD 5.6) and 70.1 years (SD 14.1). Of them, 54% and 66% were female, respectively (Table 1). Supplementary Figures 1 and 2 represent participant selection and exclusions.

**Table 1:** Baseline characteristics.

	UK Biobank			SCREAM		
	Whole cohort	No multimorbidity	Multimorbidity	Whole cohort	No multimorbidity	Multimorbidity
^a^	(*N* = 24 489)	(*n* = 9 491)	(n = 14 998)	(N = 42 902)	(n = 12 755)	(n = 30 147)
Age (years)	62.8 ± 5.6	62.2 ± 6.0	63.3 ± 5.3	70.1 ± 14.1	62.4 ± 16.1	73.4 ± 11.7
Creatinine (µmol/l)	97.8 ± 38.5	95.6 ± 31.6	99.2 ± 62.2	121.2 ± 59.1	121.9 ± 62.5	121.0 ± 57.6
Cystatin C (mg/l)	1.3 ± 0.3	1.2 ± 0.3	1.4 ± 0.3	1.6 ± 0.7	1.4 ± 0.6	1.7 ± 0.7
eGFRCr (ml/min/1.73 m^2^)	65.6 ± 16.0	66.3 ± 15.0	65.2 ± 16.5	50.8 ± 20.1	50.6 ± 17.9	50.9 ± 21.0
eGFRCys (ml/min/1.73 m^2^)	55.6 ± 13.1	59.6 ± 13.9	53.1 ± 11.9	45.5 ± 20.1	55.9 ± 22.9	41.1 ± 17.0
eGFRCr-Cys (ml/min/1.73 m^2^)	62.4 ± 11.1	65.0 ± 9.5	60.8 ± 11.6	49.4 ± 17.5	55.1 ± 17.5	47.0 ± 16.9
uACR (mg/mmol)	5.4 ± 32.4	3.3 ± 18.8	6.7 ± 38.5	15.8 ± 46.0	12.6 ± 39.5	17.1 ± 48.4
Median (IQR)	0.0 (0.0, 1.5)	0.0 (0.0, 1.0)	0.5 (0.0, 1.9)	0.9 (0.0, 7.6)	0.4 (0.0, 5.7)	1.3 (0.0, 7.6)
Median (IQR)^b^	1.5 (0.8, 4.2)	1.3 (0.7, 3.0)	1.7 (0.9, 4.9)	5.3 (1.2, 22.8)	3.7 (0.9, 17.4)	5.8 (1.4, 7.6)
Sex *n* (%)						
Male	11 232 (45.9)	4 249 (44.8)	6 983 (46.6)	14 552 (33.9)	2 765 (21.7)	11 787 (39.1)
Female	13 258 (54.1)	5 242 (55.2)	8 015 (53.4)	28 350 (66.1)	9 990 (78.3)	18 360 (60.9)
LTC count						
Median (IQR)	2 (1, 3)	1 (0, 1)	3 (2, 4)	2 (1, 4)	1 (0, 1)	3 (2, 4)
Multimorbidity cluster, *n* (%)						
Cardiometabolic	50 375 (21.9)	N/A	50 375 (35.8)	17 854 (41.6)	N/A	17 854 (59.2)
Complex	60 018 (24.6)	N/A	60 018 (40.1)	16 330 (38.1)	N/A	16 330 (54.2)
Mixed mental/physical	10 967 (8.0)	N/A	10 967 (13.1)	60 175 (14.4)	N/A	60 175 (20.5)

^a^Mean (SD) unless otherwise specified

^b^Excluding undetectable uACR values

eGFRCr = eGFR creatinine

eGFRCys = eGFR cystatin C

eGFRCr-Cys = eGFR creatinine-cystatin C

SCREAM participants were older, had greater female predominance, lower mean eGFR measured by eGFRcr and eGFRcys, higher albuminuria, and higher prevalence of multimorbidity compared to UK Biobank participants.

In UK Biobank, 14 998 (61.2%) individuals had multimorbidity, of whom 5375 (21.9%) had cardiometabolic multimorbidity, 6018 (24.6%) complex multimorbidity, and 1967 (8.0%) mixed physical and mental health multimorbidity. In SCREAM, 30 147 (70.3%) had multimorbidity, 17 854 (41.6%) cardiometabolic multimorbidity, 16 330 (38.1%) complex multimorbidity, and 6175 (14.4%) mixed physical and mental health multimorbidity. The overlaps between the multimorbidity clusters identified were similar in UK Biobank and SCREAM (Supplementary Fig. 3). Distribution of LTCs in each cohort are depicted in Supplementary Figs 4 and 5. Individuals with multimorbidity were older, had higher levels of albuminuria, and had lower mean eGFRcys compared to those without multimorbidity, with these findings being more prominent in the SCREAM cohort. Mean eGFRcr was similar in those with and without multimorbidity in both cohorts.

### Kidney failure and mortality events

Within 2 and 5 years there were 150 (0.6%) and 312 (1.3%) kidney failure events and 499 (2.0%) and 1471 (5.8%) deaths in UK Biobank. In SCREAM there were 511 (1.2%) and 1098 (2.6%) kidney failure events and 5191 (12.1%) and 10 152 (23.7%) deaths in SCREAM, within 2 and 5 years, respectively (Table 2). Both death and kidney failure event rates (per 1000 person years) were higher in those with multimorbidity compared to those without in UK Biobank but only death events were higher in the multimorbidity group in SCREAM (Table 2).

**Table 2:** Kidney failure and death events over 2 and 5 years by cohort and multimorbidity status.

	UK Biobank			SCREAM		
	Whole cohort	No multimorbidity	Multimorbidity	Whole cohort	No multimorbidity	Multimorbidity
	(*N* = 24 489)	(*n* = 9 491)	(*n* = 14 998)	(*N* = 42 902)	(*n* = 12 755)	(*n* = 30 147)
Follow-up time (months)						
2-year cohort	23.7 ± 2.0	23.9 ± 1.5	23.6 ± 2.3	20.6 ± 6.7	21.8 ± 5.7	20.1 ± 7.1
5-year cohort	58.1 ± 8.4	58.9 ± 6.3	57.5 ± 9.5	41.3 ± 20.9	46.5 ± 19.1	39.1 ± 21.3
Death, *n* (%)						
Within 2-year follow up	499 (2.0)	105 (1.1)	394 (2.6)	50 191 (12.1)	569 (4.5)	40 622 (15.3)
Per 1000 person years	10.3	2.3	13.3	70.5	11.5	91.5
Within 5-year follow up	10 471 (5.8)	329 (3.5)	10 142 (7.6)	10 152 (23.7)	1 287 (10.1)	80 865 (29.4)
Per 1000 person years	12.4	7.1	15.9	68.7	26.0	90.2
Kidney failure, *n* (%)						
Within 2-year follow up	150 (0.6)	29 (0.3)	121 (0.8)	511 (1.2)	167 (1.3)	344 (1.1)
Per 1000 person years	3.1	1.5	4.1	6.9	7.2	6.8
Within 5-year follow up	312 (1.3)	70 (0.7)	242 (1.6)	10 098 (2.6)	375 (2.9)	723 (2.4)
Per 1000 person years	2.6	1.5	3.4	7.4	7.6	7.4

Kidney failure and mortality events by LTC count and multimorbidity clusters are presented in Supplementary Tables 4 and 5. Based on minimum sample size calculations there were adequate kidney failure event numbers to perform validation in the following subgroups: cardiometabolic multimorbidity cluster groups in both cohorts and LTC count groups and complex multimorbidity group in the SCREAM cohort.

### Discrimination

Discrimination of KFRE was good in individuals with and without multimorbidity, for all eGFR equations tested and across the 2- and 5-year models (AUC $\ge $0.86 and c-index $\ge $0.86 in both cohorts: Tables 3 and 4 and Supplementary Tables 6 and 7).

**Table 3:** Performance measures for validation of UK-calibrated KFRE using eGFRcr equation by cohort and multimorbidity status at 2 years.

		Whole cohort		Multimorbidity		No multimorbidity	
eGFR equation used in KFRE	Performance measure for 2-year KFRE	UK Biobank	SCREAM	UK Biobank	SCREAM	UK Biobank	SCREAM
eGFRcr	AUC	0.88 (0.84–0.92)	0.89 (0.88–0.91)	0.88 (0.84–0.92)	0.90 (0.88–0.93)	0.86 (0.77–0.95)	0.94 (0.92–0.97)
	C-index	0.89 (0.86–0.92)	0.93 (0.92–0.94)	0.90 (0.87–0.93)	0.92 (0.91–0.94)	0.85 (0.75–0.93)	0.95 (0.93–0.95)
	O/E ratio	2.13 (1.97–2.29)	1.09 (1.01–1.18)	2.18 (2.01–2.36)	1.09 (0.99–1.20)	1.95 (1.59–2.31)	1.10 (0.95–1.25)
	Calibration intercept	0.19 (−0.09–0.47)	0.26 (0.16–0.35)	0.27 (−0.03–0.58)	0.25 (0.13–0.37)	−0.08 (−0.70–0.53)	0.26 (0.10–0.42)
	Calibration slope	0.69 (0.58–0.80)	1.35 (1.21–1.49)	0.67 (0.54–0.79)	1.17 (1.06–1.28)	0.83 (0.50–1.17)	1.43 (1.18–1.68)
	Brier score	0.005 (0.004–0.006)	0.009 (0.009–0.010)	0.007 (0.006–0.008)	0.010 (0.009–0.011)	0.003 (0.002–0.004)	0.009 (0.007–0.010)
	Scaled Brier score (%)	14.90 (9.52–20.37)	31.07 (28.24–33.66)	15.10 (9.36–20.60)	27.26 (23.94–30.22)	13.53 (1.57–26.62)	39.29 (33.21–44.23)

**Table 4:** Performance measures for validation of UK-calibrated KFRE using eGFRcr equation by cohort and multimorbidity status at 5-years.

		Whole cohort		Multimorbidity		No multimorbidity	
eGFR equation used in KFRE	Performance measure for 5-year KFRE	UK Biobank	SCREAM	UK Biobank	SCREAM	UK Biobank	SCREAM
eGFRcr	AUC	0.90 (0.88–0.92)	0.88 (0.87–0.90)	0.90 (0.87–0.93)	0.88 (0.86–0.90)	0.89 (0.83–0.94)	0.89 (0.86–0.91)
	C-index	0.91 (0.89–0.93)	0.92 (0.91–0.93)	0.91 (0.88–0.93)	0.91 (0.90–0.92)	0.92 (0.88–0.95)	0.94 (0.93–0.95)
	O/E ratio	1.76 (1.65–1.87)	1.08 (1.02–1.14)	1.75 (1.62–1.87)	1.05 (0.98–1.13)	1.83 (1.59–2.06)	1.15 (1.04–1.25)
	Calibration intercept	0.46 (0.28–0.64)	0.02 (−0.10–0.07)	0.40 (0.20–0.61)	−0.05 (−0.16–0.05)	0.69 (0.33–1.04)	0.05 (−0.10–0.20)
	Calibration slope	0.78 (0.71–0.86)	1.00 (0.92–1.07)	0.76 (0.68–0.85)	1.00 (0.91–1.10)	0.91 (0.73–1.10)	0.94 (0.82–1.05)
	Brier score	0.009 (0.008–0.010)	0.023 (0.021–0.024)	0.011 (0.010–0.013)	0.023 (0.021–0.025)	0.005 (0.004–0.007)	0.022 (0.020–0.024)
	Scaled Brier score (%)	30.86 (25.18–36.38)	36.57 (33.86–39.09)	31.14 (25.87–36.11)	35.32 (32.01–38.46)	29.27 (19.07–39.64)	39.49 (34.53–44.06)

Discrimination of KFRE was good across all eGFR equations for all LTC count groups (0–≥5 LTCs) in SCREAM (2-year model; AUC$\ \ge $0.88 and c-index $\ge $0.91, 5-year model; AUC$\ge $0.87 and c-index $\ge $0.91–Supplementary Tables 8 and 9), cardiometabolic multimorbidity in both cohorts (2-year model; AUC$\ \ge $0.84 and c-index $\ge $0.87, 5-year model; AUC$\ge $0.87 and c-index $\ge $0.88–Supplementary Tables 8 and 9) and complex multimorbidity in SCREAM (2-year model; AUC 0.93 and c-index $\ge $0.92, 5-year model; AUC$\ \ge $0.86 and c-index $\ge $0.91–Supplementary Tables 10 and 11).

### Calibration

**Figure 1:**
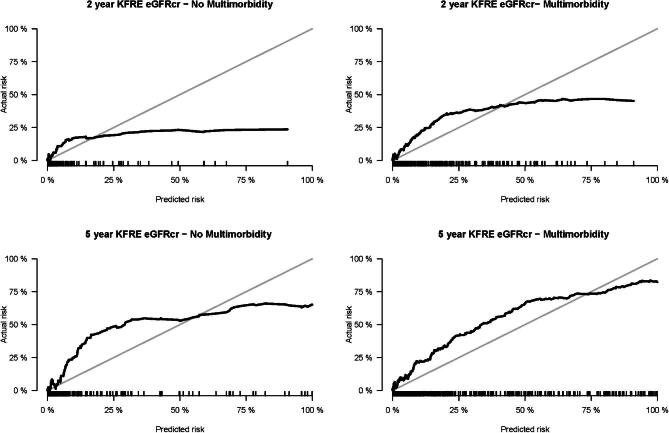
Calibration curves for predicted versus observed 2-year and 5-year risk of kidney failure by multimorbidity status in the UK Biobank cohort.

**Figure 2:**
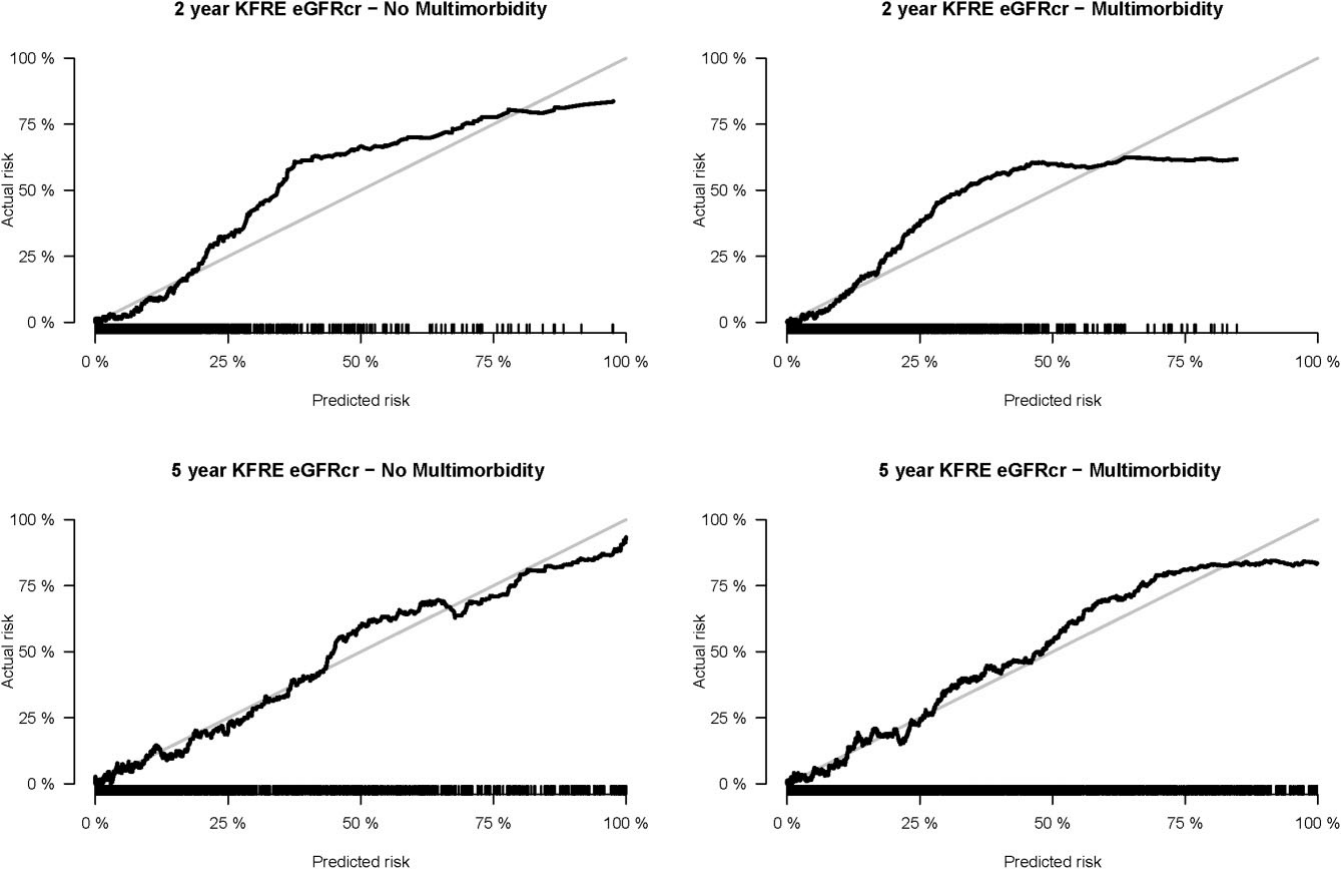
Calibration curves for predicted versus observed 2-year and 5-year risk of kidney failure by multimorbidity status in the SCREAM cohort.

Calibration assessed without accounting for competing risk of mortality was inaccurate (Figs 1 and 2, Supplementary Text 3, and Supplementary Figs 8 and 9). Calibration-in-the-large, showed underestimation of kidney failure risk (O/E ratio range 1.01–2.18), except when eGFRcys was used in the 2- and 5-year models in the SCREAM whole cohort and multimorbidity group and when eGFRcr-cys was substituted in the 5-year KFRE and validated in the multimorbidity group (Tables 3 and 4 and Supplementary Tables 6 and 7). Full calibration quantitative results are presented in Tables 3–4 and Supplementary Tables 6 and 7.

There was no improvement in calibration performance (accounting for all calibration performance measures) when using eGFRcys or eGFRcr-cys compared to eGFRcr (Tables 3 and 4, Supplementary Tables 6 and 7, Figs 1 and 2, and Supplementary Figs 6–11).

Calibration results for LTC count groups and multimorbidity clusters are presented in Supplementary Figs 12–15 and Supplementary Tables 8–11. Calibration-in-the-large and calibration slopes are similar across LTC counts but general calibration (assessed by calibration intercepts) changed from systematic underestimation of risk to overestimation of risk at 5 years as LTC counts increased. There was prominent underestimation of risk in the cardiometabolic multimorbidity cluster in UK Biobank assessed by O/E ratio (2 and 5 years).

### Overall fit

Overall fit performance measures (Tables 3 and 4 and Supplementary Tables 6 and 7) were similar across different eGFR equations but was better in those without multimorbidity compared with those with multimorbidity in both cohorts and at both time points.

Brier scores were similar across LTC count groups but scaled Brier scores were lower in higher LTC counts (3–≥5). Overall fit performance measures for multimorbidity clusters and LTC counts are presented in Supplementary Tables 8–11.

### Competing risk analysis

The 5-year cumulative incidence of kidney failure was 3.0% (95% CI: 2.7%–3.2%) in the SCREAM multimorbidity cohort, and 1.6% (95% CI: 1.4%–1.8%) in the UK Biobank multimorbidity cohort. This was compared with a 5-year cumulative incidence of kidney failure of 3.6% (95% CI: 3.2%–3.9%) and 0.7% (95% CI: 0.6%–0.9%) in the SCREAM and UK Biobank no multimorbidity cohorts, respectively (Supplementary Table 12).

**Figure 3:**
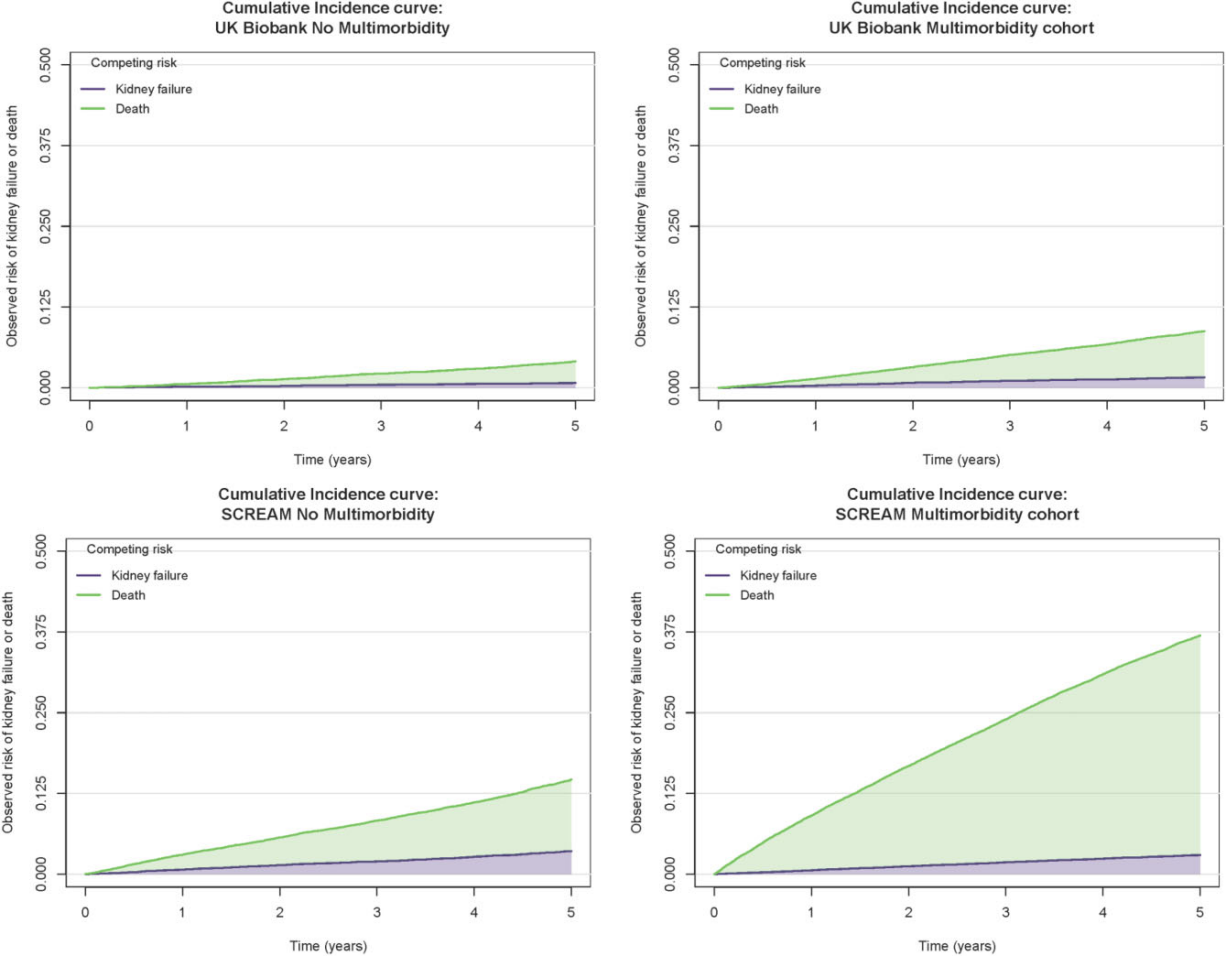
Cumulative incidence curves of kidney failure and mortality in UK Biobank and SCREAM: multimorbidity and no multimorbidity cohorts.

There was a higher risk of death in the multimorbidity group, with a prominent increase in risk of death over time compared to the no multimorbidity group (Fig. 3). The 5-year cumulative incidence of mortality was 34.0% (95% CI: 33.4%–34.6%) and 7.2% (95% CI: 6.7%–7.6%) in the SCREAM and UK Biobank multimorbidity cohorts, respectively. In comparison, the 5-year cumulative incidence of mortality in the no multimorbidity groups was lower; SCREAM 11.1% (95% CI: 10.5%–11.7%) and UK Biobank 3.3% (95% CI: 3.0%–3.7%) (Supplementary Table 13).

**Figure 4:**
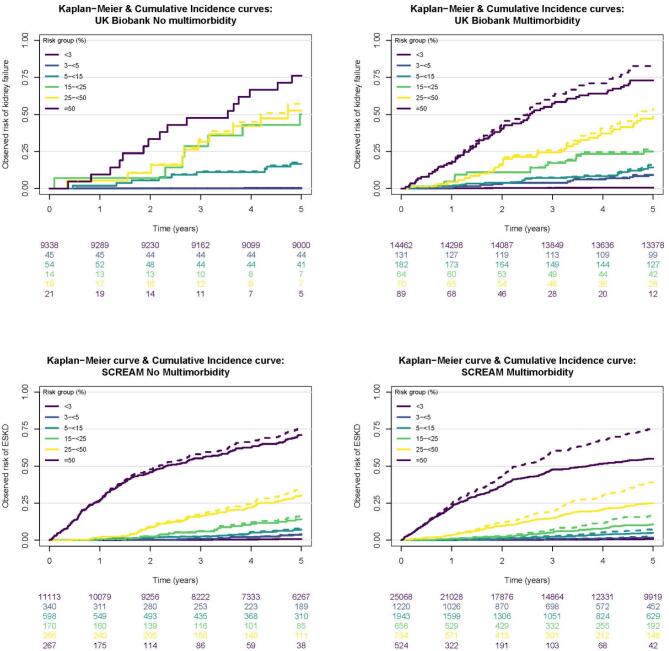
Kaplan–Meier estimates (dashed lines) and Aalen–Johansen estimates (solid lines) cumulative incidence curves for kidney failure by multimorbidity status and KFRE risk groups in UK Biobank and SCREAM.

Plots comparing survival analyses not accounting for competing risk (Kaplan–Meier survival curve) with analyses that do consider competing risk (Aalen–Johansen cumulative incidence curves) showed overestimation of kidney failure risk in the death-censored approach. This was most apparent in SCREAM, the multimorbidity subgroups, and the higher KFRE-predicted risk groups (Fig. 4). Cumulative risk of kidney failure was lower as age advanced but there was an increasing trend in overestimation of kidney failure risk using the death-censored approach for multimorbidity groups as age advanced in SCREAM (Supplementary Fig. 16).

### Updated KFRE model considering competing mortality risk

A model considering competing risk of death (full model details including coefficients, baseline hazards and model terms Supplementary R Data Serialization File 1, Supplementary Table 14) improved performance measures compared to the UK-calibrated 5-year KFRE in multimorbidity groups (Table 5, Supplementary Figs 17 and 18, and Supplementary Text 4), O/E ratio 0.98 in multimorbidity groups of both cohorts at 5 years. Improvements in calibration accuracy were predominantly in the multimorbidity groups. Overall fit of the competing risk model was also improved (increased scaled Brier scores) across both cohorts and multimorbidity groupings. Number of individuals, characteristics, event numbers, and performance for each risk group for each cohort are reported in Supplementary Tables 15 and 16 (KFRE model) and Supplementary Tables 17 and 18 (updated KFRE accounting for competing mortality risk). Total referral numbers increased in both cohorts when a threshold of kidney failure risk >5% was applied using the competing risk model compared to KFRE. Individuals referred had lower levels of multimorbidity, fewer death events by 5 years and in the SCREAM cohort were younger (Supplementary Table 19). The number of kidney failure events within 5 years was greater when the competing risk model was used compared to KFRE.

**Table 5:** Performance measures for validation of the competing mortality risk model using age, sex, log uACR, and eGFRcr to predict kidney failure at 5 years in UK Biobank and SCREAM subgroups.

Performance measure for competing risk model at 5-years					
	Whole cohort	Multimorbidity		No multimorbidity	
	UK Biobank	UK Biobank	SCREAM	UK Biobank	SCREAM
Discrimination					
Time dependent AUC	0.92 (0.90–0.94)	0.92 (0.89–0.93)	0.93 (0.91–0.94)	0.94 (0.91–0.97)	0.95 (0.93–0.96)
C-index	0.92 (0.90–0.93)	0.91 (0.89–0.93)	0.92 (0.90–0.93)	0.93 (0.91–0.96)	0.94 (0.93–0.96)
Calibration					
O/E ratio	0.93 (0.83–1.03)	0.98 (0.86–1.11)	0.98 (0.91–1.06)	0.79 (0.62–0.99)	0.83 (0.75–0.92)
Calibration intercept	−0.17 (−0.32–−0.02)	−0.17 (−0.33–0.00)	0.17 (0.08–0.27)	−0.17 (−0.48–0.14)	0.10 (−0.04–0.24)
Calibration slope	0.96 (0.87–1.06)	0.93 (0.83–1.04)	1.12 (1.03–1.21)	1.05 (0.86–1.25)	1.01 (0.90–1.11)
Overall fit					
Brier score	0.010 (0.008–0.010)	0.011 (0.010–0.013)	0.021 (0.019–0.022)	0.005 (0.004–0.006)	0.021 (0.019–0.024)
Scaled brier score (%)	28.25 (23.44–33.09)	27.61 (21.82–33.75)	27.98 (25.28–30.73)	29.99 (19.17–39.43)	37.54 (32.89–41.97)

Sensitivity analyses are presented in Supplementary Text 5, Supplementary Tables 20 and 25, and Supplementary Figs 19 and 20.

## DISCUSSION

In a research-based cohort of 24 489 individuals and population-based cohort of 42 902, 61.2% and 70.3% individuals, respectively, had multimorbidity. This study found that: (i) The four-variable KFRE has good discrimination but suboptimal calibration for CKD patients with and without multimorbidity. (ii) There was no improvement in KFRE model performance when using eGFRcys or eGFRcr-cys compared to eGFRcr. (iii) A competing risk model developed, using the same variables as KFRE, performs better for all individuals with CKD, but particularly among people with multimorbidity. The competing risk model categorizes more individuals as having >5% risk of kidney failure but more of these develop kidney failure and fewer die within 5 years.

Comparisons of characteristics of our study population and the original cohorts used to develop KFRE show that our cohorts had a higher baseline eGFR, lower levels of albuminuria, a higher proportion of females, and lower kidney failure events, and the UK Biobank cohort was younger [5]. However, the SCREAM cohort had a similar prevalence of cardiometabolic conditions as the KFRE development cohort [5].

The importance of external validation of a prognostic model across different settings, populations, and subgroups due to variation in case mix and the impact that this can have on prognostic model performance has been highlighted [47]. KFRE [36] has been validated in 31 multinational cohorts, including >30 countries. In keeping with our findings, KFRE has good discrimination across all cohorts and additionally across subgroups of age, race, and diabetes. However, KFRE over-estimates risk in some non-North American cohorts, requiring model recalibration. Validation in a UK cohort required recalibration, particularly in lower risk groups [35]. Other studies highlight differences in model performance by ethnicity [48] and renal disease aetiologies [49].

Using cystatin C based eGFR did not impact performance of KFRE. Existing evidence relating to cystatin C suggests eGFRcys can improve risk stratification and discrimination of future kidney failure [50], cardiovascular disease and mortality [17, 27]. Although others have found no benefits of KFRE using eGFRcys in mild to moderate CKD [51]. We found that individuals with multimorbidity had similar eGFRcr but lower mean eGFRcys compared to those without multimorbidity. There may be subgroups of individuals with multimorbidity with discordant eGFRcr and eGFRcys, warranting exploration of the impact of this discrepancy on KFRE model performance and which subgroups may benefit from the use of cystatin C testing.

Albuminuria and eGFR are the covariates with greatest contribution to predicting risk of kidney failure in KFRE [5]. Subgroups with multimorbidity in both cohorts had higher levels of albuminuria, which may underpin the lesser impact of eGFR formulae compared to albuminuria. It is unclear whether albuminuria seen in multimorbidity extends beyond simply being associated with diabetes, which is a major component of multimorbidity. More accurate identification of high-risk individuals and, at an earlier time point, is imperative: particularly in an era where therapies such as sodium-glucose cotransporter-2 inhibitors offer cardiovascular and renal benefits. These subgroups may stand to gain the most from early and tailored interventions, but consideration should also be given to the potential increased risk of adverse events or side effects that they may experience.

A lack of methodological guidance for competing risk analyses has probably contributed to a lack of use of these methods [44]. Competing mortality risk affects prognostic performance measures in other models in older adults and in those with multimorbidity [13, 44]. There have been mixed findings from studies accounting for competing risk of mortality in KFRE [5, 48, 52, 53]. Some studies accounting for competing mortality risk in KFRE highlight improvements in calibration in subgroups [48, 52]. Grams *et al.* found an improvement in calibration at 5 years when accounting for competing risk of death among adults ≥65 years old and in those with eGFR between 45 and 59 ml/min/1.73 m^2^ [52]. Ramspek *et al.* demonstrated models making predictions over longer time periods overestimate risk of kidney failure, specifically finding the 5-year four-variable KFRE overpredicted kidney failure risk by 10% when not accounting for competing mortality [11]. Likewise, Al-Wahsh *et al.* directly compared the prognostic performance of a standard Cox regression, cause-specific Cox regression, and Fine–Gray models. They showed that, in individuals with more advanced CKD (stage 4), miscalibration was particularly evident in older adults and those with more comorbidity (diabetes or cardiovascular disease) when longer prediction horizons were used [53].

We highlight the high risk of mortality in individuals with CKD especially in those with multimorbidity. We demonstrate improvements in calibration and overall fit when a model, using the same covariates, accounts for competing mortality risk, predominantly in multimorbidity subgroups. This is important for all individuals with CKD, although will have a greater impact on those at the highest mortality risk, e.g. those with multimorbidity [10], and when predicting risk over longer follow up [53].

Some studies compare concurrent use of models assessing both kidney failure and mortality risk [54] or a model predicting both simultaneously (KD-predict) [55]. Such models are useful in understanding risk of death and kidney failure in relation to each other. It is uncertain whether this approach helps communications between healthcare professionals and patients.

There are other validated prognostic models that may be used in clinical practice, e.g. the CKD-PC ‘risks’ model [56] and KD-predict [55], both of which account for or consider risk of mortality. Comparison and clinical utility studies are required to inform the best model for use in clinical practice.

Our study has several strengths. The combined datasets included >63 000 individuals with >10 000 kidney failure events, resulting in a large and precise validation study. We include cohorts from research and population-based cohorts to strengthen findings across different populations and settings [57]. This is the first study that we are aware of validating KFRE in those with multiple LTCs and that also considers the competing risk of mortality. We have also followed robust methodologies [44, 46].

We acknowledge some limitations. The cohorts may not be representative of populations outside Northern Europe. UK Biobank is a high-quality research database, but consists of fewer socio-economically deprived individuals, however, there is evidence that risk factor associations are generalizable [58]. There was selection bias in SCREAM as participants missing baseline albuminuria data were excluded. Some groups, such as those with diabetes or hypertension, are more likely to have albuminuria tested. We reduced this bias by converting dipstick proteinuria results in the SCREAM cohort (reflecting individuals having proteinuria tested as ‘screening’) and by including UK Biobank, where albuminuria was universally measured. Around 1.6 million individuals were excluded due to missing cystatin C measurements also contributing selection bias, as individuals may be more likely to have cystatin C measured when clinical characteristics—such as extremes of muscle mass, frailty, increased comorbidity, or older age [59]—may result in inaccuracies in creatinine-based eGFR. There were also differences in the measurements of LTCs between UK Biobank and SCREAM. Small kidney failure event numbers in some multimorbidity cluster groups (complex and mixed mental/physical multimorbidity in UK Biobank) limited our ability to conduct external validation. The kidney failure outcome definition we used did not include conservative (non-KRT) kidney care which may be more common in individuals with multimorbidity and advancing age. Finally, we observed violation of PH assumptions in competing risk models (notably for eGFR and age), such violations may result in misinterpretation of time-specific hazards associations and bias interpretation of constant hazard ratios if effects vary meaningfully over time [60, 61]. Our primary aim, however, was absolute risk estimation at a fixed 5-year horizon and comparability with the four-variable KFRE, contexts in which modest departures are less consequential for predicted risks.

## CONCLUSIONS

KFRE has good discrimination accuracy to predict kidney failure in individuals with multimorbidity but has less robust calibration and crucially does not account for competing risk of death. A model accounting for competing mortality risk improves model performance, particularly calibration, in individuals with multimorbidity.

## DATA AVAILABILITY STATEMENT

Data used for this study are available from UK Biobank and SCREAM. Access to data is through data application processes for each data source and fulfilment of ethics and GDPR regulations. Further details can be found via https://www.ukbiobank.ac.uk/use-our-data/apply-for-access/ for UK Biobank and by contacting juan.jesus.carrero@ki.se for SCREAM. Analysis of UK Biobank was conducted as part of project 14151. Code is available on GitHub for others to replicate our analysis: https://github.com/h-j-walker/KFRE_validation.git.

## Supplementary Material

gfaf252_Supplemental_File
