# Supplementary material for: The kidney failure risk equation in people with CKD and multimorbidity: the effect of competing mortality risks
Source: Nephrol Dial Transplant. 2025 Nov 25;41(7):1239–52. doi: 10.1093/ndt/gfaf252 (PMC13403277; doi:10.1093/ndt/gfaf252)
Supplement: gfaf252_Supplemental_File [file gfaf252_Supplemental_File.docx]

All UK Biobank participants

n = 502,503

Excluded participants:

1. Missing creatinine measurement (n=33,133)
2. Missing uACR measurement (n=13,402)
3. Missing cystatin C measurement (n=333)
4. eGFR >60ml/min/1.73m^2^ (n=430,944)
5. KRT before recruitment (n=52)

UK Biobank participants included in analysis

n = 24,489

Supplementary Figure 1. Flow diagram of participant selection and exclusions UK Biobank cohort

SCREAM participants with creatinine measurement

n = 1,802,601

Excluded participants:

1. Missing same day cystatin C measurement

(n=1,599,698)

1. Missing albuminuria measurement within 12 months

(n=114,909)

1. KRT before recruitment

(n=1,082)

1. GFR >60ml/min/1.73m^2^

(n=44,010)

SCREAM participants included in analysis

n = 42,902

Supplementary Figure 2. Flow diagram of participant selection and exclusions SCREAM cohort


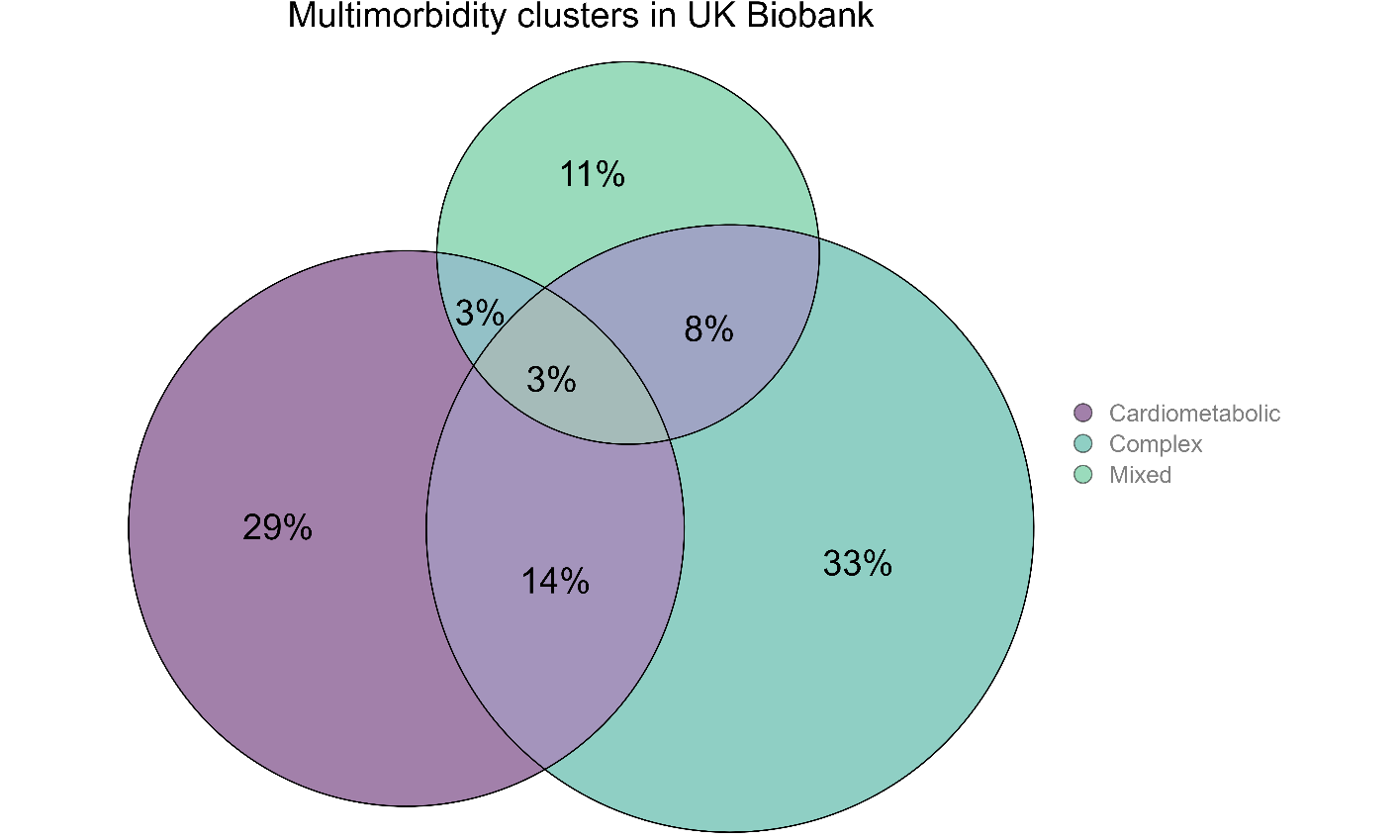

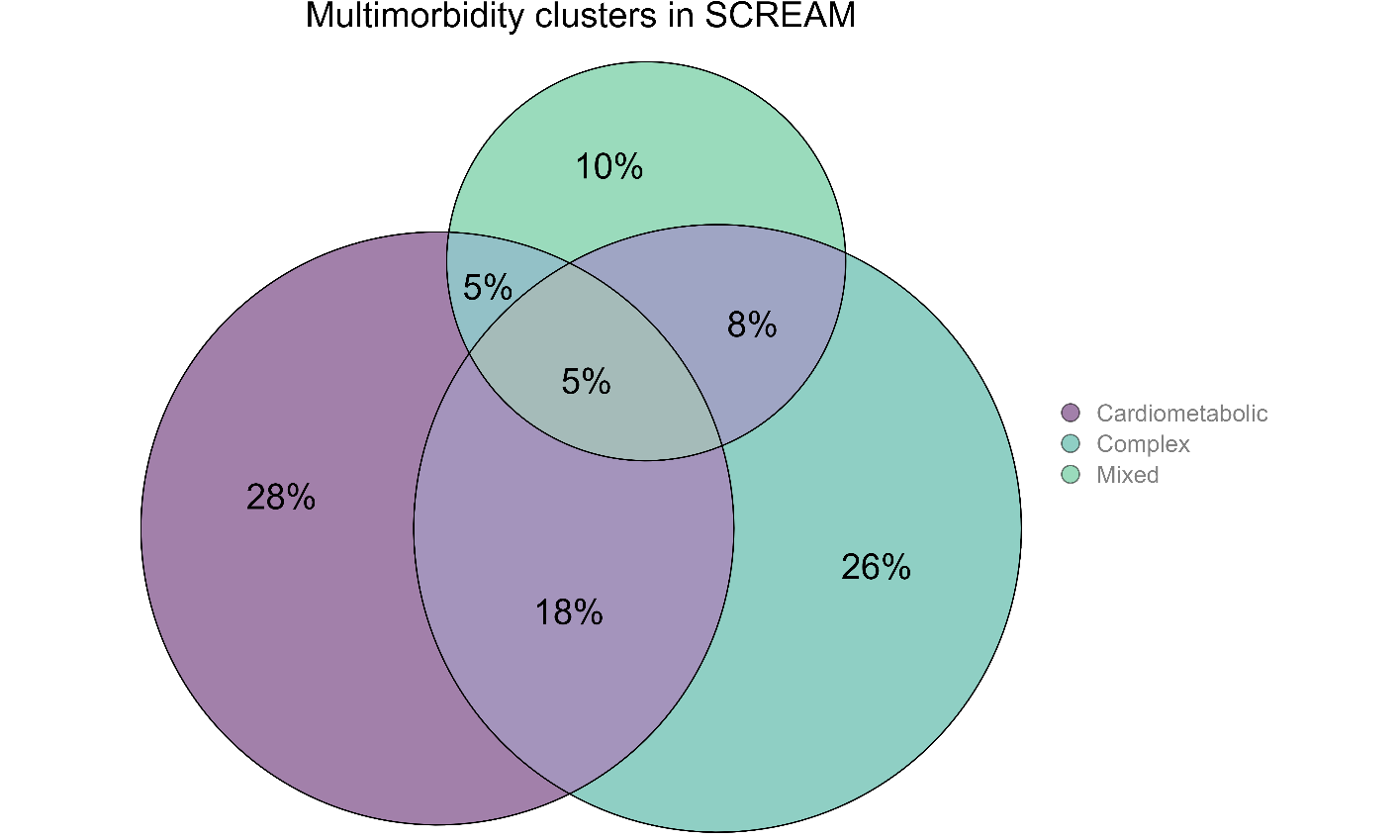


Supplementary Figure 3. Euler diagrams showing overlap between multimorbidity clusters in UK Biobank and SCREAM cohorts.


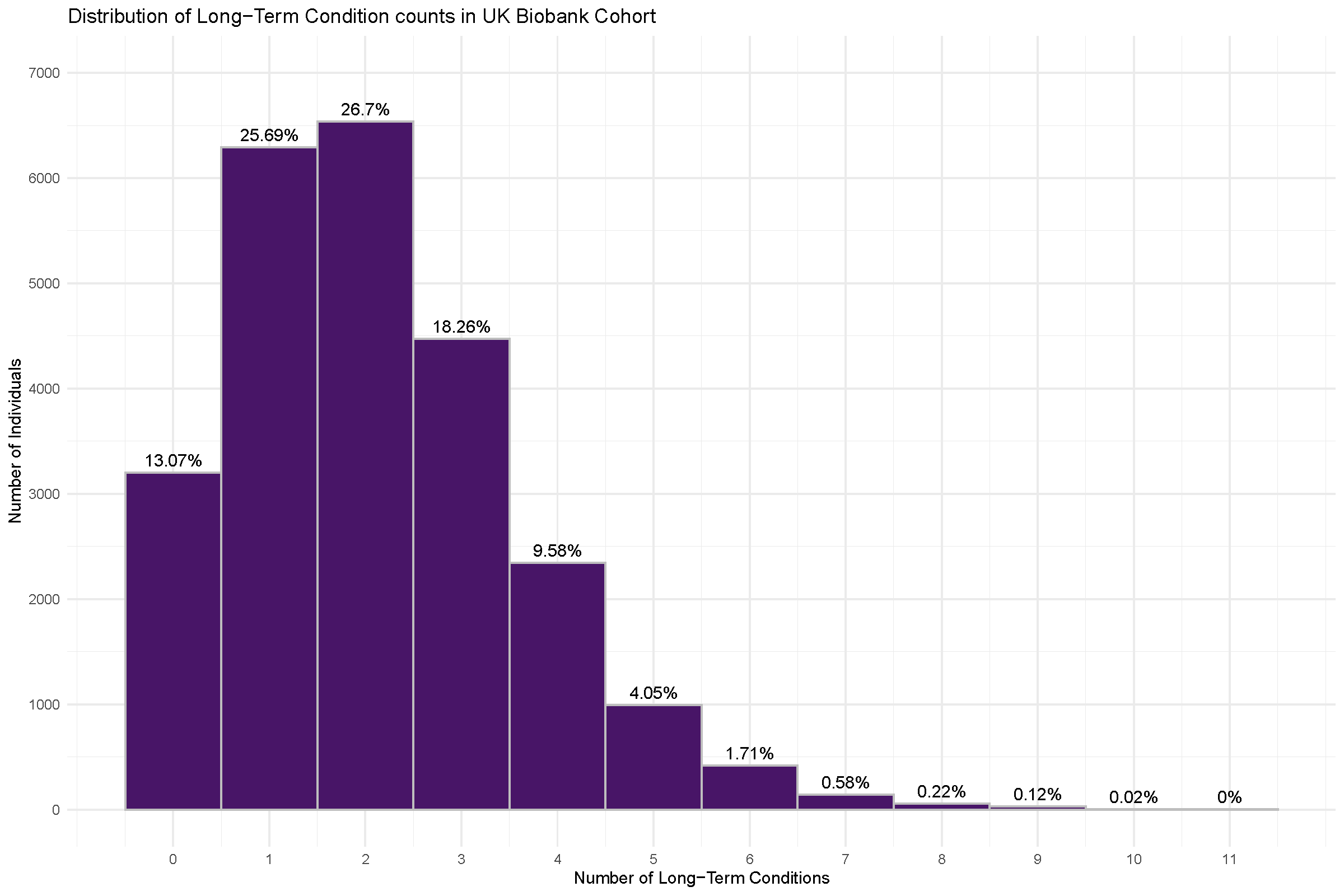


Supplementary Figure 4. Distribution of long-term condition counts: UK Biobank


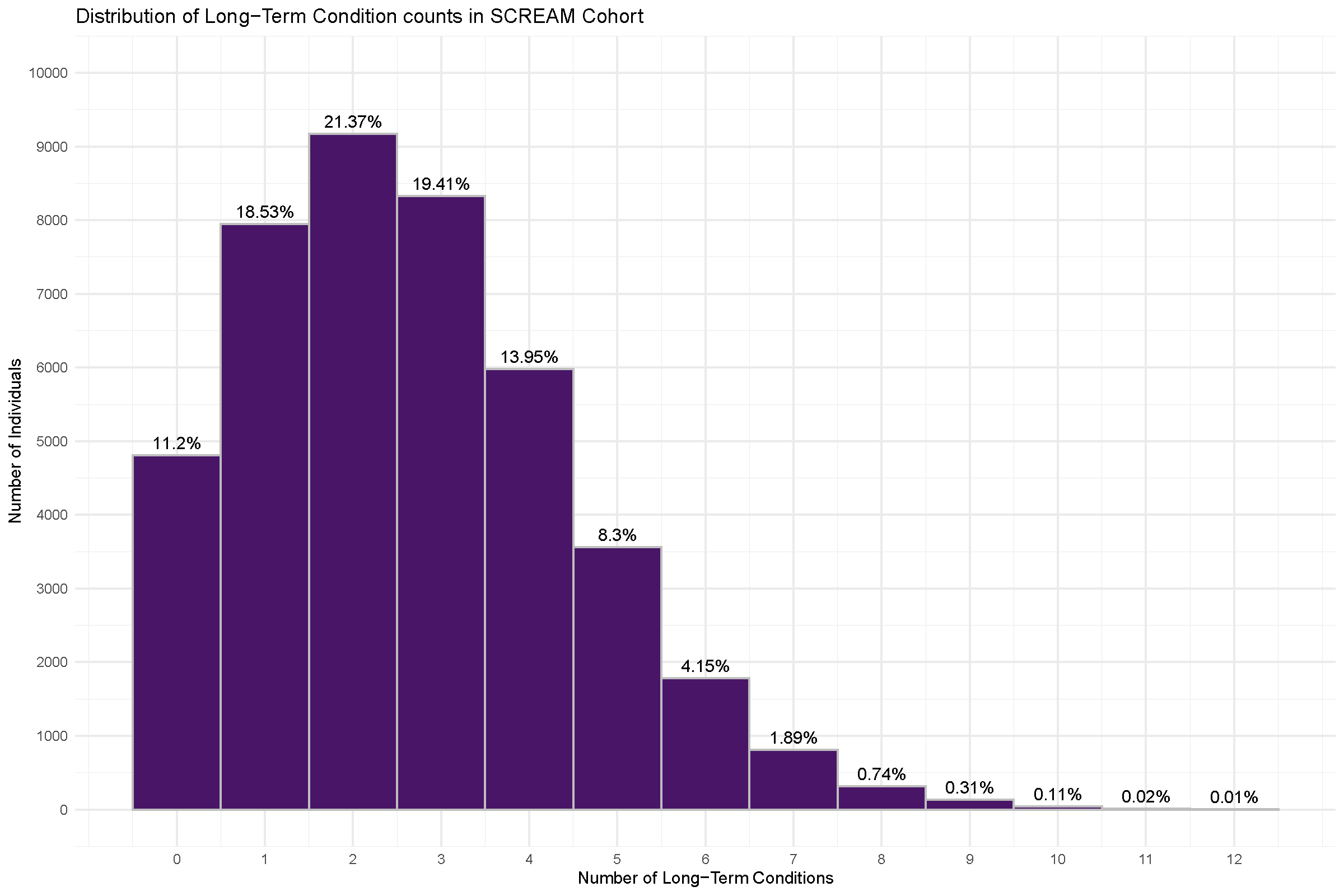


Supplementary Figure 5. Distribution of long-term condition counts: SCREAM


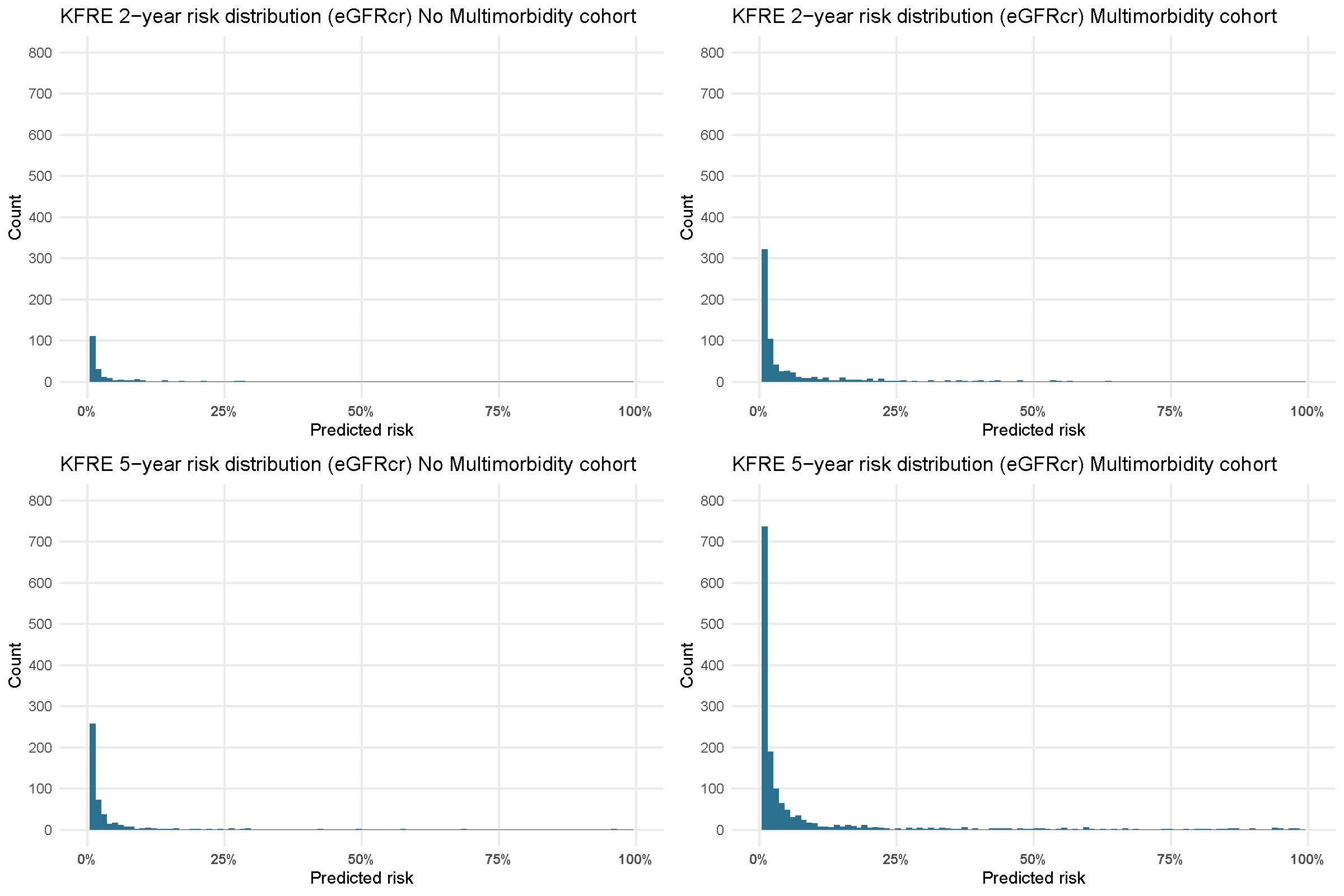


Supplementary Figure 6. Histograms showing distribution of the predicted 2-year and 5-year risk of kidney failure by multimorbidity status in UK Biobank cohort. Predicted risk calculated by the UK calibrated KFRE using eGFRcr.


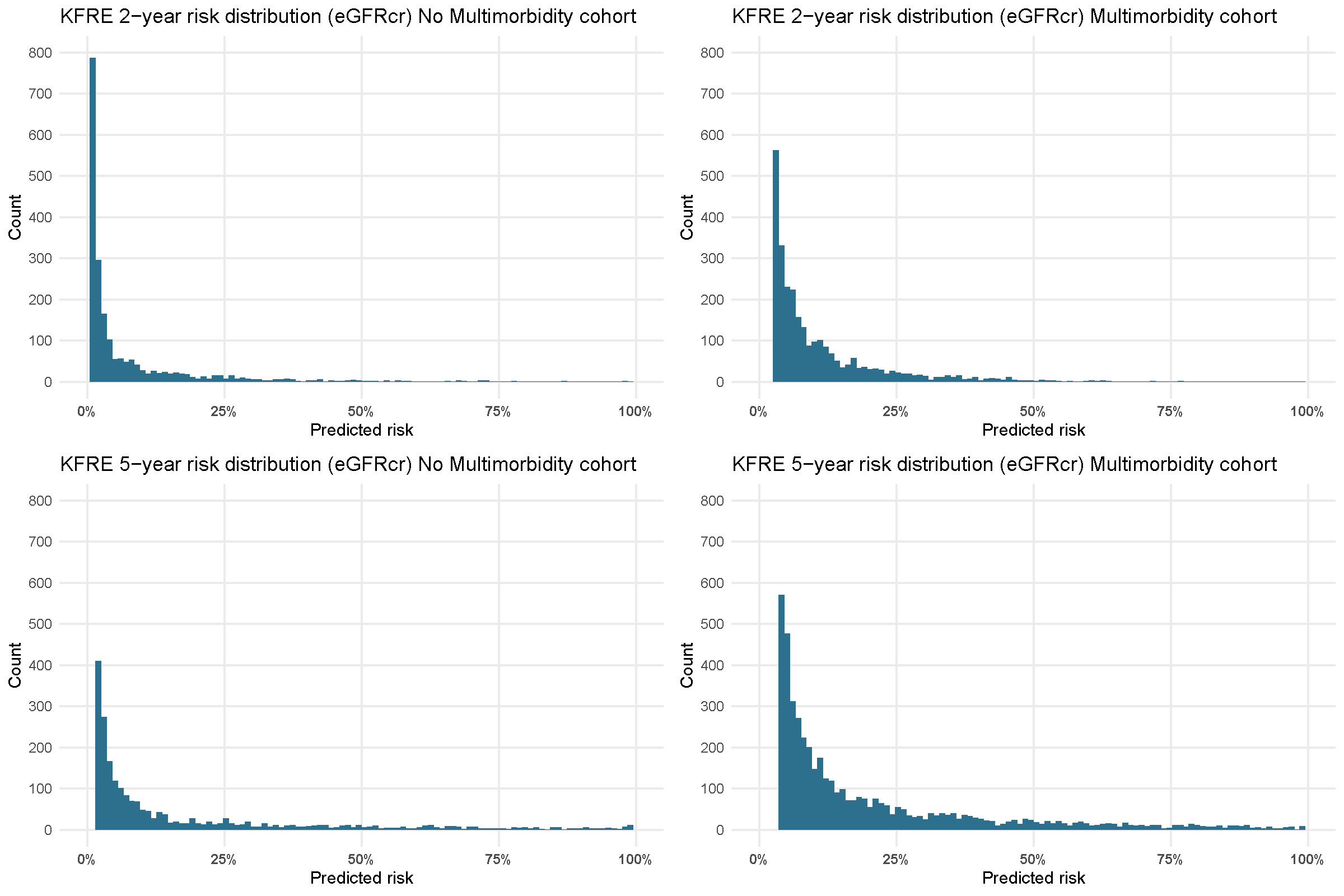


Supplementary Figure 7. Histograms showing distribution of the predicted 2-year and 5-year risk of kidney failure by multimorbidity status in SCREAM cohort. Predicted risk calculated by the UK calibrated KFRE using eGFRcr.


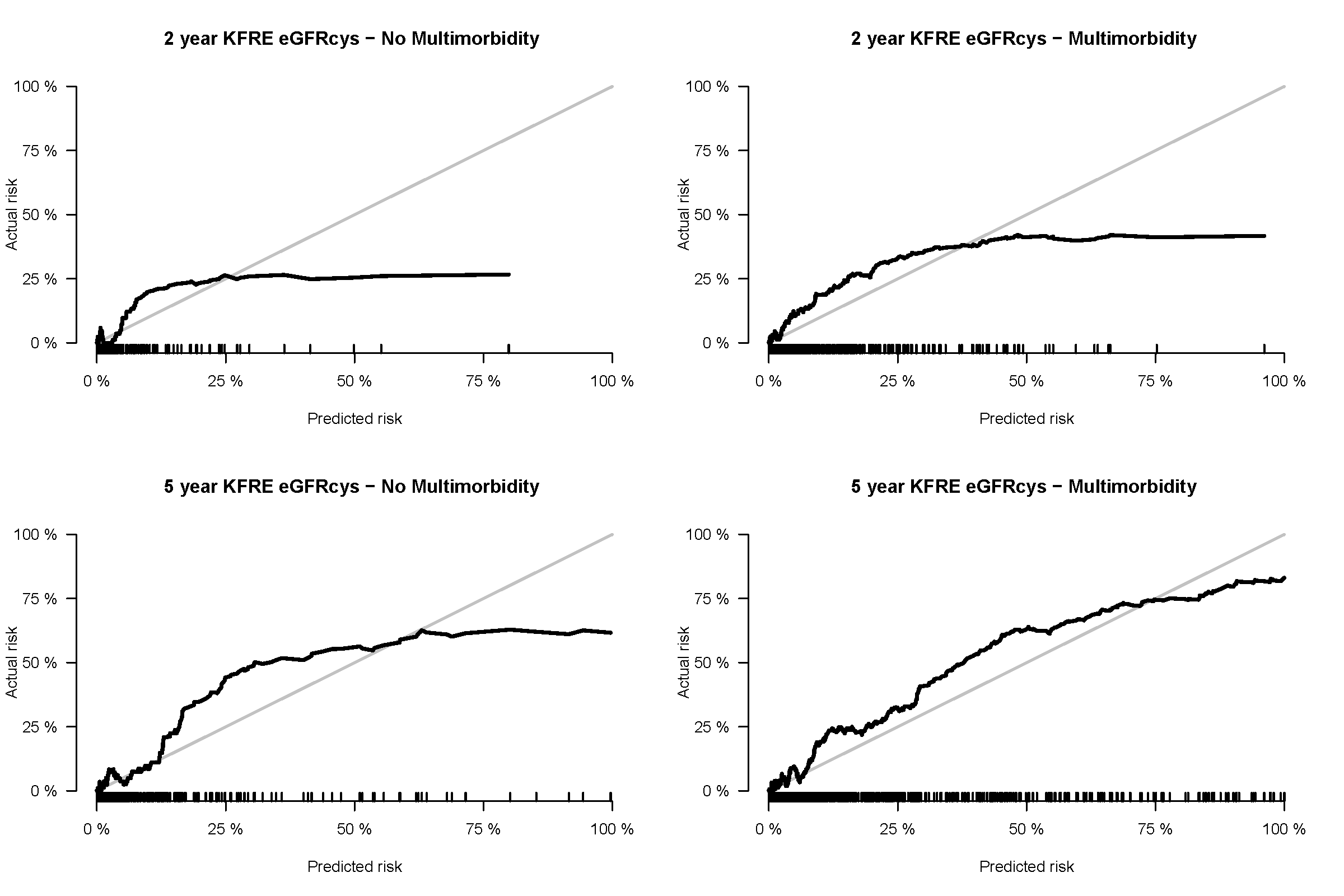


Supplementary Figure 8. Calibration curves for predicted versus observed 2- and 5-year risk of kidney failure by multimorbidity status in UK Biobank cohort. Predicted risk is according to the UK calibrated KFRE using **eGFRcys**.


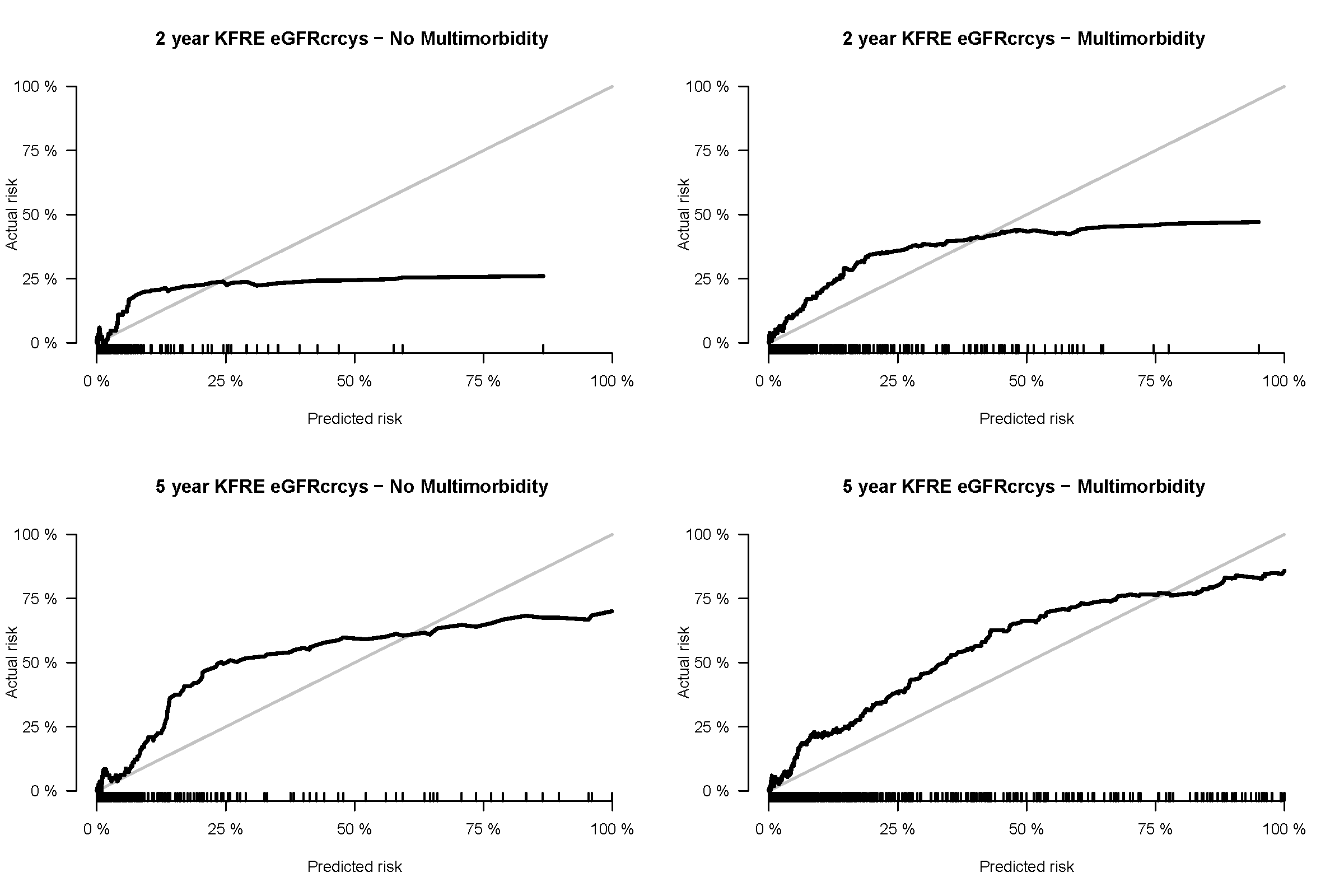


Supplementary Figure 9. Calibration curves for predicted versus observed 2- and 5-year risk of kidney failure by multimorbidity status in UK Biobank cohort. Predicted risk is according to the UK calibrated KFRE using **eGFRcrcys**.


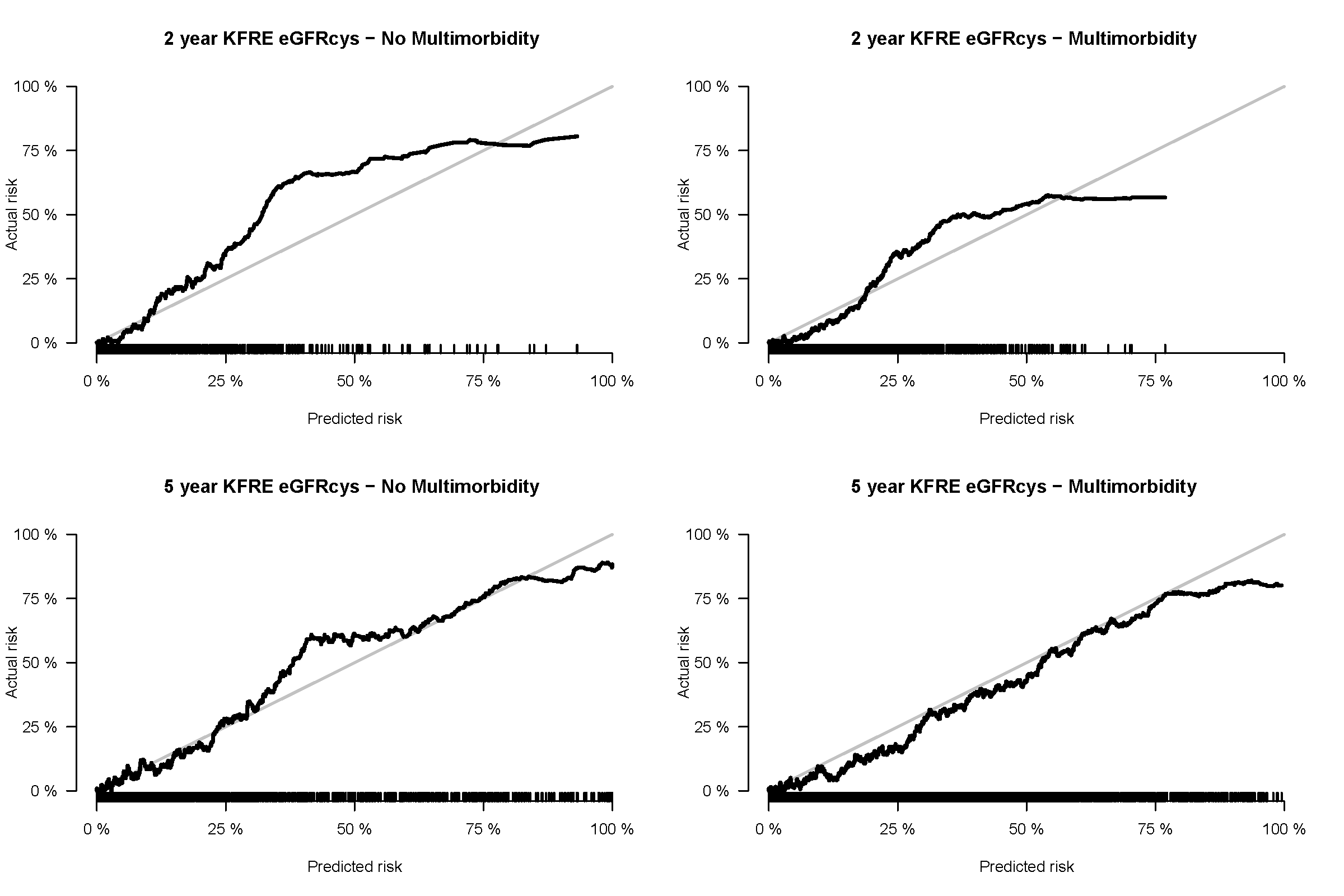


Supplementary Figure 10. Calibration curves for predicted versus observed 2- and 5-year risk of kidney failure by multimorbidity status in SCREAM cohort. Predicted risk is according to the UK calibrated KFRE using **eGFRcys**.


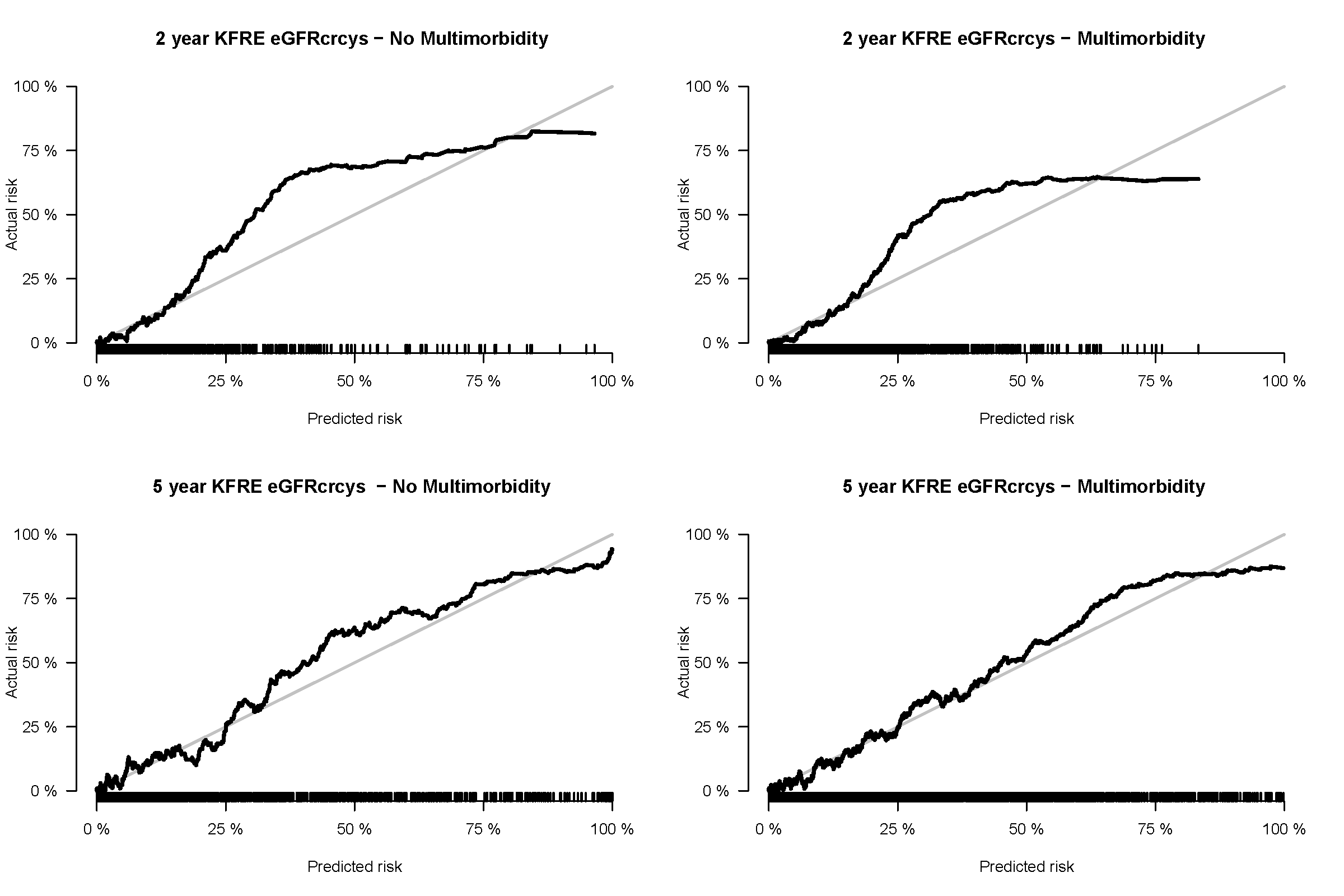


Supplementary Figure 11. Calibration curves for predicted versus observed 2- and 5-year risk of kidney failure by multimorbidity status in SCREAM cohort. Predicted risk is according to the UK calibrated KFRE using **eGFRcrcys**.


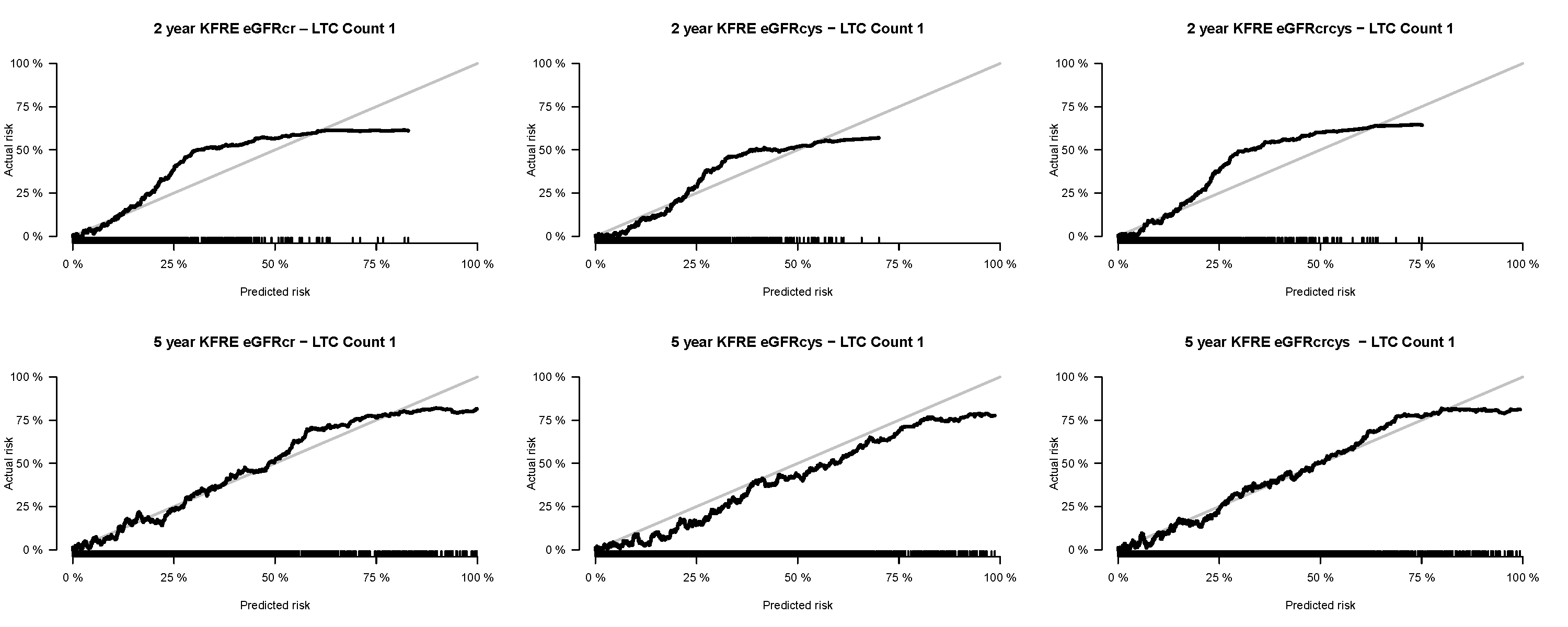

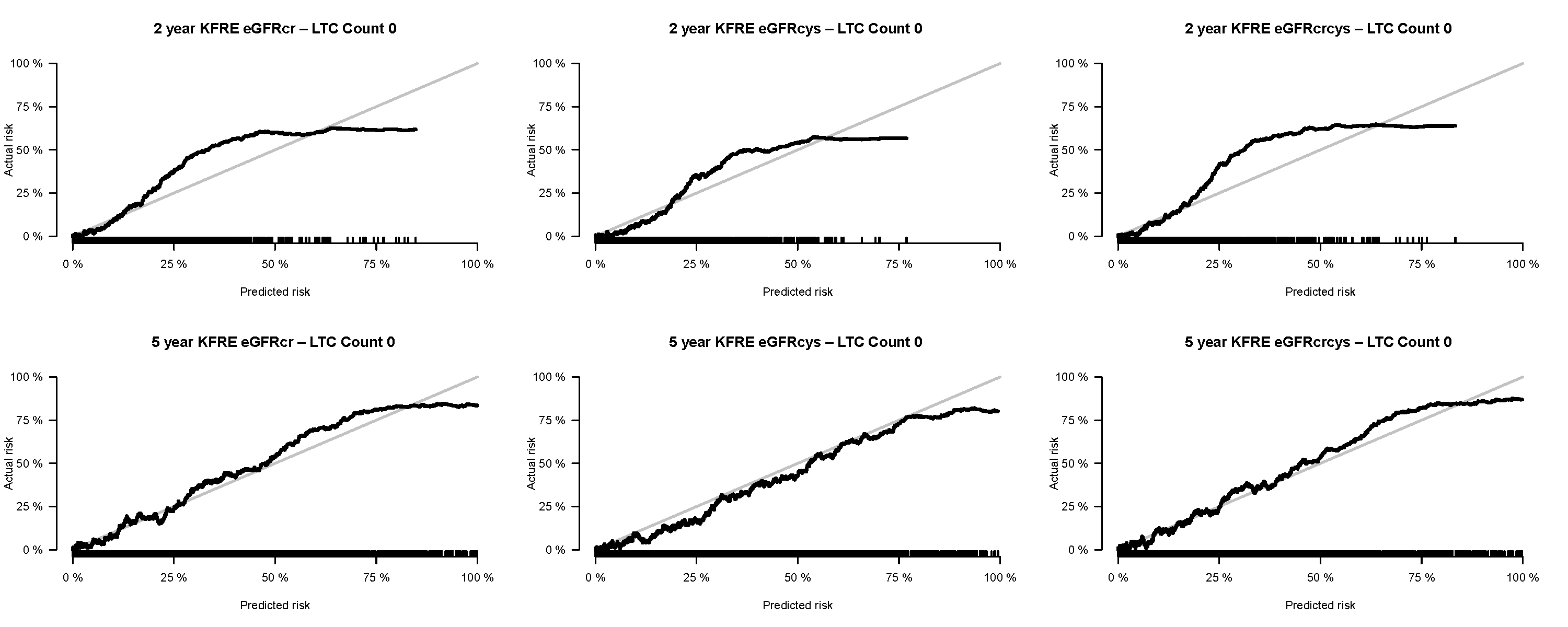


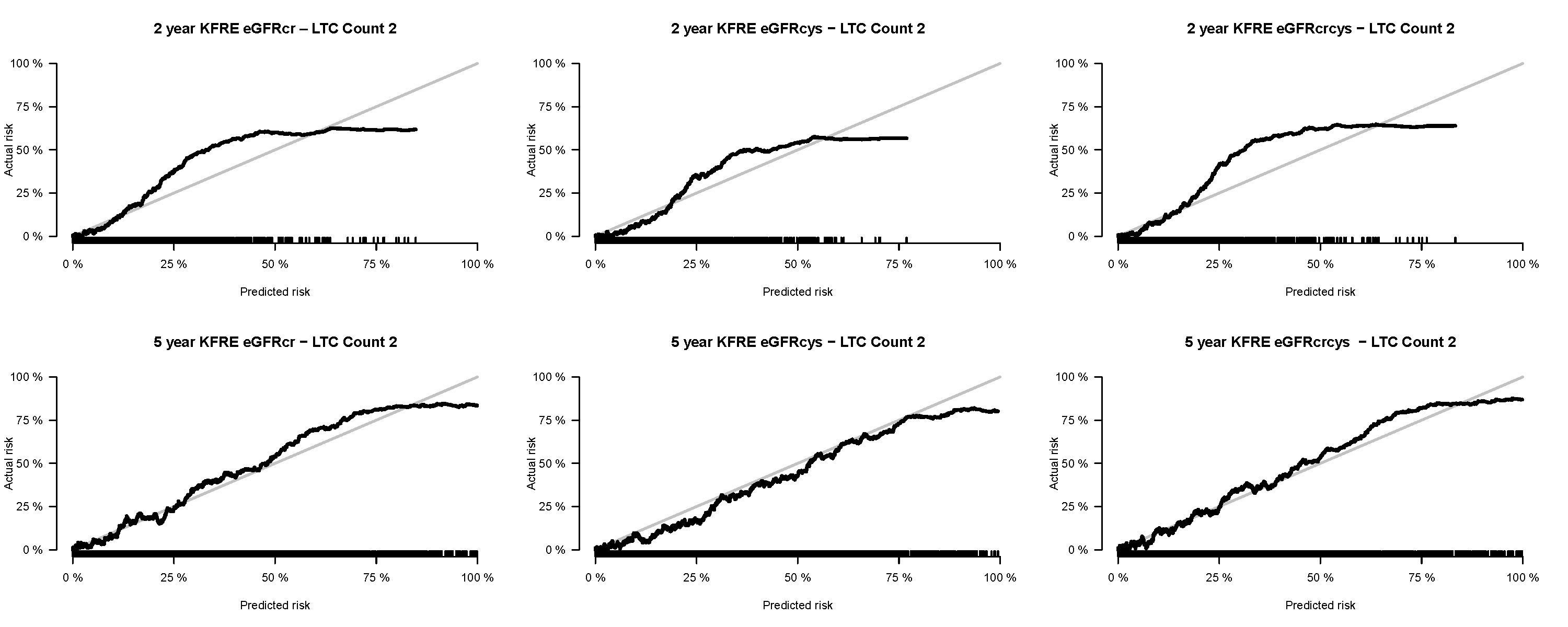


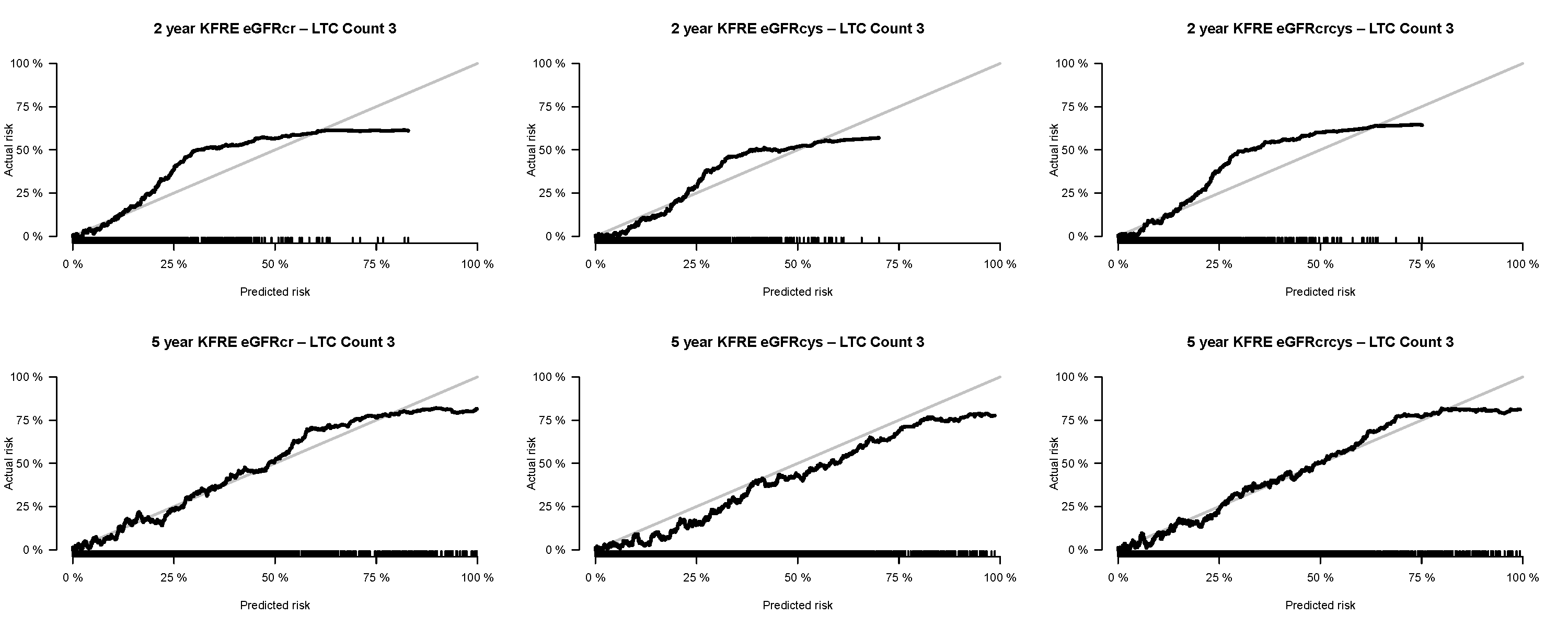


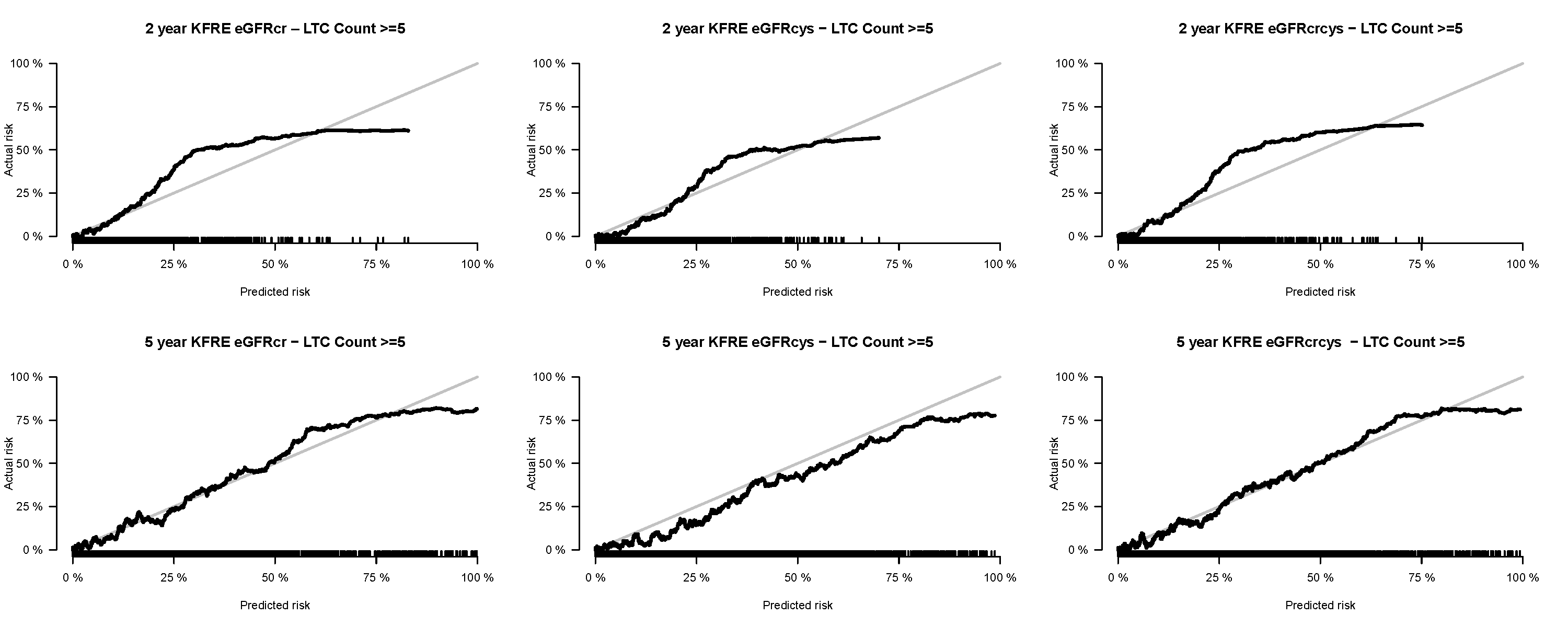

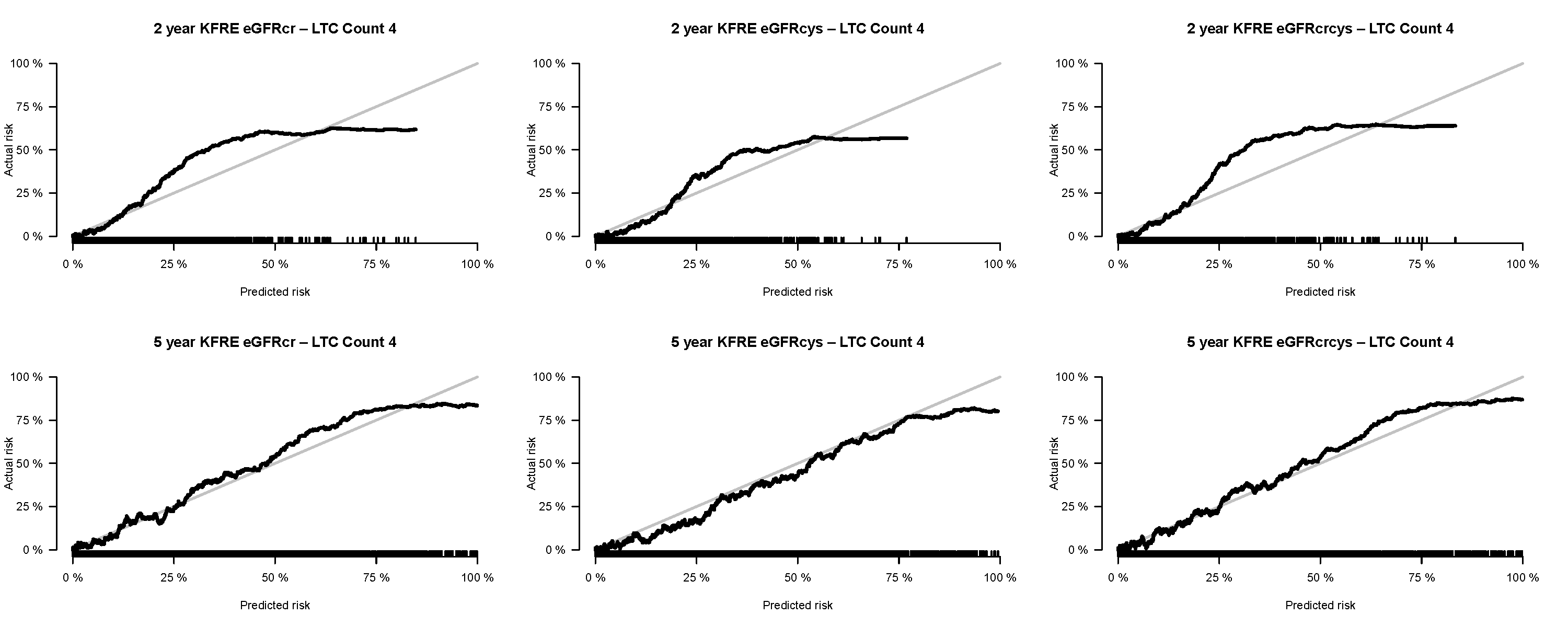


Supplementary Figure 12. Calibration curves for predicted versus observed 2- and 5-year risk of kidney failure by LTC count and different eGFR equations used in KFRE for SCREAM cohort. Predicted risk is according the UK calibrated KFRE.


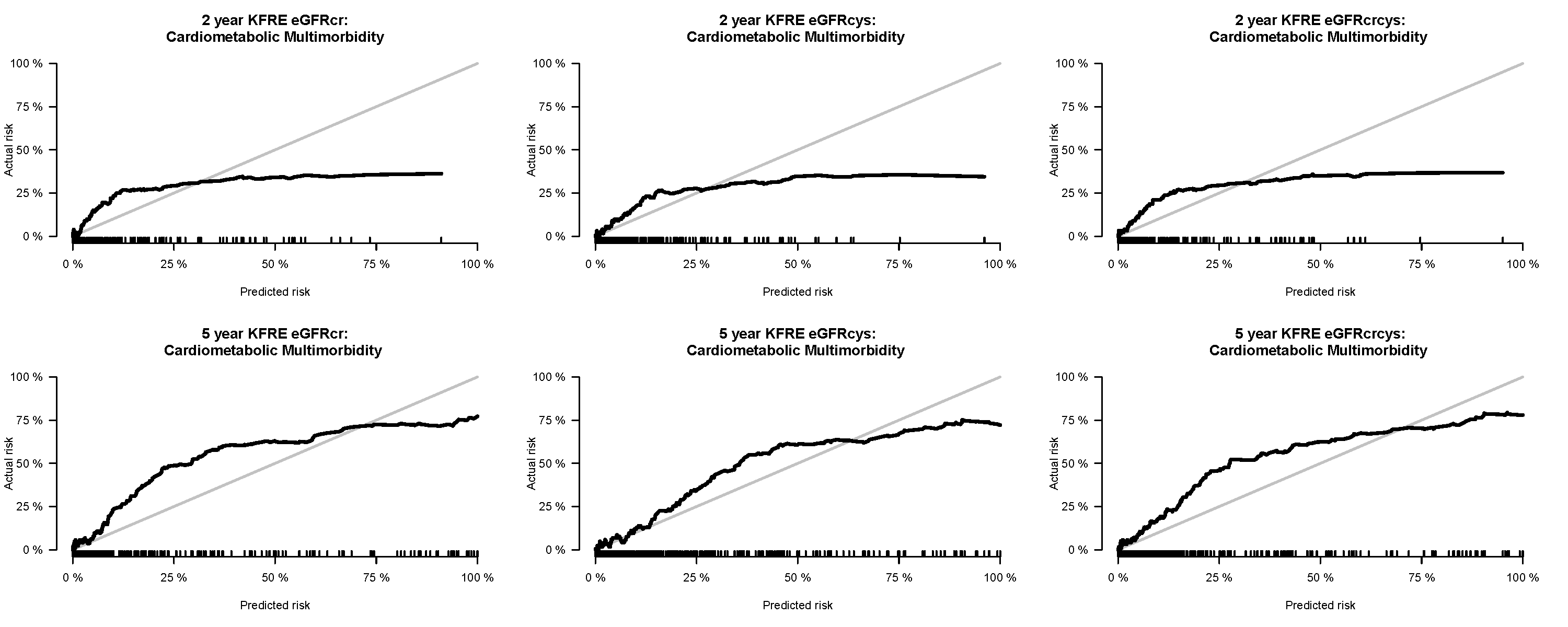


Supplementary Figure 13. Calibration curves for predicted versus observed 2- and 5-year risk of kidney failure cardiometabolic multimorbidity and different eGFR equations used in KFRE for UK Biobank cohort. Predicted risk is according to the UK calibrated KFRE.


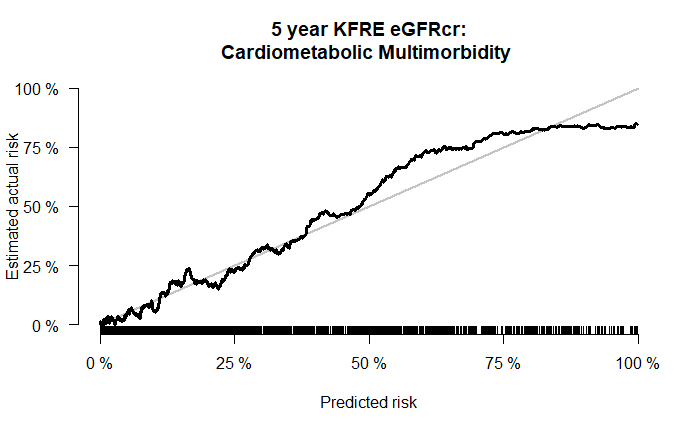

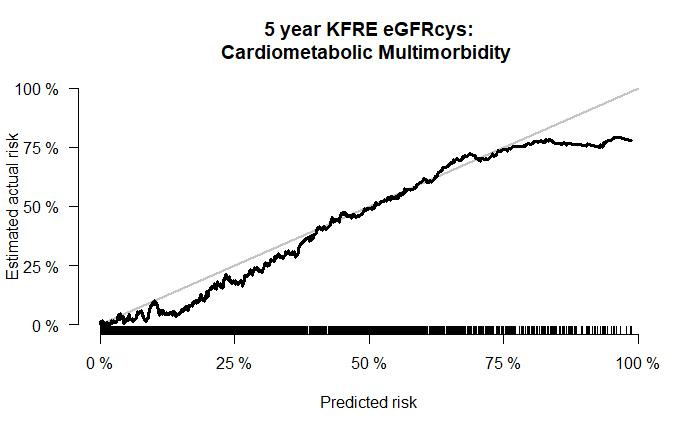


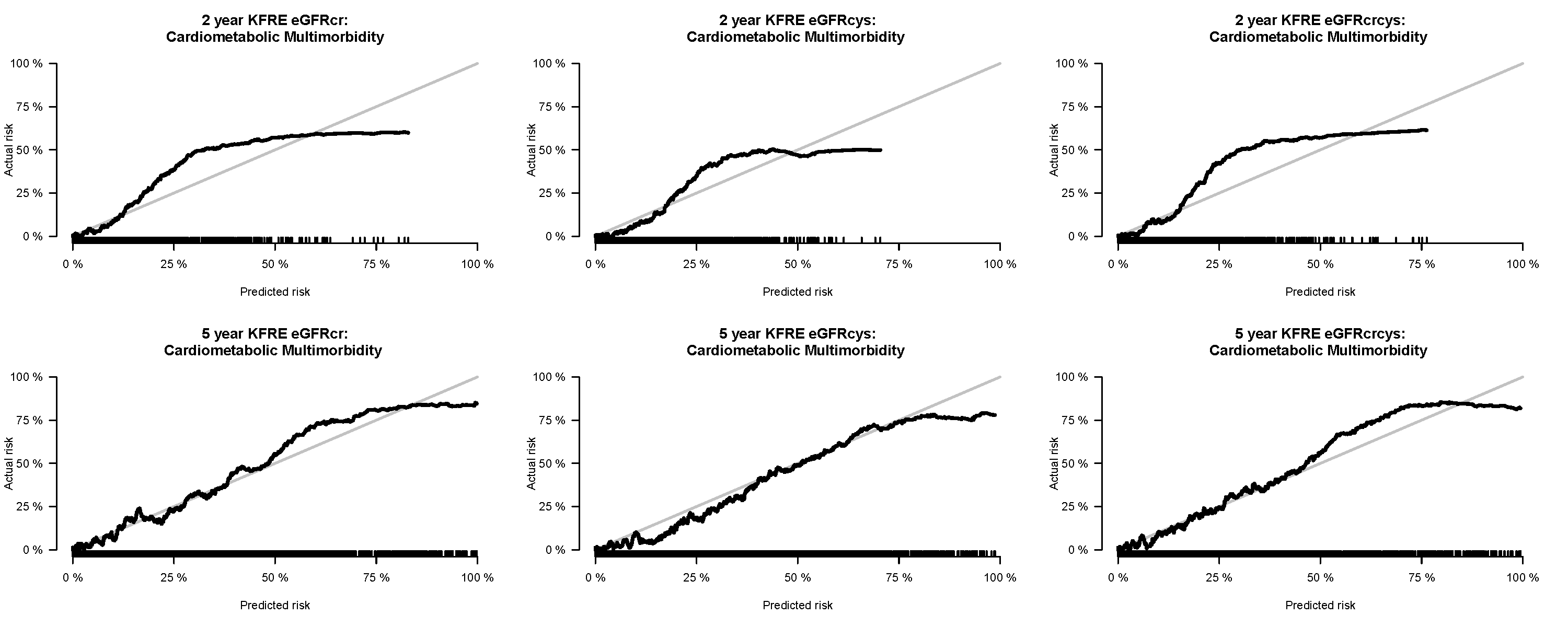


Supplementary Figure 14. Calibration curves for predicted versus observed 2- and 5-year risk of kidney failure cardiometabolic multimorbidity and different eGFR equations used in KFRE for SCREAM cohort. Predicted risk is according to the UK calibrated KFRE.


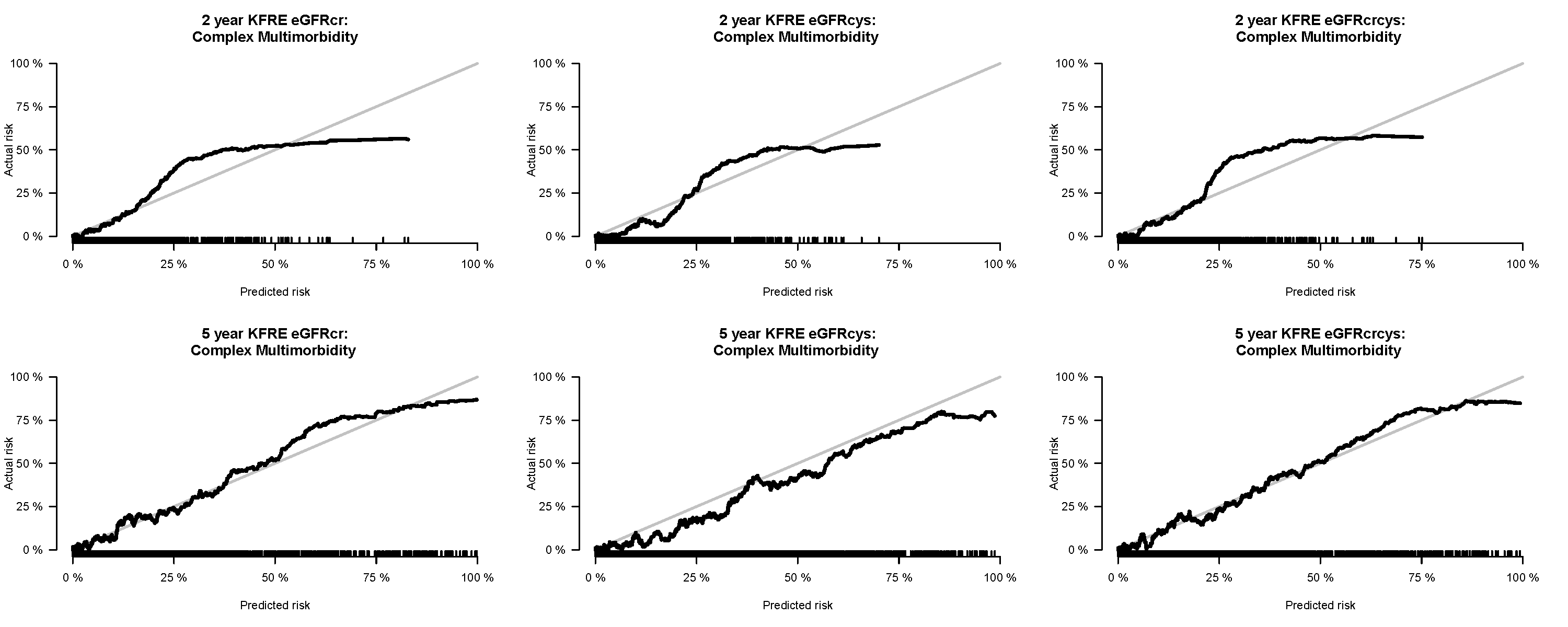


Supplementary Figure 15. Calibration curves for predicted versus observed 2- and 5-year risk of kidney failure complex multimorbidity and different eGFR equations used in KFRE for SCREAM cohort. Predicted risk is according to the UK calibrated KFRE.


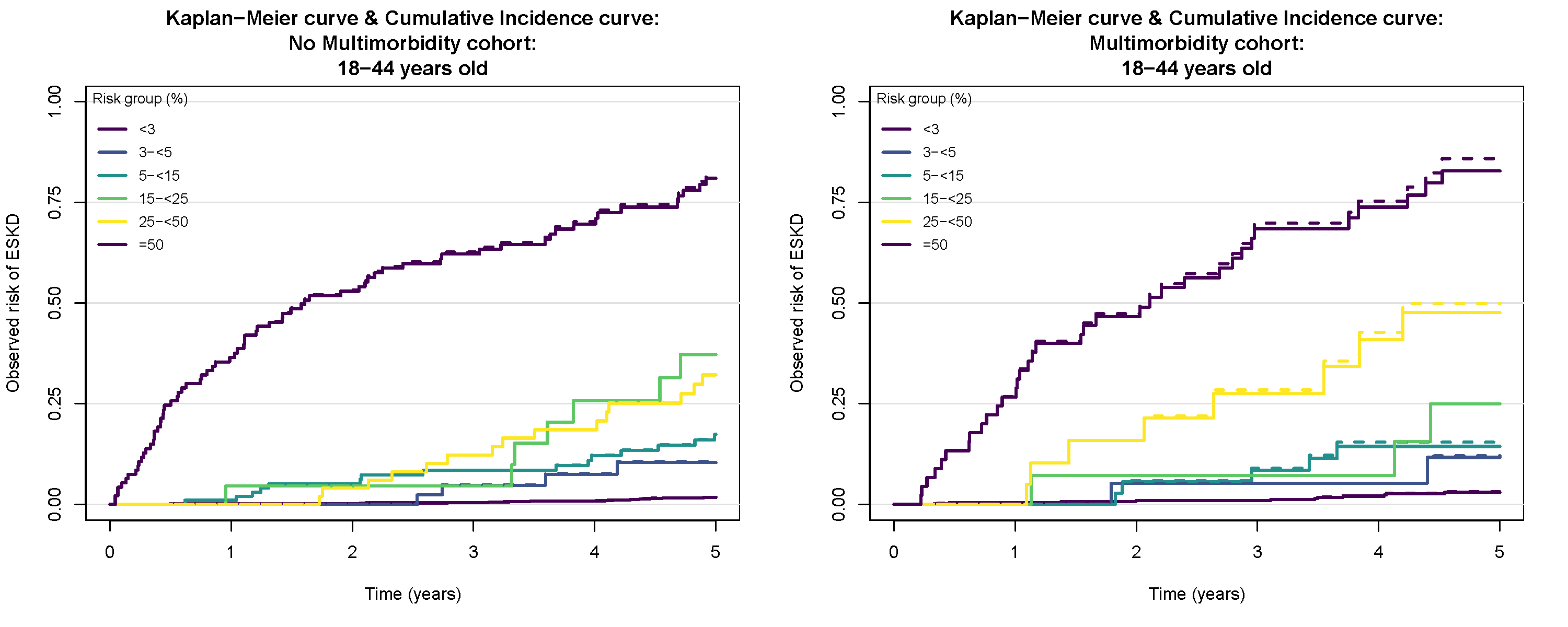


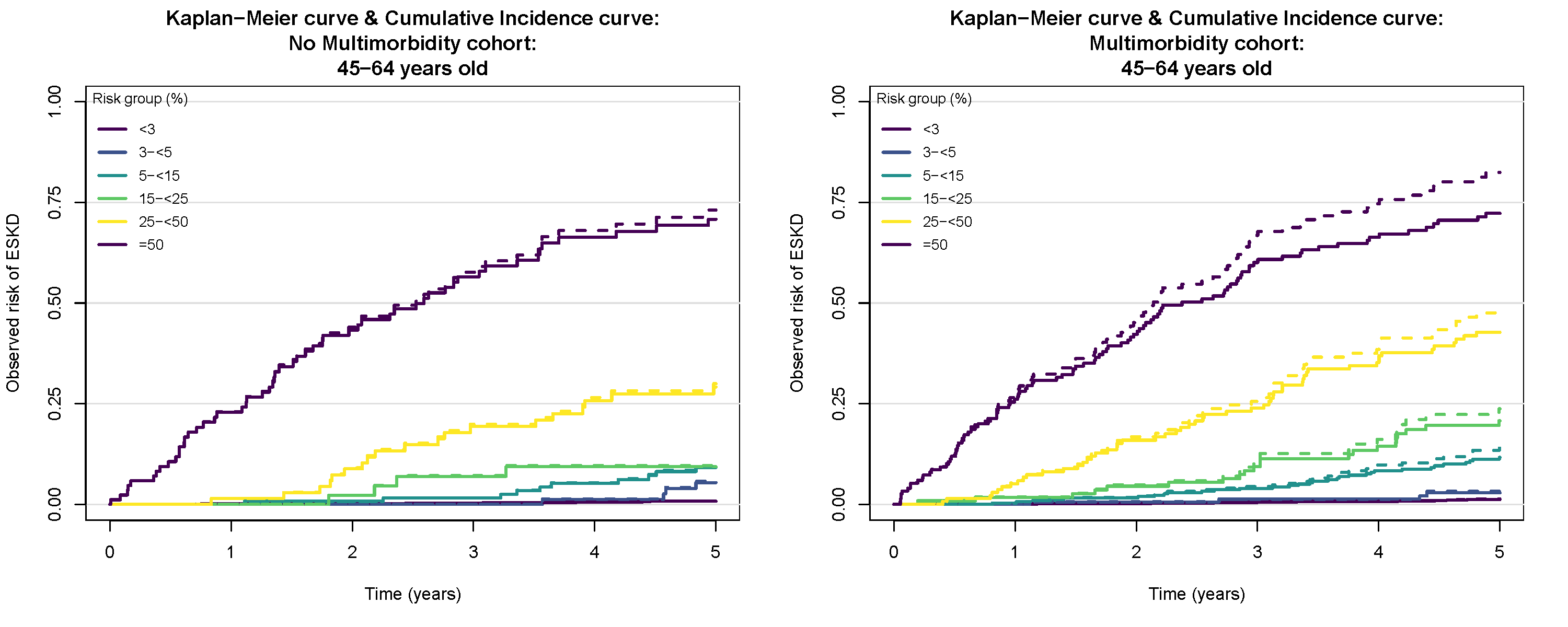


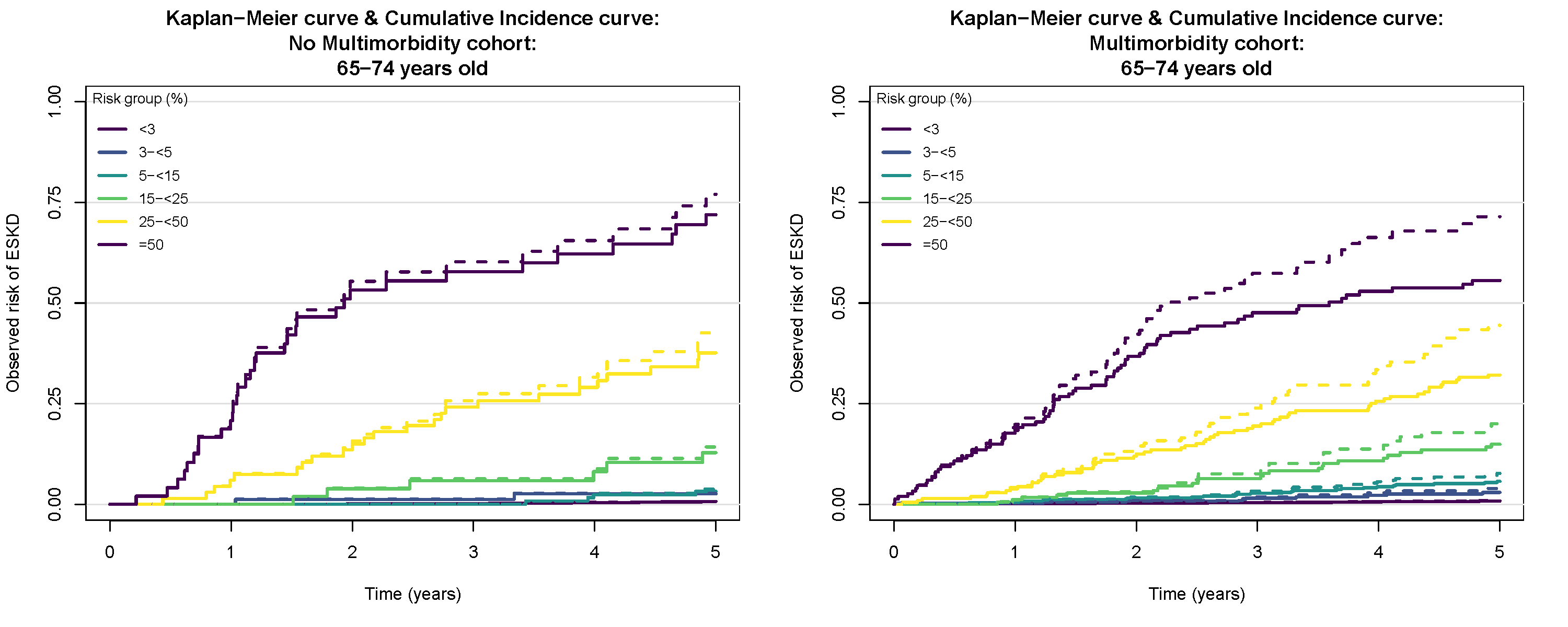


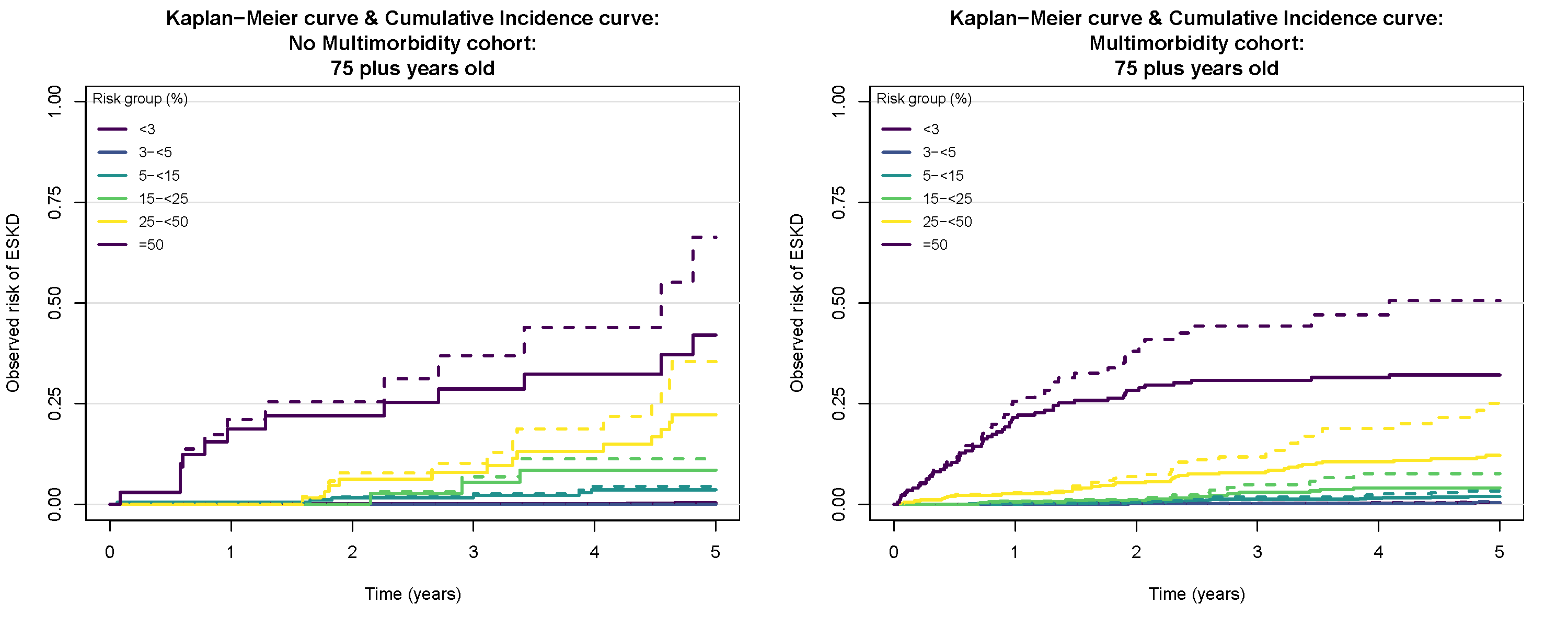


Supplementary Figure 16. Kaplan-Meier estimates (dashed lines) and Aalen-Johansen estimates (solid lines) cumulative incidence curves for kidney failure by KFRE risk groups in SCREAM – comparison by multimorbidity status and age groups


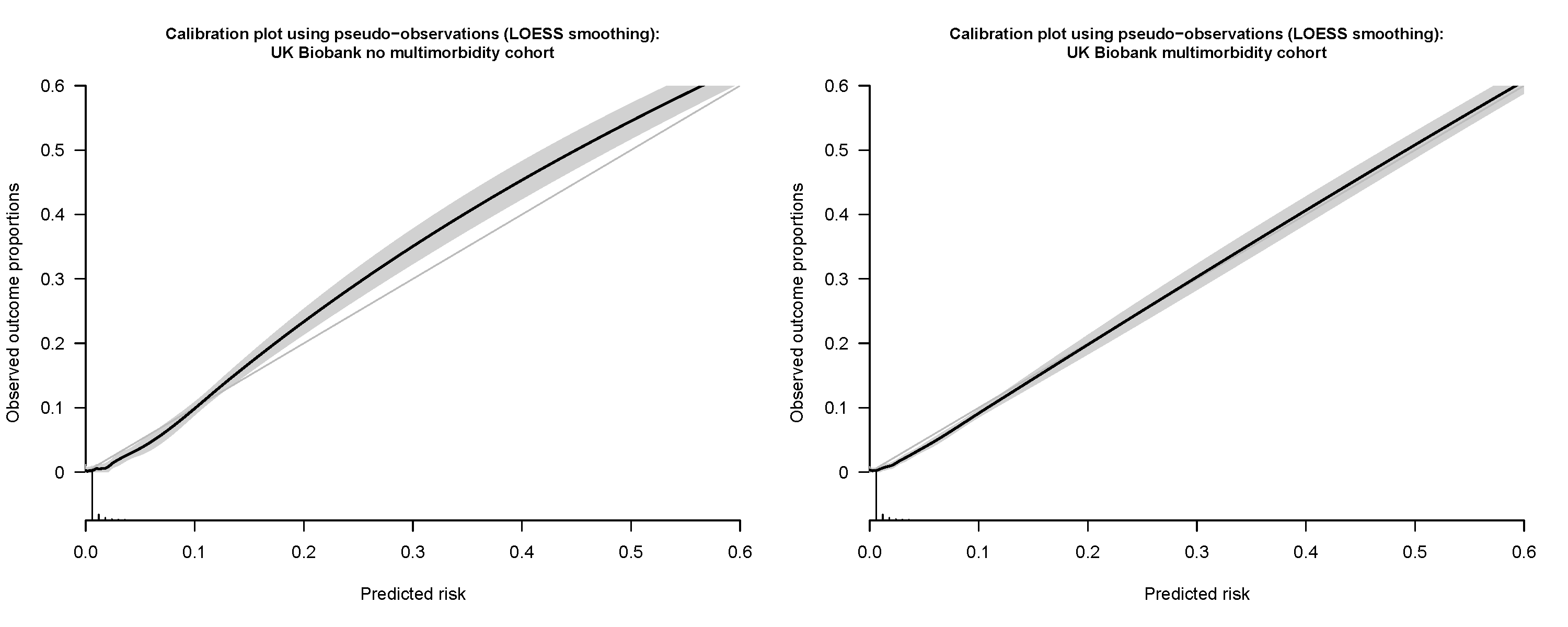


Supplementary Figure 17. Calibration plots with LOESS smoothing using cause-specific hazards approach and pseudo-observations for 5-year model (using eGFRcr) accounting for competing risk of mortality – UK Biobank cohort by multimorbidity status.


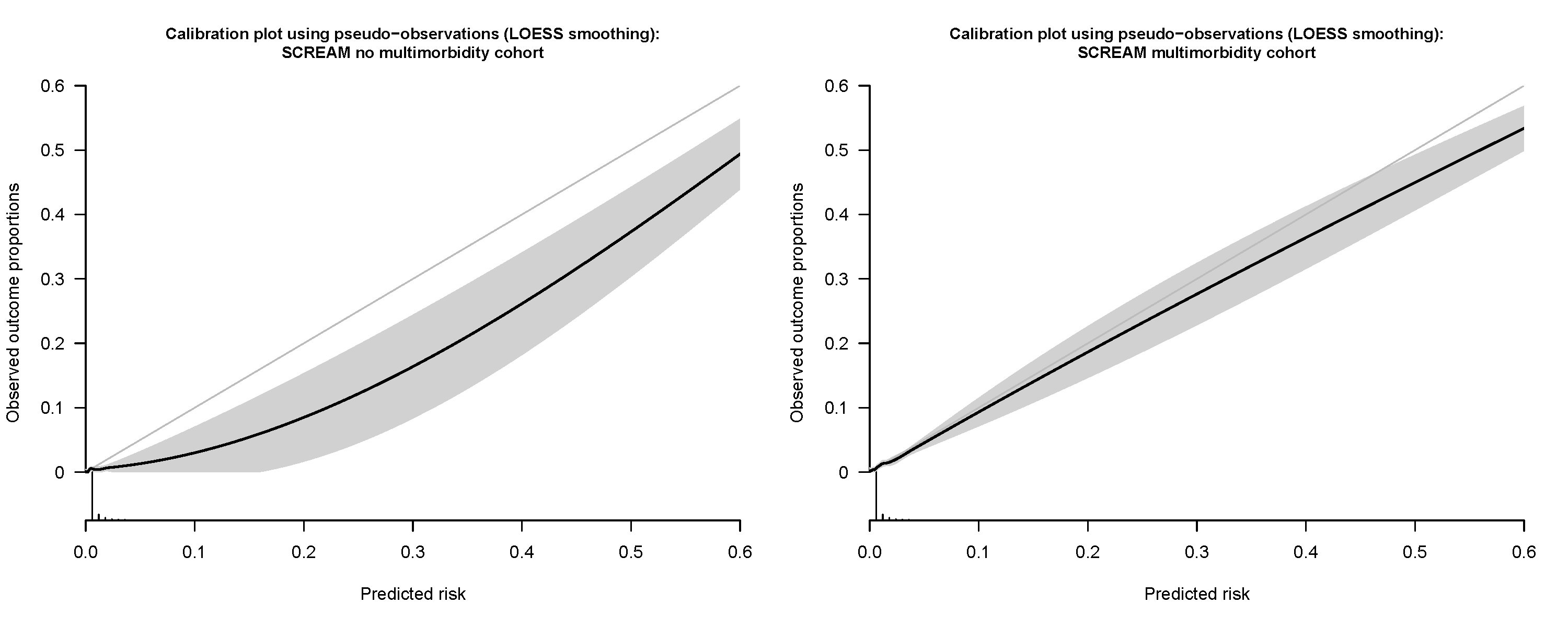


Supplementary Figure 18. Calibration plots with LOESS smoothing using cause-specific hazards approach and pseudo-observations for 5-year model (using eGFRcr) accounting for competing risk of mortality – SCREAM cohort by multimorbidity status.


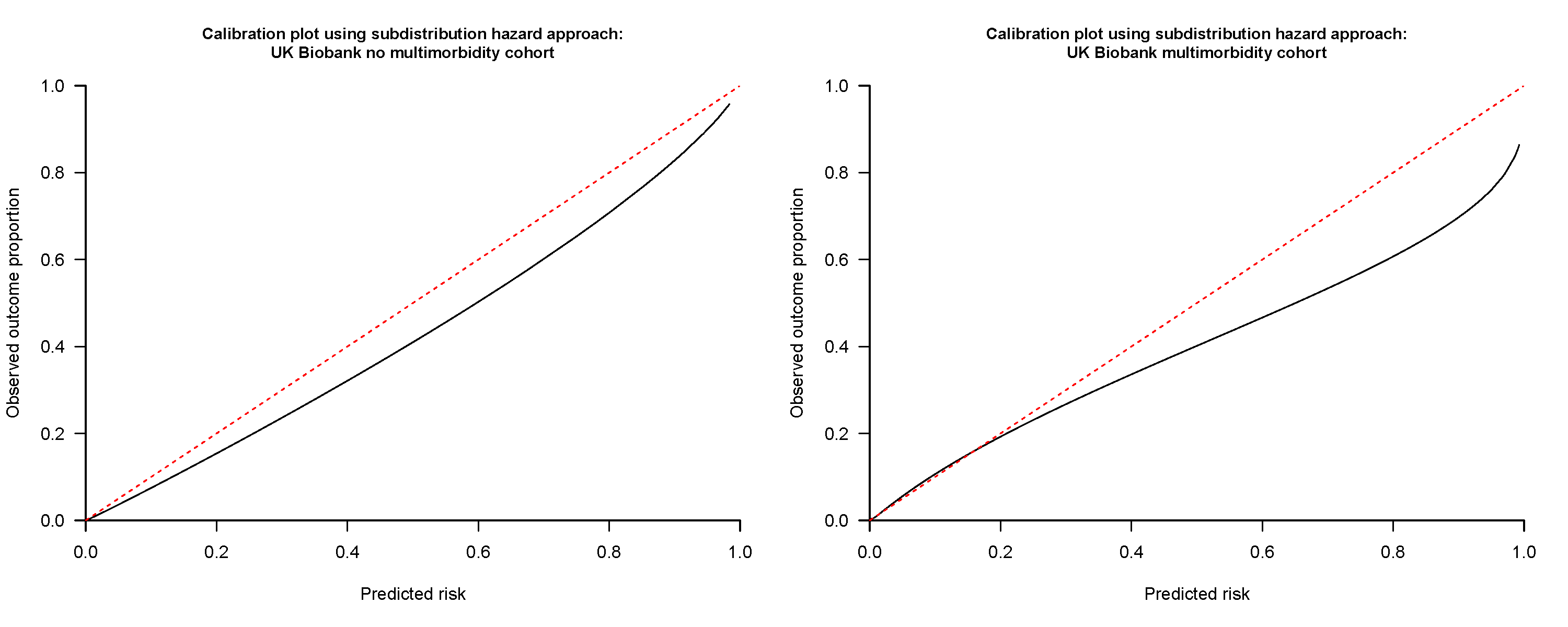


Supplementary Figure 19. Calibration plots using sub-distribution hazard approach for 5-year model (using eGFRcr) accounting for competing risk of mortality – UK Biobank cohort by multimorbidity status


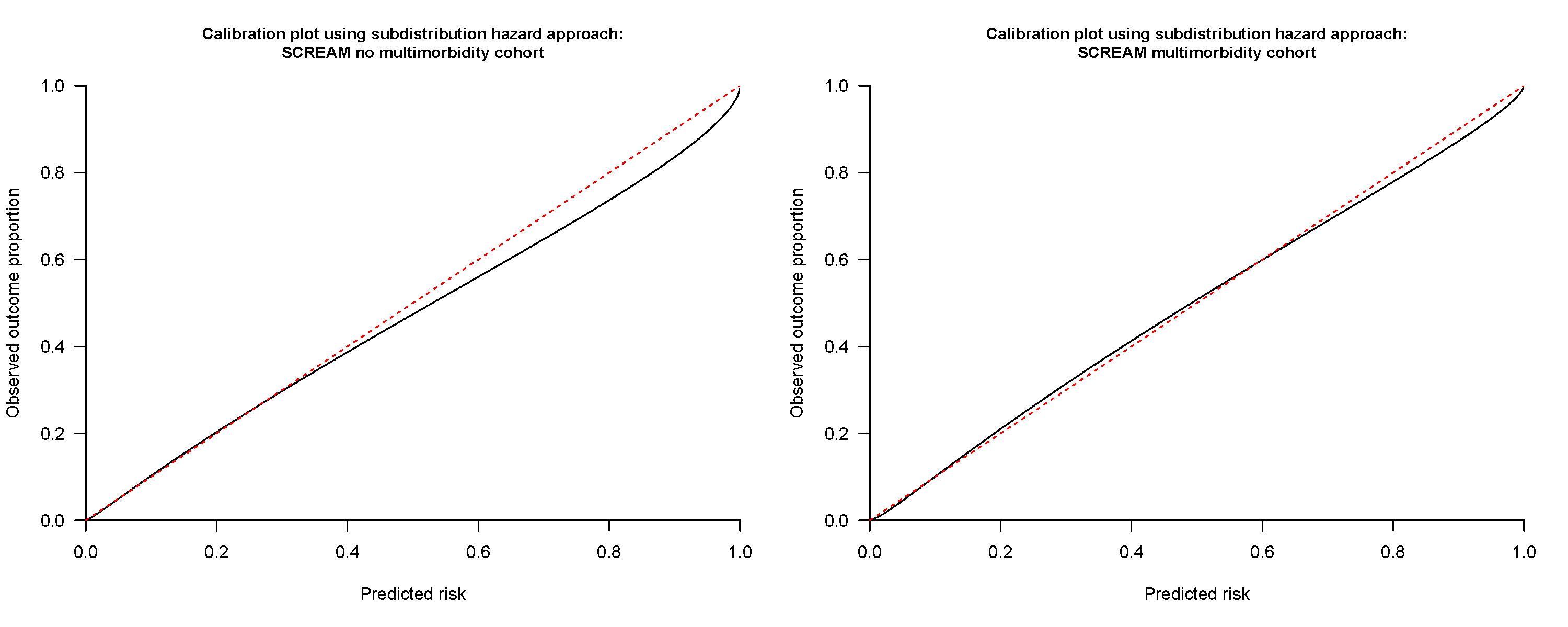


Supplementary Figure 20. Calibration plots using sub-distribution hazard approach for 5-year model (using eGFRcr) accounting for competing risk of mortality –SCREAM cohort by multimorbidity status

| Physical health conditions | Mental health conditions |
| --- | --- |
| Painful conditions | Depression |
| Hypertension | Anxiety* |
| Asthma | Alcohol problems* |
| Atrial Fibrillation | Other psychoactive substance abuse* |
| Coronary Heart Disease | Dementia |
| Dyspepsia* | Schizophrenia/bipolar disorder |
| Diabetes | Anorexia or bulimia* |
| Thyroid disorders |  |
| Connective tissue disorders |  |
| Chronic Obstructive Pulmonary Disease (COPD) |  |
| Irritable bowel syndrome |  |
| Treated constipation |  |
| Stroke/Transient Ischaemic Attack (TIA) |  |
| Chronic kidney disease |  |
| Diverticular disease* |  |
| Peripheral vascular disease |  |
| Heart failure |  |
| Prostate disorders |  |
| Glaucoma* |  |
| Epilepsy |  |
| Psoriasis/eczema |  |
| Inflammatory Bowel Disease |  |
| Migraine* |  |
| Chronic sinusitis* |  |
| Bronchiectasis* |  |
| Parkinson’s disease |  |
| Multiple Sclerosis |  |
| Viral Hepatitis |  |
| Chronic Liver disease |  |
| Osteoporosis* |  |
| Chronic fatigue syndrome* |  |
| Endometriosis* |  |
| Meniere’s disease* |  |
| Pernicious Anaemia* |  |
| Polycystic ovary* |  |
| Cancer |  |

*Included in UK Biobank LTCs only

Supplementary Table 1. Long term conditions included in multimorbidity definition

| Multimorbidity cluster | LTC included |
| --- | --- |
| Cardiometabolic | Hypertension, coronary heart disease, peripheral vascular disease, atrial fibrillation, heart failure, stroke/transient ischaemic attack, diabetes |
| Complex | Involvement of three or more LTCs from three or more body systems (neoplasm, haematological, endocrine/metabolic, mental health, neurological, ophthalmological, ontological, cardiovascular, respiratory, gastrointestinal, musculoskeletal, gynaecological, other) |
| Mixed physical and mental health | At least 1 physical and 1 mental health condition from Supplementary Table 1 |

Supplementary Table 2. Long term conditions included in multimorbidity clusters

| **Section/Topic** | **Item** | **Checklist Item** | **Page** |
| --- | --- | --- | --- |
| **Title and abstract** | | | |
| Title | 1 | Identify the study as developing and/or validating a multivariable prediction model, the target population, and the outcome to be predicted. | 1 |
| Abstract | 2 | Provide a summary of objectives, study design, setting, participants, sample size, predictors, outcome, statistical analysis, results, and conclusions. | 4-5 |
| **Introduction** | | | |
| Background and objectives | 3a | Explain the medical context (including whether diagnostic or prognostic) and rationale for developing or validating the multivariable prediction model, including references to existing models. | 6-8 |
|  | 3b | Specify the objectives, including whether the study describes the development or validation of the model or both. | 8 |
| **Methods** | | | |
| Source of data | 4a | Describe the study design or source of data (e.g., randomized trial, cohort, or registry data), separately for the development and validation data sets, if applicable. | 8-9 |
|  | 4b | Specify the key study dates, including start of accrual; end of accrual; and, if applicable, end of follow-up. | 8-9 |
| Participants | 5a | Specify key elements of the study setting (e.g., primary care, secondary care, general population) including number and location of centres. | 8-9 |
|  | 5b | Describe eligibility criteria for participants. | 9 |
|  | 5c | Give details of treatments received, if relevant. | N/A |
| Outcome | 6a | Clearly define the outcome that is predicted by the prediction model, including how and when assessed. | 11 |
|  | 6b | Report any actions to blind assessment of the outcome to be predicted. | N/A |
| Predictors | 7a | Clearly define all predictors used in developing or validating the multivariable prediction model, including how and when they were measured. | 9-10 |
|  | 7b | Report any actions to blind assessment of predictors for the outcome and other predictors. | N/A |
| Sample size | 8 | Explain how the study size was arrived at. | 11 |
| Missing data | 9 | Describe how missing data were handled (e.g., complete-case analysis, single imputation, multiple imputation) with details of any imputation method. | 10 |
| Statistical analysis methods | 10c | For validation, describe how the predictions were calculated. | 12 |
|  | 10d | Specify all measures used to assess model performance and, if relevant, to compare multiple models. | 12-13 |
|  | 10e | Describe any model updating (e.g., recalibration) arising from the validation, if done. | 13 |
| Risk groups | 11 | Provide details on how risk groups were created, if done. | 13 |
| Development vs. validation | 12 | For validation, identify any differences from the development data in setting, eligibility criteria, outcome, and predictors. | Table 1 |
| **Results** | | | |
| Participants | 13a | Describe the flow of participants through the study, including the number of participants with and without the outcome and, if applicable, a summary of the follow-up time. A diagram may be helpful. | Table 1  Supplementary figures 1-2 |
|  | 13b | Describe the characteristics of the participants (basic demographics, clinical features, available predictors), including the number of participants with missing data for predictors and outcome. | Table 1 |
|  | 13c | For validation, show a comparison with the development data of the distribution of important variables (demographics, predictors and outcome). | Table 1 |
| Model performance | 16 | Report performance measures (with CIs) for the prediction model. | 15-16, tables 2-3 + sup mats |
| Model-updating | 17 | If done, report the results from any model updating (i.e., model specification, model performance). | 17-18, table 5 |
| **Discussion** | | | |
| Limitations | 18 | Discuss any limitations of the study (such as nonrepresentative sample, few events per predictor, missing data). | 22-23 |
| Interpretation | 19a | For validation, discuss the results with reference to performance in the development data, and any other validation data. | 19-20 |
|  | 19b | Give an overall interpretation of the results, considering objectives, limitations, results from similar studies, and other relevant evidence. | 19-24 |
| Implications | 20 | Discuss the potential clinical use of the model and implications for future research. | 23-24 |
| **Other information** | | | |
| Supplementary information | 21 | Provide information about the availability of supplementary resources, such as study protocol, Web calculator, and data sets. |  |
| Funding | 22 | Give the source of funding and the role of the funders for the present study. | 25 |

Supplementary Table 3. Tripod checklist

| Long-term conditions count | Individuals | | RRT outcome events  n (%) | | | | Death (no prior RRT) events  n(%) | | | |
| --- | --- | --- | --- | --- | --- | --- | --- | --- | --- | --- |
|  | UK Biobank | SCREAM | UK Biobank | | SCREAM | | UK Biobank | | SCREAM | |
|  |  |  | **2-years** | **5-years** | **2-years** | **5-years** | **2-years** | **5-years** | **2-years** | **5-years** |
| 0 | 3200 | 4806 | 2 (0.1) | 3 (0.1) | 61 (1.3) | 138 (2.9) | 21 (0.7) | 75 (2.3) | 125 (2.6) | 314 (6.5) |
| 1 | 6291 | 7949 | 27 (0.4) | 67 (1.1) | 106 (1.3) | 237 (3.0) | 84 (1.3) | 254 (4.0) | 444 (5.6) | 973 (12.2) |
| 2 | 6538 | 9169 | 47 (0.7) | 100 (1.5) | 114 (1.2) | 259 (2.8) | 134 (2.1) | 395 (6.4) | 860 (9.4) | 1780 (19.4) |
| 3 | 4472 | 8329 | 37 (0.8) | 73 (1.6) | 94 (1.1) | 207 (2.5) | 111 (2.5) | 325 (7.3) | 1076 (12.9) | 2195 (26.4) |
| 4 | 2345 | 5983 | 23 (1.0) | 38 (1.6) | 58 (1.0) | 119 (2.0) | 92 (3.9) | 236 (10.1) | 1039 (17.4) | 1984 (33.2) |
| ≥5 | 1643 | 6666 | 14 (0.9) | 31 (1.9) | 78 (1.2) | 138 (2.1) | 57 (3.5) | 186 (11.3) | 1647 (24.7) | 2906 (43.6) |

Supplementary Table 4. Kidney failure and mortality events by long-term condition count groups

| Multimorbidity cluster | Individuals | | RRT outcome events  n (%) | | | | | Death (no prior RRT) events  n(%) | | | |
| --- | --- | --- | --- | --- | --- | --- | --- | --- | --- | --- | --- |
|  | UK Biobank | SCREAM | UK Biobank | | SCREAM | | UK Biobank | | | SCREAM | |
|  |  |  | **2-years** | **5-years** | **2-years** | **5-years** | **2-years** | | **5-years** | **2-years** | **5-years** |
| Cardiometabolic | 5375 | 17854 | 61 (1.1) | 126 (2.3) | 246 (1.4) | 492 (2.8) | 189 (3.5) | | 567 (10.5) | 3133 (17.5) | 6026 (33.8) |
| Complex | 6018 | 16330 | 53 (0.9) | 102 (1.7) | 157 (1.0) | 338 (2.1) | 177 (1.7) | | 532 (8.8) | 2996 (18.3) | 5561 (34.1) |
| Mixed mental/physical health | 1967 | 6175 | 12 (0.6) | 29 (1.5) | 60 (1.0) | 123 (2.0) | 55 (2.8) | | 157 (8.0) | 1145 (18.5) | 2118 (34.3) |

Supplementary Table 5. Kidney failure and mortality events by multimorbidity clusters

| eGFR equation used in KFRE | Performance measure for 2-year KFRE | Whole cohort | | Multimorbidity | | No multimorbidity | |
| --- | --- | --- | --- | --- | --- | --- | --- |
|  |  | UK Biobank | SCREAM | UK Biobank | SCREAM | UK Biobank | SCREAM |
| eGFRcys | AUC | 0.90 (0.86-0.93) | 0.89 (0.88-0.91) | 0.89 (0.86-0.93) | 0.90 (0.88-0.92) | 0.88 (0.80-0.97) | 0.95 (0.92-0.97) |
|  | C-index | 0.93 (0.92-0.95) | 0.94 (0.93-0.94) | 0.93 (0.90-0.94) | 0.93 (0.91-0.93) | 0.94 (0.91-0.97) | 0.96 (0.95-0.97) |
|  | O/E ratio | 1.74  (1.58-1.90) | 0.86  (0.77-0.95) | 1.75  (1.57-1.92) | 0.78  (0.67-0.88) | 1.75  (1.38-2.11) | 1.14  (0.99-1.29) |
|  | Calibration intercept | 0.23  (-0.02-0.48) | 0.10  (0.00-0.19) | 0.22  (-0.06-0.49) | -0.01  (-0.13-0.10) | 0.30  (-0.29-0.88) | 0.38  (0.22-0.54) |
|  | Calibration slope | 0.78  (0.66-0.90) | 1.49  (1.34-1.64) | 0.75 (0.62-0.89) | 1.47  (1.29-1.65) | 0.91  (0.58-1.25) | 1.48  (1.22-1.73) |
|  | Brier score | 0.005  (0.004-0.006) | 0.010  (0.009-0.011) | 0.007  (0.006-0.008) | 0.010  (0.009-0.011) | 0.003  (0.002-0.004) | 0.009  (0.008-0.010) |
|  | Scaled brier score (%) | 14.80  (10.00-19.29) | 28.33 (25.84-30.76) | 15.11  (9.83-20.19) | 24.82  (22.01-27.68) | 13.04  (3.18-22.83) | 35.67  (30.83-40.02) |
| eGFRcrcys | AUC | 0.89 (0.86-0.93) | 0.90 (0.89-0.91) | 0.89 (0.85-0.93) | 0.91 (0.88-0.93) | 0.88 (0.79-0.97) | 0.95 (0.87-0.92) |
|  | C-index | 0.94 (0.91-0.95) | 0.94 (0.93-0.95 | 0.93 (0.90-0.95 | 0.92 (0.91-0.94) | 0.93 (0.90-0.97) | 0.96 (0.95-0.97) |
|  | O/E ratio | 2.13  (1.97-2.29) | 1.06  (0.97-1.15) | 2.13  (1.96-2.31) | 1.01  (0.91-1.12) | 2.11  (1.74-2.47) | 1.19  (1.04-1.34) |
|  | Calibration intercept | 0.28  (0.02-0.55) | 0.28  (0.19-0.38) | 0.30  (0.01-0.60) | 0.23  (0.11-0.35) | 0.22  (-0.40-0.83) | 0.39  (0.23-0.55) |
|  | Calibration slope | 0.71  (0.59-0.82) | 1.45  (1.30-1.60) | 0.68  (0.56-0.80) | 1.44  (1.26-1.62) | 0.83  (0.51-1.14) | 1.44  (1.18-1.70) |
|  | Brier score | 0.005  (0.004-0.006) | 0.010  (0.009-0.011) | 0.007  (0.006-0.008) | 0.010  (0.009-0.011) | 0.003  (0.002-0.004) | 0.009  (0.008-0.010) |
|  | Scaled brier score (%) | 14.81  (10.21-19.25) | 28.33  (28.49-33.54) | 15.12  (10.41-21.03) | 24.82  (24.55-30.68) | 13.04  (2.66-25.61) | 35.67  (33.84-43.58) |

Supplementary Table 6. Performance measures for validation of UK-calibrated KFRE by cohort, cystatin-based eGFR equation and multimorbidity status at 2-years

AUC = Area under the receiver operator characteristic curves

| eGFR equation used in KFRE | Performance measure for 5-year KFRE | Whole cohort | | Multimorbidity | | No multimorbidity | | |
| --- | --- | --- | --- | --- | --- | --- | --- | --- |
|  |  | UK Biobank | SCREAM | UK Biobank | SCREAM | UK Biobank | SCREAM |  |
| eGFRcys | AUC | 0.90 (0.86-0.93) | 0.88 (0.87-0.90) | 0.91 (0.88-0.94) | 0.88 (0.86-0.89) | 0.89 (0.84-0.95) | 0.90 (0.87-0.92) |  |
|  | C-index | 0.94 (0.92-0.95) | 0.93 (0.92-0.93) | 0.93 (0.88-0.94) | 0.93 (0.91-0.92) | 0.95 (0.93-0.97) | 0.95 (0.94-0.95) |  |
|  | O/E ratio | 1.30  (1.19-1.41) | 0.81  (0.75-0.87) | 1.28  (1.15-1.40) | 0.73  (0.65-0.80) | 1.43  (1.20-1.67) | 1.12  (1.02-1.22) |  |
|  | Calibration intercept | 0.21  (0.06-0.363 | -0.31  (-0.38- -0.23) | 0.17  (-0.01-0.35) | -0.44 (-0.53—0.34) | 0.38  (0.07-0.70) | 0.09  (-0.05-0.23) |  |
|  | Calibration slope | 0.87  (0.78-0.96) | 1.02  (0.95-1.09) | 0.84  (0.75-0.94) | 1.02  (0.94-1.11) | 1.03  (0.78-1.28) | 0.99  (0.87-1.10) |  |
|  | Brier score | 0.009  (0.008-0.010) | 0.023  (0.022-0.025) | 0.011  (0.010-0.013) | 0.024  (0.022-0.026) | 0.005  (0.004-0.007) | 0.022  (0.020-0.024) |  |
|  | Scaled brier score (%) | 30.52  (25.64-35.06) | 35.43  (32.54-38.15) | 31.05  (25.72-36.21) | 33.34  (30.02-36.55) | 28.03  (19.06-36.89) | 39.30  (33.88-43.66) |  |
| eGFRcrcys | AUC | 0.89 (0.89-0.93) | 0.89 (0.87-0.90) | 0.91 (0.88-094) | 0.88 (0.87-0.90) | 0.90 (0.84-0.95) | 0.90 (0.87-0.92) |  |
|  | C-index | 0.94 (0.92-0.95) | 0.93 (0.92-0.94) | 0.93 (0.91-0.94) | 0.92 (0.91-0.93) | 0.96 (0.94-0.97) | 0.95 (0.94-0.96) |  |
|  | O/E ratio | 1.69  (1.58-1.80) | 1.03  (0.97-1.09) | 1.65  (1.52-1.77) | 0.96  (0.88-1.03) | 1.86  (1.63-2.10) | 1.22  (1.12-1.32) |  |
|  | Calibration intercept | 0.46  (0.29-0.63) | -0.04  (-0.12-0.04) | 0.40  (0.21-0.59) | -0.13 (-0.23- -0.03) | 0.70  (0.37-1.02) | 0.18  (0.04-0.33) |  |
|  | Calibration slope | 0.79  (0.71-0.87) | 1.01  (0.93-1.08) | 0.77  (0.68-0.86) | 1.01  (0.92-1.10) | 0.96  (0.72-1.21) | 0.96  (0.84-1.08) |  |
|  | Brier score | 0.009  (0.008-0.010) | 0.022  (0.021-0.024) | 0.011  (0.010-0.013) | 0.023  (0.021-0.024) | 0.005  (0.004-0.007) | 0.021  (0.019-0.024) |  |
|  | Scaled brier score (%) | 31.10  (26.58-35.75) | 38.28  (35.60-40.70) | 31.46  (26.42-36.61) | 36.75  (33.52-39.70) | 29.27  (19.94-38.24) | 41.51  (36.92-45.94) |  |

Supplementary Table 7. Performance measures for validation of UK-calibrated KFRE by cohort, cystatin-based eGFR equation and multimorbidity status at 5-years

AUC = Area under the receiver operator characteristic curves

| eGFR equation used in KFRE | Performance measure for 2-year KFRE (95% CI) | Long term condition count | | | | | |
| --- | --- | --- | --- | --- | --- | --- | --- |
|  |  | **0 (n=4806)** | **1 (n=7949)** | **2 (n=9169)** | **3 (n=8329)** | **4 (n=5983)** | **≥5 (n=6666)** |
| eGFRcr | AUC | 0.90 (0.88-0.93) | 0.88 (0.87-0.913 | 0.90 (0.88-0.931) | 0.90 (0.87-0.93) | 0.90 (0.88-0.93) | 0.90 (0.87-0.93) |
|  | C-index | 0.95 (0.92-0.98) | 0.95 (0.94-0.97) | 0.91 (0.89-0.94) | 0.92 (0.90-0.94) | 0.93 (0.90-0.96) | 0.93 (0.92-0.95) |
|  | O/E ratio | 1.13 (0.88-1.38) | 1.08 (0.89-1.27) | 1.10 (0.92-1.28) | 1.07 (0.87-1.27) | 1.04 (0.78-1.30) | 1.16 (0.94-1.39) |
|  | Calibration intercept | 0.31 (0.05-0.56) | 0.23 (0.02-0.44) | 0.25 (0.04-0.46) | 0.21 (-0.03-0.44) | 0.27 (-0.03-0.56) | 0.29 (0.03-0.55) |
|  | Calibration slope | 1.50 (1.07-1.93) | 1.39 (1.08-1.69) | 1.27 (1.01-1.53) | 1.28 (0.98-1.57) | 1.71 (1.09-2.33) | 1.24 (0.90-1.59) |
|  | Brier score | 0.008 (0.006-0.010) | 0.009 (0.007-0.011) | 0.010 (0.008-0.011) | 0.010 (0.008-0.011) | 0.008 (0.006-0.010) | 0.012 (0.009-0.014) |
|  | Scaled brier score (%) | 42.71 (33.76-50.9) | 37.31 (31.30-43.80) | 29.99 (24.28-35.62) | 26.32 (19.62-32.24) | 29.31 (21.82-36.47) | 23.13 (18.40-28.45) |
| eGFRcys | AUC | 0.90 (0.88-0.92) | 0.90 (0.86-0.93) | 0.90 (0.88-0.92) | 0.90 (0.86-0.93) | 0.90 (0.88-0.92) | 0.90 (0.86-0.93) |
|  | C-index | 0.96 (0.95-0.97) | 0.96 (0.95-0.97) | 0.92 (0.90-0.94) | 0.92 (0.89-0.94) | 0.93 (0.91-0.95) | 0.93 (0.91-0.94) |
|  | O/E ratio | 1.29 (1.04-1.54) | 1.07 (0.89-1.26) | 0.94 (0.76-1.13) | 0.80 (0.60-1.00) | 0.67 (0.41-0.92) | 0.69 (0.46-0.91) |
|  | Calibration intercept | 0.48 (0.23-0.73) | 0.33 (0.12-0.53) | 0.16 (-0.04-0.36) | -0.02 (-0.24-0.21) | -0.06 (-0.34-0.22) | -0.18 (-0.43-0.08) |
|  | Calibration slope | 1.45 (1.04-1.86) | 1.47 (1.15-1.80) | 1.52 (1.21-1.82) | 1.41 (1.10-1.73) | 1.67 (1.16-2.17) | 1.29 (0.93-1.64) |
|  | Brier score | 0.009 (0.007-0.011) | 0.009 (0.008-0.011) | 0.010 (0.008-0.012) | 0.010 (0.008-0.012) | 0.008 (0.006-0.010) | 0.012 (0.009-0.014) |
|  | Scaled brier score (%) | 35.63 (28.69-42.03) | 35.67 (30.12-40.90) | 27.52 (22.52-32.59) | 22.23 (16.07-27.85) | 28.18 (21.32-34.60) | 21.43 (15.62-27.01) |
| eGFRcrcys | AUC | 0.91 (0.88-0.93) | 0.90 (0.87-0.93) | 0.91 (0.88-0.93) | 0.90 (0.87-0.93) | 0.91 (0.88-0.93) | 0.90 (0.87-0.93) |
|  | C-index | 0.96 (0.94-0.98) | 0.96 (0.95-0.97) | 0.92 (0.89-0.94) | 0.92 (0.90-0.95) | 0.94 (0.92-0.96) | 0.93 (0.92-0.95) |
|  | O/E ratio | 1.27 (1.02-1.52) | 1.15 (0.97-1.34) | 1.12 (0.93-1.30) | 1.01 (0.81-1.22) | 0.92 (0.66-1.17) | 0.98 (0.75-1.20) |
|  | Calibration intercept | 0.44 (0.19-0.70) | 0.36 (0.15-0.57) | 0.31 (0.10-0.51) | 0.20 (-0.03-0.43) | 0.22 (-0.06-0.50) | 0.18 (-0.08-0.43) |
|  | Calibration slope | 1.48 (1.04-1.92) | 1.41 (1.10-1.73) | 1.42 (1.12-1.72) | 1.35 (1.04-1.66) | 1.87 (1.23-2.51) | 1.32 (0.95-1.70) |
|  | Brier score | 0.008 (0.006-0.010) | 0.009 (0.007-0.011) | 0.010 (0.008-0.011) | 0.010 (0.008-0.011) | 0.008 (0.006-0.010) | 0.011 (0.009-0.014) |
|  | Scaled brier score (%) | 40.89 (33.26-48.85) | 37.70 (31.58-43.89) | 30.14 (24.28-35.05) | 25.81 (19.21-31.33) | 30.63 (23.71-37.40) | 23.79 (18.52-28.88) |

Supplementary Table 8. Performance measures for validation of UK-calibrated KFRE by eGFR equation and LTC count in SCREAM cohort at 2-years

AUC = Area under the receiver operator characteristic curves

| eGFR equation used in KFRE | Performance measure for 5-year KFRE (95% CI) | Long term condition count | | | | | |
| --- | --- | --- | --- | --- | --- | --- | --- |
|  |  | **0 (n=4806)** | **1 (n=7949)** | **2 (n=9169)** | **3 (n=8329)** | **4 (n=5983)** | **≥5 (n=6666)** |
| eGFRcr | AUC | 0.88 (0.86-0.90) | 0.87 (0.85-0.89) | 0.88 (0.86-0.89) | 0.87 (0.85-0.89) | 0.88 (0.86-0.90) | 0.87 (0.85-0.89) |
|  | C-index | 0.93 (0.91-0.95) | 0.94 (0.93-0.95) | 0.91 (0.90-0.93) | 0.91 (0.89-0.93) | 0.92 (0.89-0.94) | 0.92 (0.90-0.94) |
|  | O/E ratio | 1.18 (1.02-1.35) | 1.13 (1.00-1.26) | 1.14 (1.02-1.26) | 1.08 (0.94-1.21) | 0.97 (0.78-1.15) | 0.96 (0.78-1.13) |
|  | Calibration intercept | 0.01 (-0.24-0.26) | 0.08 (-0.11-0.26) | 0.11 (-0.07-0.28) | -0.01 (-0.20-0.19) | -0.26 (-0.51- -0.02) | -0.24 (-0.47- -0.01) |
|  | Calibration slope | 0.89 (0.72-1.05) | 0.97 (0.82-1.12) | 1.05 (0.88-1.21) | 1.02 (0.85-1.19) | 0.98 (0.76-1.21) | 0.89 (0.71-1.07) |
|  | Brier score | 0.021 (0.017-0.025) | 0.023 (0.020-0.026) | 0.023 (0.020-0.026) | 0.024 (0.020-0.027) | 0.025 (0.020-0.030) | 0.024 (0.020-0.029) |
|  | Scaled brier score (%) | 39.30 (30.39-46.88) | 39.56 (33.18-45.51) | 40.35 (34.47-44.99) | 33.80 (27.69-39.81) | 18.65 (24.43-39.23) | 30.56 (22.58-38.25) |
| eGFRcys | AUC | 0.88 (0.86-0.89) | 0.86 (0.84-0.89) | 0.88 (0.86-0.89) | 0.86 (0.84-0.89) | 0.87 (0.86-0.89) | 0.86 (0.84-0.89) |
|  | C-index | 0.95 (0.93-0.96) | 0.95 (0.94-0.96) | 0.92 (0.91-0.94) | 0.91 (0.89-0.93) | 0.92 (0.90-0.94) | 0.92 (0.90-0.93) |
|  | O/E ratio | 1.24 (1.07-1.40) | 1.07 (0.94-1.19) | 0.93 (0.81-1.05) | 0.78 (0.64-0.91) | 0.61 (0.42-0.79) | 0.56 (0.39-0.74) |
|  | Calibration intercept | 0.14 (-0.08-0.36) | 0.07 (-0.10-0.24) | -0.13 (-0.29-0.03) | -0.41 (-0.58- -0.23) | -0.59 (-0.82- -0.37) | -0.70 (-0.91- -0.49) |
|  | Calibration slope | 0.98 (0.78-1.18) | 0.98 (0.84-1.13) | 1.02 (0.87-1.16) | 0.99 (0.84-1.14) | 1.12 (0.88-1.35) | 0.99 (0.80-1.18) |
|  | Brier score | 0.021 (0.017-0.025) | 0.023 (0.020-0.026) | 0.024 (0.021-0.027) | 0.025 (0.022-0.029) | 0.024 (0.020-0.029) | 0.024 (0.019-0.028) |
|  | Scaled brier score (%) | 39.12 (31.51-46.93) | 39.31 (33.47-44.40) | 37.32 (31.98-42.25) | 29.39 (22.30-35.16) | 21.46 (21.97-38.66) | 33.17 (24.49-40.78) |
| eGFRcrcys | AUC | 0.88 (0.87-0.90) | 0.87 (0.85-0.90) | 0.88 (0.87-0.90) | 0.87 (0.85-0.90) | 0.88 (0.87-0.90) | 0.87 (0.85-0.90) |
|  | C-index | 0.95 (0.93-0.96) | 0.95 (0.94-0.96) | 0.92 (0.91-0.94) | 0.91 (0.90-0.93) | 0.92 (0.91-0.94) | 0.92 (0.90-0.94) |
|  | O/E ratio | 1.29 (1.13-1.46) | 1.19 (1.06-1.31) | 1.13 (1.01-1.25) | 1.00 (0.86-1.14) | 0.84 (0.66-1.03) | 0.79 (0.62-0.97) |
|  | Calibration intercept | 0.17 (-0.07-0.41) | 0.20 (0.02-0.38) | 0.11 (-0.06-0.28) | -0.10 (-0.28-0.09) | -0.30 (-0.54- -0.07) | -0.36 (-0.58- -0.14) |
|  | Calibration slope | 0.90 (0.72-1.08) | 1.00 (0.84-1.16) | 1.02 (0.87-1.17) | 0.98 (0.82-1.14) | 1.01 (0.80-1.23) | 0.96 (0.77-1.16) |
|  | Brier score | 0.020 (0.016-0.024) | 0.022 (0.019-0.026) | 0.023 (0.019-0.026) | 0.024 (0.020-0.027) | 0.025 (0.020-0.030) | 0.023 (0.019-0.028) |
|  | Scaled brier score (%) | 41.35 (33.91-48.67) | 41.55 (35.75-47.18) | 40.96 (35.71-46.16) | 33.79 (27.46-39.46) | 19.98 (27.27-42.00) | 34.28 (26.95-41.46) |

Supplementary Table 9. Performance measures for validation of UK-calibrated KFRE by eGFR equation and LTC count in SCREAM cohort at 5-years

AUC = Area under the receiver operator characteristic curves

| eGFR equation used in KFRE | Performance measure for 2-year KFRE (95% CI) | Multimorbidity cluster | | | |
| --- | --- | --- | --- | --- | --- |
|  |  | **Cardiometabolic** | | **Complex** | |
|  |  | **UK Biobank**  **n=5375** | **SCREAM**  **n=17854** | **SCREAM**  **n=16330** |  |
| eGFRcr | AUC | 0.84 (0.77-0.90) | 0.90 (0.87-0.93) | 0.93 (0.91-0.95) | |
|  | C-index | 0.87 (0.82-0.91) | 0.92 (0.91-0.93) | 0.93 (0.91-0.94) | |
|  | O/E ratio | 2.06 (1.81-2.31) | 1.11 (0.98-1.23) | 1.03 (0.88-1.19) | |
|  | Calibration intercept | 0.15 (-0.28-0.59) | 0.27 (0.13-0.42) | 0.19 (0.01-0.37) | |
|  | Calibration slope | 0.64 (0.46-0.81) | 1.31 (1.11-1.51) | 1.37 (1.11-1.63) | |
|  | Brier score | 0.010 (0.007-0.012) | 0.012 (0.010-0.013) | 0.009 (0.007-0.010) | |
|  | Scaled brier score (%) | 13.70 (5.76-21.36) | 26.84 (23.23-30.59) | 25.71 (20.89-30.59) | |
| eGFRcys | AUC | 0.86 (0.80-0.93) | 0.90 (0.87-0.93) | 0.93 (0.91-0.95) |  |
|  | C-index | 0.90 (0.87-0.94) | 0.92 (0.91-0.93) | 0.92 (0.90-0.94) |  |
|  | O/E ratio | 1.51 (1.26-1.76) | 0.78 (0.65-0.90) | 0.65 (0.49-0.80) |  |
|  | Calibration intercept | 0.04 (-0.35-0.43) | -0.02 (-0.16-0.12) | -0.17 (-0.34-0.00) |  |
|  | Calibration slope | 0.71 (0.52-0.91) | 1.34 (1.15-1.53) | 1.58 (1.29-1.87) |  |
|  | Brier score | 0.010 (0.007-0.012) | 0.012 (0.011-0.014) | 0.009 (0.00-0.010) |  |
|  | Scaled brier score (%) | 14.57 (6.44-21.90) | 23.85 (20.47-27.16) | 23.57 (18.68-28.05) |  |
| eGFRcrcys | AUC | 0.86 (0.79-0.92) | 0.90 (0.88-0.93) | 0.93 (0.91-0.95) |  |
|  | C-index | 0.90 (0.86-0.94) | 0.92 (0.91-0.94) | 0.93 (0.91-0.94) |  |
|  | O/E ratio | 1.92 (1.67-2.17) | 1.02 (0.89-1.14) | 0.90 (0.74-1.05) |  |
|  | Calibration intercept | 0.16 (-0.25-0.57) | 0.24 (0.10-0.38) | 0.13 (-0.05-0.30) |  |
|  | Calibration slope | 0.65 (0.47-0.83) | 1.38 (1.18-1.59) | 1.52 (1.23-1.80) |  |
|  | Brier score | 0.010 (0.007-0.12) | 0.012 (0.011-0.013) | 0.009 (0.007-0.010) |  |
|  | Scaled brier score (%) | 14.53 (6.44-21.76) | 26.77 (23.52-30.39) | 26.54 (22.18-30.39) |  |

Supplementary Table 10. Performance measures by eGFR equations and multimorbidity clusters at 2-years

AUC = Area under the receiver operator characteristic curves

| eGFR equation used in KFRE | Performance measure for 5-year KFRE (95% CI) | Multimorbidity cluster | | |
| --- | --- | --- | --- | --- |
|  |  | **Cardiometabolic** | | **Complex** |
|  |  | **UK Biobank**  **n=5375** | **SCREAM**  **n=17854** | **SCREAM**  **n=16330** |
| eGFRcr | AUC | 0.87 (0.83-0.91) | 0.88 (0.85-0.90) | 0.87 (0.84-0.89) |
|  | C-index | 0.88 (0.85-0.91) | 0.91 (0.90-0.92) | 0.92 (0.90-0.93) |
|  | O/E ratio | 1.72 (1.54-1.89) | 1.01 (0.92-1.10) | 1.04 (0.93-1.15) |
|  | Calibration intercept | 0.49 (0.22-0.76) | -0.09 (-0.21-0.03) | -0.11 (-0.26-0.04) |
|  | Calibration slope | 0.82 (0.68-0.97) | 1.01 (0.90-1.12) | 0.96 (0.83-1.08) |
|  | Brier score | 0.016 (0.013-0.020) | 0.026 (0.024-0.029) | 0.022 (0.020-0.025) |
|  | Scaled brier score (%) | 31.33 (23.59-38.03) | 35.42 (31.53-39.33) | 32.36 (27.77-36.71) |
| eGFRcys | AUC | 0.87 (0.82-0.92) | 0.87 (0.85-0.89) | 0.86 (0.84-0.89) |
|  | C-index | 0.90 (0.88-0.93) | 0.91 (0.90-0.92) | 0.91 (0.90-0.93) |
|  | O/E ratio | 1.17 (0.99-1.34) | 0.69 (0.60-0.78) | 0.64 (0.53-0.75) |
|  | Calibration intercept | 0.08 (-0.16-0.33) | -0.45 (-0.56- -0.34) | -0.58 (-0.71- -0.44) |
|  | Calibration slope | 0.83 (0.69-0.97) | 1.05 (0.94-1.16) | 1.03 (0.90-1.16) |
|  | Brier score | 0.016 (0.013-0.019) | 0.026 (0.023-0.029) | 0.023 (0.020-0.025) |
|  | Scaled brier score (%) | 31.22 (23.84-39.31) | 35.45 (31.28-39.34) | 30.56 (25.33-35.23) |
| eGFRcrcys | AUC | 0.87 (0.82-0.92) | 0.88 (0.86-0.90) | 0.87 (0.84-0.90) |
|  | C-index | 0.90 (0.87-0.93) | 0.92 (0.91-0.93) | 0.92 (0.91-0.93) |
|  | O/E ratio | 1.55 (1.38-1.72) | 0.91 (0.82-1.00) | 0.89 (0.78-1.00) |
|  | Calibration intercept | 0.41 (0.15-0.66) | -0.15 (-0.27- -0.04) | -0.23 (-0.38- -0.09) |
|  | Calibration slope | 0.79 (0.6-0.93) | 1.04 (0.93-1.15) | 0.98 (0.85-1.10) |
|  | Brier score | 0.016 (0.013-0.019) | 0.025 (0.023-0.028) | 0.022 (0.019-0.024) |
|  | Scaled brier score (%) | 31.53 (23.48-39.21) | 37.91 (34.45-41.57) | 34.03 (29.44-38.42) |

Supplementary Table 11. Performance measures by eGFR equations and multimorbidity clusters at 5-years

AUC = Area under the receiver operator characteristic curves

| Time frame | Cumulative incidence of kidney failure - Estimate (95% CI) | | | |
| --- | --- | --- | --- | --- |
|  | **UK Biobank** | | **SCREAM** | |
|  | **No Multimorbidity** | **Multimorbidity** | **No Multimorbidity** | **Multimorbidity** |
| 1-year | 0.0016 (0.0008-0.0024) | 0.0033 (0.0024-0.0043) | 0.0070 (0.0055-0.0085) | 0.0059 (00.51-0.0068) |
| 2-year | 0.0028 (0.0018-0.0039) | 0.0077 (0.0063-0.0091) | 0.0140 (0.0119-0.0161) | 0.0122 (0.0109-0.0135) |
| 3-year | 0.0046 (0.0033-0.0060) | 0.0107 (0.0091-0.0124) | 0.0197 (0.0172-0.0223) | 0.0184 (0.0167-0.0200) |
| 4-year | 0.0061 (0.0045-0.0077) | 0.0129 (0.0111-0.0147) | 0.0268 (0.0238-0.0299) | 0.0240 (0.0221-0.0259) |
| 5-year | 0.0074 (0.0057-0.0091) | 0.0161 (0.0141-0.0181) | 0.0356 (0.0320-0.0391) | 0.0295 (0.0274-0.0316) |

Supplementary Table 12. Cumulative incidence of kidney failure by multimorbidity status

| Time frame | Cumulative incidence of mortality - Estimate (95% CI) | | | |
| --- | --- | --- | --- | --- |
|  | **UK Biobank** | | **SCREAM** | |
|  | **No Multimorbidity** | **Multimorbidity** | **No Multimorbidity** | **Multimorbidity** |
| 1-year | 0.0043 (0.0030-0.0056) | 0.0106 (0.0090-0.0122) | 0.0232 (0.0205-0.0258) | 0.0848 (0.0815-0.0880 |
| 2-year | 0.0104 (0.0084-0.0125) | 0.0241 (0.0216-0.0265) | 0.0428 (0.0391-0.0464) | 0.1551 (0.1508-0.1593) |
| 3-year | 0.0173 (0.0147-0.0199) | 0.0402 (0.0371-0.0433) | 0.0632 (0.0588-0.0677) | 0.2212 (0.2162-0.2262) |
| 4-year | 0.0234 (0.0203-0.0264) | 0.0544 (0.0508-0.0580) | 0.0843 (0.0790-0.0895) | 0.2856 (0.2800-0.2912) |
| 5-year | 0.0334 (0.0298-0.0370) | 0.0715 (0.0673-0.0756) | 0.0111 (0.1050-0.1171) | 0.3400 (0.3339-0.3460) |

Supplementary Table 13. Cumulative incidence of mortality by multimorbidity status

| **Covariate** | **Hazard ratio (95% CI)** | |
| --- | --- | --- |
|  | **Kidney failure** | **Morality** |
| Age | 0.45 (0.43-0.48) | 3.73 (3.61-3.85) |
| eGFR | 0.10 (0.09-0.10) | 0.87 (0.85-0.89) |
| lnACR | 4.51 (3.83-5.31) | 1.36 (1.31-1.41) |
| Sex (male) | 3.68 (3.31-4.08) | 1.05 (1.01-1.10) |

Supplementary Table 14. Hazard ratio and 95% confidence intervals from cause-specific Cox model for kidney failure and mortality

|  | **KFRE risk groups (as calculated by 5-year KFRE) – UK Biobank** | | | | | |
| --- | --- | --- | --- | --- | --- | --- |
|  | **<3%**  **n = 23800** | **3- <5%**  **n = 176** | **5- <15%**  **n = 236** | **15- <25%**  **n = 78** | **25- <50%**  **n = 89** | **≥50%**  **n= 110** |
| **Age** | 62.9 ± 5.5 | 61.4 ± 7.0 | 61.0 ± 6.9 | 59.1 ± 7.5 | 59.5 ± 7.8 | 59.3 ± 7.4 |
| **eGFRcr mean (SD)** | 66.7 ± 14.8 | 38.0 ± 6.8 | 32.8 ± 6.7 | 26.5 ± 6.2 | 22.7 ± 5.7 | 13.0 ± 6.1 |
| **eGFRcys mean (SD)** | 56.5 ± 12.2 | 35.9 ± 10.1 | 30.2 ± 7.7 | 26.7 ± 7.2 | 22.5 ± 6.5 | 15.4 ± 5.6 |
| **uACR median** | 0.0 (0.0, 1.3) | 13.1 (4.2, 47.3) | 18.3 (5.8, 62.5) | 37.3 (14.3, 88.9) | 68.4 (31.6, 153.4) | 102.6 (39.0, 239.2) |
| **Sex, n(%)** |  |  |  |  |  |  |
| **Male** | 10,750 (45) | 117 (66) | 170 (72) | 52 (67) | 62 (70) | 81 (74) |
| **Female** | 13,050 (55) | 59 (34) | 66 (28) | 26 (33) | 27 (30) | 29 (26) |
| **Multimorbidity** |  |  |  |  |  |  |
| **n(%)** | 14,462 (61) | 131 (74) | 182 (77) | 64 (82) | 70 (79) | 89 (81) |
| **Mean LTC count** | 2.1 ± 1.5 | 2.4 ± 1.4 | 2.7 ± 1.5 | 2.7 ± 1.5 | 2.7 ± 1.3 | 2.7 ± 1.3 |
| **Kidney failure within 2 years** | 65 (0) | 5 (3) | 10 (4) | 8 (10) | 17 (19) | 45 (41) |
| **Kidney failure within 5 years** | 117 (0) | 12 (7) | 35 (15) | 23 (29) | 44 (49) | 81 (74) |
| **Death within 2 years** | 443 (2) | 10 (6) | 17 (7) | 4 (5) | 11 (12) | 14 (13) |
| **Death within 5 years** | 1,349 (6) | 22 (12) | 42 (18) | 9 (12) | 21 (24) | 28 (25) |
| **Sensitivity (recall)** | 33.4 | 3.9 | 11.5 | 7.5 | 14.4 | 26.6 |
| **PPV** | 0.5 | 6.8 | 14.8 | 29.5 | 49.4 | 73.6 |

Supplementary Table 15. Individuals, characteristics, event numbers and performance for each risk group calculated by 5-year KFRE in UK Biobank

|  | **KFRE risk groups (as calculated by 5-year KFRE) – SCREAM** | | | | | |
| --- | --- | --- | --- | --- | --- | --- |
|  | **<3%**  **n = 36181** | **3- <5%**  **n = 1561** | **5- <15%**  **n = 2541** | **15- <25%**  **n = 828** | **25- <50%**  **n = 1000** | **≥50%**  **n= 791** |
| **Age** | 70.1 ± 14.0 | 81.8 ± 13.5 | 71.7 ± 13.9 | 71.7 ± 12.8 | 69.4 ± 15.1 | 62.2 ± 17.1 |
| **eGFRcr mean (SD)** | 55.4 ± 18.0 | 34.1 ± 7.0 | 28.7 ± 6.9 | 22.8 ± 5.6 | 18.5 ± 5.7 | 12.6 ± 4.8 |
| **eGFRcys mean (SD)** | 49.2 ± 19.1 | 33.2 ± 13.2 | 27.6 ± 11.1 | 22.3 ± 7.9 | 18.9 ± 7.5 | 14.5 ± 4.9 |
| **uACR median** | 0.4 (0.0, 3.3) | 8.9 (4.2, 38.1) | 18.6 (7.6, 40.2) | 38.18 (12.2, 75.0) | 38.1 (23.1, 138.9) | 138.9 (38.1, 141.9) |
| **Sex, n(%)** |  |  |  |  |  |  |
| **Male** | 13,292 (37) | 307 (20) | 468 (18) | 138 (17) | 194 (19) | 153 (19) |
| **Female** | 22,889 (63) | 1,254 (80) | 2,073 (82) | 690 (83) | 806 (81) | 638 (81) |
| **Multimorbidity** |  |  |  |  |  |  |
| **n(%)** | 25,068 (69) | 1,220 (78) | 1,943 (76) | 658 (79) | 734 (73) | 524 (66) |
| **Mean LTC count** | 2.6 ± 1.8 | 3.0 ± 1.8 | 3.0 ± 1.8 | 3.1 ± 1.8 | 2.8 ± 1.8 | 2.5 ± 1.8 |
| **Kidney failure within 2 years** | 147 (0) | 17 (1) | 89 (4) | 63 (8) | 193 (19) | 409 (52) |
| **Kidney failure within 5 years** | 202 (1) | 27 (2) | 115 (5) | 82 (10) | 235 (24) | 437 (55) |
| **Death within 2 years** | 6,417 (18) | 424 (27) | 804 (32) | 320 (39) | 451 (45) | 369 (47) |
| **Death within 5 years** | 7,469 (21) | 487 (31) | 930 (37) | 369 (45) | 503 (50) | 394 (50) |
| **Sensitivity (recall)** | 18.4 | 2.5 | 10.5 | 7.5 | 21.4 | 39.8 |
| **PPV** | 0.6 | 1.7 | 4.5 | 9.9 | 23.5 | 55.2 |

Supplementary Table 16. Individuals, characteristics, event numbers and performance for each risk group calculated by 5-year KFRE in SCREAM

|  | **KFRE risk groups (as calculated by updated KFRE accounting for competing mortality risk) – UK Biobank** | | | | | |
| --- | --- | --- | --- | --- | --- | --- |
|  | **<3%**  **n = 22897** | **3- <5%**  **n = 539** | **5- <15%**  **n = 599** | **15- <25%**  **n = 149** | **25- <50%**  **n = 157** | **≥50%**  **n= 148** |
| **Age** | 63.0 ± 5.4 | 61.4 ± 7.0 | 61.0 ± 7.4 | 59.8 ± 7.8 | 58.7 ± 7.6 | 58.6 ± 7.6 |
| **eGFRcr mean (SD)** | 67.6 ± 14.3 | 46.0 ± 6.7 | 40.1 ± 6.9 | 31.6 ± 6.5 | 26.0 ± 6.2 | 15.3 ± 6.8 |
| **eGFRcys mean (SD)** | 56.1 ± 11.9 | 47.6 ± 15.5 | 39.4 ± 13.6 | 30.7 ± 11.1 | 16.1 ± 8.7 | 17.4 ± 6.6 |
| **uACR median** | 0.0 (0.0, 1.2) | 2.7 (0.8, 9.6) | 5.4 (1.8, 24.0) | 14.7 (2.9, 56.2) | 20.8 (5.2, 78.2) | 57.0 (22.5, 146.4) |
| **Sex, n(%)** |  |  |  |  |  |  |
| **Male** | 12,989 (57) | 90 (17) | 97 (16) | 31 (21) | 31 (20) | 19 (13) |
| **Female** | 9,908 (43) | 449 (83) | 502 (84) | 118 (79) | 126 (80) | 129 (87) |
| **Multimorbidity** |  |  |  |  |  |  |
| **n(%)** | 13,822 (60) | 387 (72) | 438 (73) | 109 (73) | 120 (76) | 122 (82) |
| **Mean LTC count** | 2.1 ± 1.5 | 2.5 ± 1.5 | 2.5 ± 1.5 | 2.5 ± 1.5 | 2.6 ± 1.4 | 2.6 ± 1.2 |
| **Kidney failure within 2 years** | 43 (0) | 7 (1) | 18 (3) | 7 (5) | 26 (17) | 49 (33) |
| **Kidney failure within 5 years** | 80 (0) | 11 (2) | 47 (8) | 26 (17) | 52 (33) | 96 (65) |
| **Death within 2 years** | 404 (2) | 26 (5) | 33 (6) | 3 (2) | 18 (11) | 15 (10) |
| **Death within 5 years** | 1,250 (5) | 57 (11) | 82 (14) | 17 (11) | 35 (22) | 30 (20) |
| **Sensitivity (recall)** | 25.6 | 4.5 | 15.1 | 8.3 | 16.7 | 30.8 |
| **PPV** | 0.3 | 2.0 | 7.8 | 17.4 | 33.1 | 64.9 |

Supplementary Table 17. Individuals, characteristics, event numbers and performance for each risk group calculated by 5-year updated KFRE accounting for competing mortality risk in UK Biobank

|  | **KFRE risk groups (as calculated by updated KFRE accounting for competing mortality risk) – SCREAM** | | | | | |
| --- | --- | --- | --- | --- | --- | --- |
|  | **<3%**  **n = 35189** | **3- <5%**  **n = 2428** | **5- <15%**  **n = 3202** | **15- <25%**  **n = 857** | **25- <50%**  **n = 733** | **≥50%**  **n= 493** |
| **Age** | 70.9 ± 13.7 | 69.6 ± 14.1 | 68.5 ± 14.5 | 65.9 ± 14.4 | 61.4 ± 14.2 | 49.0 ± 15.5 |
| **eGFRcr mean (SD)** | 55.9 ± 17.9 | 33.6 ± 9.6 | 27.8 ± 9.5 | 22.2 ± 9.2 | 18.6 ± 8.4 | 15.0 ± 7.1 |
| **eGFRcys mean (SD)** | 49.5 ± 18.8 | 33.5 ± 16.6 | 27.2 ± 13.9 | 21.4 ± 9.9 | 18.9 ± 8.6 | 16.2 ± 6.4 |
| **uACR median** | 0.40 (0.00, 3.80) | 6.69 (1.30, 31.02) | 10.10 (3.50, 38.08) | 38.08 (7.57, 101.89) | 38.08 (13.09, 138.88) | 38.08 (33.60, 138.88) |
| **Sex, n(%)** |  |  |  |  |  |  |
| **Male** | 12,393 (35) | 602 (25) | 820 (26) | 276 (32) | 236 (32) | 225 (46) |
| **Female** | 22,796 (65) | 1,826 (75) | 2,382 (74) | 581 (68) | 497 (68) | 268 (54) |
| **Multimorbidity** |  |  |  |  |  |  |
| **n(%)** | 24,620 (70) | 1,781 (73) | 2,392 (75) | 607 (71) | 490 (67) | 257 (52) |
| **Mean LTC count** | 2.6 ± 1.8 | 2.8 ± 1.8 | 2.9 ± 1.9 | 2.7 ± 1.9 | 2.5 ± 1.8 | 2.0 ± 1.7 |
| **Kidney failure within 2 years** | 64 (0) | 41 (2) | 149 (5) | 134 (16) | 240 (33) | 290 (59) |
| **Kidney failure within 5 years** | 97 (0) | 56 (2) | 191 (6) | 165 (19) | 280 (38) | 309 (63) |
| **Death within 2 years** | 6,457 (18) | 608 (25) | 1,010 (32) | 300 (35) | 278 (38) | 132 (27 |
| **Death within 5 years** | 7,529 (21) | 685 (28) | 1,152 (36) | 339 (40) | 308 (42) | 139 (28 |
| **Sensitivity (recall)** | 8.8 | 5.1 | 17.4 | 15.0 | 25.5 | 28.1 |
| **PPV** | 0.3 | 2.3 | 6.0 | 19.3 | 38.2 | 62.7 |

Supplementary Table 18. Individuals, characteristics, event numbers and performance for each risk group calculated by 5-year updated KFRE accounting for competing mortality risk in SCREAM

|  | UK Biobank | | SCREAM | |  |
| --- | --- | --- | --- | --- | --- |
|  | KFRE  n=513 | Competing risk model  n=1053 | KFRE  n=5160 | Competing risk model  n=5285 |  |
| Age (years) | 60 (7) | 60 (8) | 70 (15) | 65 (16) |  |
| Sex n(%) |  | | | |  |
| Male | 365 (71.2) | 875 (83.1) | 4,207 (81.5) | 3,728 (70.5) |  |
| Female | 148 (28.8) | 178 (16.9) | 953 (18.5) | 1,577 (29.4) |  |
| eGFR creatinine (ml/min/1.73m2) | 26 (10) | 33 (11) | 23 (9) | 24 (10) |  |
| uACR (mg/mmol) | 96 (160) | 53 (120) | 68 (87) | 55 (83) |  |
| Age categories (years) |  |  |  |  |  |
| 18 to 44 | 17 (3.3) | 49 (4.7) | 394 (7.6) | 635 (12.0) |  |
| 45 to 64 | 319 (62.2) | 609 (57.8) | 1,091 (21.1) | 1,439 (27.2) |  |
| 65 to 74 | 177 (34.5) | 395 (37.5) | 1,416 (27.4) | 1,546 (29.3) |  |
| 75 plus | N/A | N/A | 2259 (43.8) | 1665 (31.5) |  |
| LTC count | | | | | |
| 0 | 12 (2.3) | 37 (3.5) | 421 (8.2) | 541 (10.2) |  |
| 1 | 96 (18.7) | 227 (21.6) | 880 (17.1) | 998 (18.9) |  |
| 2 | 150 (29.2) | 310 (29.4) | 1,100 (21.3) | 1,124 (21.3) |  |
| 3 | 126 (24.6) | 243 (23.1) | 1,042 (20.2) | 999 (18.9) |  |
| 4 | 72 (14.0) | 127 (12.1) | 742 (14.4) | 700 (13.2) |  |
| 5 or more | 57 (11.1) | 109 (10.4) | 975 (18.9) | 923 (17.5) |  |
| Multimorbidity clusters |  | | | |  |
| Cardiometabolic | 218 (42.5) | 439 (41.7) | 2,781 (53.9) | 2,574 (48.7) |  |
| Mixed mental and physical | 47 (9.2) | 80 (7.6) | 707 (13.7) | 760 (14.4) |  |
| Complex | 189 (36.8) | 334 (31.7) | 2,041 (39.6) | 1,983 (37.5) |  |
| Event within 5 years |  | | | |  |
| Kidney failure | 183 (35.7) | 221 (21.0) | 869 (16.8) | 945 (17.9) |  |
| Death | 100 (19.5) | 164 (15.6) | 2,196 (42.6) | 1,938 (36.7) |  |

Supplementary Table 19. Baseline characteristics of individuals with predicted risk of kidney failure >5% by KFRE or competing risk model

| eGFR equation used in KFRE | Performance measure for 2-year KFRE (95% CI) | Whole cohort | Multimorbidity | No multimorbidity |
| --- | --- | --- | --- | --- |
| eGFRcr | AUC | 0.92 (0.90-0.93) | 0.90 (0.88-0.93) | 0.94 (0.92-0.97) |
|  | C-index | 0.93 (0.92-0.94) | 0.92 (0.91-0.94) | 0.95 (0.94-0.97) |
|  | O/E ratio | 0.83 (0.75-0.92) | 0.83 (0.72-0.93) | 0.85 (0.70-1.00) |
|  | Calibration intercept | -0.07 (-0.16-0.03) | -0.07 (-0.20-0.05) | -0.06 (-0.23-0.10) |
|  | Calibration slope | 1.35 (1.21-1.49) | 1.32 (1.15-1.48) | 1.43 (1.18-1.68) |
|  | Brier score | 0.009 (0.008-0.010) | 0.010 (0.009-0.010) | 0.008 (0.007-0.010) |
|  | Scaled brier score (%) | 32.48 (29.16-35.59) | 28.85 (24.82-32.60) | 40.38 (33.83-46.28) |
| eGFRcys | AUC | 0.92 (0.90-0.93) | 0.90 (0.88-0.92) | 0.95 (0.92-0.97) |
|  | C-index | 0.94 (0.93-0.94) | 0.92 (0.91-0.93) | 0.96 (0.95-0.97) |
|  | O/E ratio | 0.65 (0.56-0.74) | 0.58 (0.48-0.69) | 0.87 (0.72-1.02) |
|  | Calibration intercept | -0.23 (-0.32- -0.13) | -0.34 (-0.45- -0.22) | 0.06 (-0.10-0.21) |
|  | Calibration slope | 1.49 (1.34-1.64) | 1.47 (1.29-1.65) | 1.48 (1.22-1.73) |
|  | Brier score | 0.010 (0.009-0.010) | 0.010 (0.009-0.011) | 0.009 (0.007-0.010) |
|  | Scaled brier score (%) | 29.14 (25.91-28.65) | 24.69 (20.63-28.65) | 38.28 (32.87-42.99) |
| eGFRcrcys | AUC | 0.92 (0.90-0.94) | 0.91 (0.88-0.93) | 0.95 (0.92-0.97) |
|  | C-index | 0.94 (0.93-0.95 | 0.92 (0.91-0.94) | 0.96 (0.95-0.97) |
|  | O/E ratio | 0.80 (0.72-0.89) | 0.76 (0.66-0.87) | 0.92 (0.77-1.07) |
|  | Calibration intercept | -0.04 (-0.14-0.05) | -0.09 (-0.21-0.03) | 0.07 (-0.09-0.23) |
|  | Calibration slope | 1.45 (1.30-1.60) | 1.44 (1.26-1.62) | 1.44 (1.18-1.70) |
|  | Brier score | 0.010 (0.009-0.010) | 0.010 (0.009-0.011) | 0.009 (0.007-0.010) |
|  | Scaled brier score (%) | 29.14 (30.09-36.12) | 24.69 (25.63-33.07) | 38.28 (35.51-46.77) |

Supplementary Table 20. Sensitivity analysis: Performance measures for SCREAM cohort by eGFR equation and multimorbidity status using Non-North American calibrated 2-year KFRE

AUC = Area under the receiver operator characteristic curves

| eGFR equation used in KFRE | Performance measure for 5-year KFRE | Whole cohort | Multimorbidity | No multimorbidity |
| --- | --- | --- | --- | --- |
| eGFRcr | AUC | 0.88 (0.87-0.90) | 0.88 (0.86-0.90) | 0.89 (0.86-0.91) |
|  | C-index | 0.92 (0.91-0.93) | 0.91 (0.90-0.92 | 0.94 (0.93-0.95) |
|  | O/E ratio | 0.82 (0.76-0.88) | 0.79 (0.72-0.87) | 0.89 (0.79-0.99) |
|  | Calibration intercept | -0.42 (-0.51- -0.34) | -0.45 (-0.56- -0.35) | -0.35 (-0.50- -0.20) |
|  | Calibration slope | 1.00 (0.92-1.07) | 1.00 (0.91-1.10) | 0.94 (0.82-1.05) |
|  | Brier score | 0.023 (0.022-0.025) | 0.023 (0.021-0.025) | 0.023 (0.020-0.025) |
|  | Scaled brier score (%) | 35.28 (31.84-38.55) | 34.57 (30.51-38.28) | 37.23 (31.46-42.88) |
| eGFRcys | AUC | 0.88 (0.87-0.90) | 0.87 (0.86-0.89) | 0.90 (0.87-0.92) |
|  | C-index | 0.93 (0.92-0.93) | 0.92 (0.91-0.92) | 0.95 (0.94-0.95) |
|  | O/E ratio | 0.81 (0.75-0.87) | 0.73 (0.65-0.80) | 1.12 (1.02-1.22) |
|  | Calibration intercept | -0.71 (-0.79- -0.63) | -0.84 (-0.93- -0.74) | -0.31 (-0.45- -0.18) |
|  | Calibration slope | 1.02 (0.95-1.09) | 1.02 (0.94-1.11) | 0.99 (0.87-1.10) |
|  | Brier score | 0.024 (0.023-0.026) | 0.025 (0.024-0.027) | 0.023 (0.020-0.025) |
|  | Scaled brier score (%) | 32.17 (28.42-35.45) | 28.71 (24.27-33.06) | 38.16 (31.77-43.77) |
| eGFRcrcys | AUC | 0.89 (0.87-0.90) | 0.88 (0.87-0.90) | 0.90 (0.87-0.92) |
|  | C-index | 0.93 (0.92-0.94) | 0.92 (0.91-0.93) | 0.95 (0.94-0.96) |
|  | O/E ratio | 0.77 (0.72-0.83) | 0.72 (0.64-0.79) | 0.95 (0.84-1.05) |
|  | Calibration intercept | -0.44 (-0.52- -0.36) | -0.53 (-0.63- -0.43) | -0.22 (-0.36- -0.07) |
|  | Calibration slope | 1.01 (0.93-1.08) | 1.01 (0.92-1.10) | 0.96 (0.84-1.08) |
|  | Brier score | 0.022 (0.021-0.024) | 0.023 (0.021-0.025) | 0.021 (0.019-0.024) |
|  | Scaled brier score (%) | 37.77 (34.66-40.70) | 36.13 (32.12-39.93) | 41.08 (35.27-46.22) |

Supplementary Table 21. Sensitivity analysis: Performance measures for SCREAM cohort by eGFR equation and multimorbidity status using Non-North American calibrated 5-year KFRE

AUC = Area under the receiver operator characteristic curves

| eGFR equation used in KFRE | Performance measure for 2-year KFRE (95% CI) | Multimorbidity cluster | |
| --- | --- | --- | --- |
|  |  | **Cardiometabolic**  **n=17854** | **Complex**  **n=16330** |
| eGFRcr | AUC | 0.90 (0.87-0.93) | 0.93 (0.91-0.95) |
|  | C-index | 0.92 (0.91-0.93) | 0.93 (0.91-0.94) |
|  | O/E ratio | 0.84 (0.71-0.96) | 1.03 (0.88-1.19) |
|  | Calibration intercept | -0.05 (-0.19-0.10) | -0.13 (-0.31-0.05) |
|  | Calibration slope | 1.31 (1.11-1.51) | 1.37 (1.11-1.63) |
|  | Brier score | 0.012 (0.010-0.013) | 0.009 (0.007-0.010) |
|  | Scaled brier score (%) | 28.75 (24.24-33.56) | 27.27 (21.66-32.75) |
| eGFRcys | AUC | 0.90 (0.87-0.93) | 0.93 (0.91-0.95) |
|  | C-index | 0.92 (0.91-0.93) | 0.92 (0.90-0.94) |
|  | O/E ratio | 0.59 (0.46-0.71) | 0.65 (0.49-0.80) |
|  | Calibration intercept | -0.34 (-0.48- -0.20) | -0.49 (-0.67- -0.32) |
|  | Calibration slope | 1.34 (1.15-1.53) | 1.58 (1.29-1.87) |
|  | Brier score | 0.012 (0.011-0.014) | 0.009 (0.008-0.010) |
|  | Scaled brier score (%) | 23.93 (19.27-28.25) | 22.00 (14.84-27.54) |
| eGFRcrcys | AUC | 0.90 (0.88-0.93) | 0.93 (0.88-0.91) |
|  | C-index | 0.92 (0.91-0.94) | 0.93 (0.91-0.94) |
|  | O/E ratio | 0.77 (0.64-0.89) | 0.90 (0.74-1.05) |
|  | Calibration intercept | -0.08 (-0.22-0.06) | -0.20 (-0.37- -0.02) |
|  | Calibration slope | 1.38 (1.18-1.59) | 1.52 (1.23-1.80) |
|  | Brier score | 0.012 (0.010-0.013) | 0.008 (0.007-0.010) |
|  | Scaled brier score (%) | 28.95 (24.41-32.89) | 27.99 (22.60-33.07) |

Supplementary Table 22. Sensitivity analysis: Performance measures for SCREAM cohort by eGFR equation and multimorbidity clusters using Non-North American calibrated 2-year KFRE

AUC = Area under the receiver operator characteristic curves

| eGFR equation used in KFRE | Performance measure for 5-year KFRE (95% CI) | Multimorbidity cluster | |
| --- | --- | --- | --- |
|  |  | **Cardiometabolic**  **n=17854** | **Complex**  **n=16330** |
| eGFRcr | AUC | 0.87 (0.85-0.90) | 0.87 (0.84-0.89) |
|  | C-index | 0.91 (0.90-0.92) | 0.91 (0.90-0.93) |
|  | O/E ratio | 0.76 (0.67-0.85) | 1.04 (0.93-1.15) |
|  | Calibration intercept | -0.49 (-0.61- -0.37) | -0.51 (-0.66- -0.36) |
|  | Calibration slope | 1.01 (0.90-1.12) | 0.96 (0.83-1.08) |
|  | Brier score | 0.027 (0.024-0.029) | 0.023 (0.020-0.025) |
|  | Scaled brier score (%) | 34.58 (29.92-39.85) | 31.42 (26.00-36.99) |
| eGFRcys | AUC | 0.87 (0.85-0.89) | 0.86 (0.84-0.89) |
|  | C-index | 0.91 (0.90-0.92) | 0.91 (0.90-0.93) |
|  | O/E ratio | 0.69 (0.60-0.78) | 0.64 (0.53-0.75) |
|  | Calibration intercept | -0.85 (-0.96- -0.74) | -0.98 (-1.12—0.85) |
|  | Calibration slope | 1.05 (0.94-1.16) | 1.03 (0.91-1.16) |
|  | Brier score | 0.028 (0.026-0.031) | 0.025 (0.023-0.028) |
|  | Scaled brier score (%) | 31.27 (25.54-36.19) | 23.71 (16.29-30.49) |
| eGFRcrcys | AUC | 0.88 (0.86-0.90) | 0.87 (0.84-0.90) |
|  | C-index | 0.92 (0.91-0.92) | 0.92 (0.91-0.93) |
|  | O/E ratio | 0.68 (0.59-0.77) | 0.89 (0.78-1.00) |
|  | Calibration intercept | -0.55 (-0.67- -0.44) | -0.63 (-0.78- -0.49) |
|  | Calibration slope | 1.04 (0.93-1.15) | 0.98 (0.85-1.10) |
|  | Brier score | 0.026 (0.023-0.028) | 0.022 (0.020-0.025) |
|  | Scaled brier score (%) | 37.53 (32.87-41.97) | 32.54 (26.83-37.95) |

Supplementary Table 23. Sensitivity analysis: Performance measures for SCREAM cohort by eGFR equation and multimorbidity clusters using Non-North American calibrated 5-year KFRE

AUC = Area under the receiver operator characteristic curves

| Performance measure for 2-year KFRE | Whole cohort | | Multimorbidity | | No multimorbidity | |
| --- | --- | --- | --- | --- | --- | --- |
|  | **UK Biobank** | **SCREAM** | **UK Biobank** | **SCREAM** | **UK Biobank** | **SCREAM** |
| Precision | 0.26 | 0.27 | 0.27 | 0.24 | 0.22 | 0.34 |
| Recall | 0.45 | 0.71 | 0.46 | 0.70 | 0.38 | 0.75 |
| F1 score | 0.33 | 0.39 | 0.34 | 0.36 | 0.28 | 0.46 |
| AUPRC | 0.24 (0.17-0.32) | 0.46 (0.42-0.50) | 0.24 (0.17-0.34) | 0.41 (0.37-0.45) | 0.25 (0.08-0.40) | 0.55 (0.50-0.61) |

Supplementary Table 24. Sensitivity analysis: Additional performance measures for 2-year KFRE (utilising eGFRcr) by cohort and multimorbidity subgroups

AUPRC = Area under the precision-recall curve

| Performance measure for 5-year KFRE | Whole cohort | | Multimorbidity | | No multimorbidity | |
| --- | --- | --- | --- | --- | --- | --- |
|  | **UK Biobank** | **SCREAM** | **UK Biobank** | **SCREAM** | **UK Biobank** | **SCREAM** |
| Precision | 0.36 | 0.17 | 0.35 | 0.15 | 0.39 | 0.23 |
| Recall | 0.59 | 0.81 | 0.58 | 0.80 | 0.60 | 0.83 |
| F1 score | 0.44 | 0.28 | 0.44 | 0.25 | 0.47 | 0.36 |
| AUPRC | 0.45 (0.38-0.51) | 0.46 (0.43-0.49) | 0.44 (0.38-0.52) | 0.41 (0.37-0.45) | 0.47 (0.34-0.59) | 0.56 (0.51-0.61) |

Supplementary Table 25. Sensitivity analysis: Additional performance measures for 5-year KFRE (utilising eGFRcr) by cohort and multimorbidity subgroup

AUPRC = Area under the precision-recall curve

Supplementary Text 1 – Proteinuria conversion algorithm

Predicted urine ACR from urine PCR (uPCR) = exp(5.3920 +0.3072 x log(min(uPCR/50, 1)) + 1.5793 x log (max(min(uPCR/500, 100), 0.1) + 1.1266 x log (max(uPCR/500, 1)))

Predicted urine ACR from dipstick proteinuria = exp(2.43738 + 0.7539 x (if trace) + 1.7243 (if +) + 3.3475 x (if ++) + 4.6399 x (if >++))

Supplementary Text 2 - Interpretation of statistical performance measures utilised

Discrimination

Discrimination describes the ability of a prognostic model to correctly separate those who do or do not experience the outcome event. It is commonly assessed by the area under the receiver operating characteristic curve (AUC) and/or Harrel’s C-index. Harrell’s c-index measures overall discrimination by assessing the proportion of all comparable pairs where the model correctly predicts which individual experiences the event first. However, does not explicitly account for time. In comparison, the time-dependent AUC considers changes in risk over time. It evaluates discrimination at specific time points, allowing assessment of how well a prediction model distinguishes between outcomes at different time horizons. Discrimination (using both measures) range from 0.5 (no discrimination) to 1 (perfect discrimination).

Calibration

Calibration describes the agreement and correspondence between the predicted risk attributed from a prognostic model and the true observed risk that is measured and can be measured in a number of ways:

- Calibration curves - assess calibration over the whole range of predicted risk (0 to 1) by plotting the observed risks against the predicted risk (represented by the 45 degree line). Observation above the 45 degree represents under-estimation of risk and those below represent over-estimation of risk.
- Calibration intercept - assesses general calibration by assessing how close the estimated risks are to the overall observed outcome proportion. The ideal calibration intercept is 0, a positive intercept represents that the model systematically overestimates the outcome and a negative intercept indicates the mode underestimates the outcome.
- Calibration slope - measures the level of variation in model predictions across the range of predictions. The perfect calibration slope is 1, a slope <1 suggests that model predictions are too far apart/extreme (high predicted risks are too high and low predicted risks are too low) and a slope >1 indicates that the predictions are too modest (i.e. the high predicted risks are too low and the low predicted risks are too high).
- Calibration in the large: observed/expected (O/E) ratio - an overall measure of calibration. An O/E ratio of 1 indicates perfect calibration in the large (i.e. the number of observed events matches the number of expected events), a ratio >1 indicates that on average the model predictions are underestimated (i.e. there are more observed events than the model predicts), and a ratio <1 that the predictions are overestimated (the model predicts more outcome events than are observed).

Prediction error and model fit

- Brier Score - assesses how close the predicted probabilities are to observed outcomes. Brier Score can range from 0 (best) to 1 (worst).
- Scaled Brier Score - provides interpretation of the model against a null model without covariates. Scaled brier score can range from negative values (indicates predication from the model are worse than a null model) to 100% (indicates perfect predictive accuracy from the model). A scaled brier score of 0% would indicate that the predictions from a model are no better or worse than a null model.

Supplementary Text 3 – Description of Kaplan-Meier based calibration curve results for 2- and 5-year KFRE in UK Biobank and SCREAM cohorts

Calibration curves in UK Biobank revealed under-estimation at lower risk levels and over-estimation of risk at higher risk levels at 2-years for those with and without multimorbidity (Figure 1). Improved calibration of 2-year KFRE was evident in the SCREAM cohort in both those with and without multimorbidity but with under-estimation across most risk levels (Figure 2). In UK Biobank, there was under-estimation of risk at 5-years across most risk levels in patients with multimorbidity (Figure 1). In the no multimorbidity group in UK Biobank, at 5-years, there was underestimation at lower risk levels but overestimation at higher risk levels (Figure 1). In comparison, calibration at 5-years in SCREAM was good in those with and without multimorbidity (Figure 2).

Supplementary Text 4 – Description of competing risk analysis results

There was improved discrimination across both cohorts and multimorbidity statuses when measured by AUC (competing risk model; range 0.92-0.95, UK calibrated 5-year KFRE;0.88-0.90). However, discrimination measured by c-index showed similar results in both models. Calibration plots are presented in Supplementary Figures 17 and 18. Calibration-in-the-large (O/E ratios) were closer to 1 for the competing risk model, demonstrating improved calibration. Likewise, calibration intercepts were closer to 0 (ideal) for UK Biobank but not SCREAM. Demonstrating slight underestimation in the UK Biobank cohort and slight over-estimation in the SCREAM cohort but with similar results across multimorbidity status. Calibration slopes showed improvements (closer to 1) in the UK Biobank cohort but were similar in the SCREAM cohort compared to the non-competing risk 5-year KFRE.

Supplementary Text 5 – Sensitivity Analyses

Analyses validating the non-North American calibrated KFRE in the SCREAM cohort provided similar results (Supplementary Tables 15-18). Calibration in the large (as measured using O/E ratios) demonstrated worsening in the direction of over-estimation of the risk of kidney failure for all multimorbidity status groups and the cardiometabolic multimorbidity cluster, but remained unchanged for complex multimorbidity. The calibration intercept became negative and moved closer to 0 (ideal calibration) at 2-years in all groups. At 5-years KFRE calibration intercepts displayed increasingly negative values (systematic underestimation of risk). Overall fit assessed using the scaled Brier score showed similar results in the whole SCREAM cohort and multimorbidity group with the 2-year KFRE (all eGFR equations). Whereas small improvements were demonstrated in the no multimorbidity group compared to when the UK calibrated KFRE was used. Scaled Brier scores were similar for the 5-year KFRE using eGFRcr and eGFRcr-cys for all multimorbidity groupings but there were small reductions in the score when using eGFRcys, primarily for the whole cohort and multimorbidity group.

Calibration plots using a Fine and Gray approach were similar to those produced using a cause-specific hazards approach (Supplementary Figures 19-20).
